# Mechanistic insights into the plant biostimulant activity of a novel formulation based on rice husk nanobiosilica embedded in a seed coating alginate film

**DOI:** 10.3389/fpls.2024.1349573

**Published:** 2024-05-21

**Authors:** Naomi Tritean, Bogdan Trică, Ştefan-Ovidiu Dima, Luiza Capră, Raluca-Augusta Gabor, Anisoara Cimpean, Florin Oancea, Diana Constantinescu-Aruxandei

**Affiliations:** ^1^ National Institute for Research & Development in Chemistry and Petrochemistry—ICECHIM, Bucharest, Romania; ^2^ Faculty of Biology, University of Bucharest, Bucharest, Romania; ^3^ Faculty of Biotechnologies, University of Agronomic Sciences and Veterinary Medicine of Bucharest, Bucharest, Romania

**Keywords:** biogenic silica nanoparticles, alginate film coating, mixture design, Wurster process, mung seedlings, metabolic activity, ROS scavenging, salt stress

## Abstract

Seed coating ensures the targeted delivery of various compounds from the early stages of development to increase crop quality and yield. Silicon and alginate are known to have plant biostimulant effects. Rice husk (RH) is a significant source of biosilica. In this study, we coated mung bean seeds with an alginate–glycerol–sorbitol (AGS) film with embedded biogenic nanosilica (SiNPs) from RH, with significant plant biostimulant activity. After dilute acid hydrolysis of ground RH in a temperature-controlled hermetic reactor, the resulting RH substrate was neutralized and calcined at 650°C. The structural and compositional characteristics of the native RH, the intermediate substrate, and SiNPs, as well as the release of soluble Si from SiNPs, were investigated. The film for seed coating was optimized using a mixture design with three factors. The physiological properties were assessed in the absence and the presence of 50 mM salt added from the beginning. The main parameters investigated were the growth, development, metabolic activity, reactive oxygen species (ROS) metabolism, and the Si content of seedlings. The results evidenced a homogeneous AGS film formation embedding 50-nm amorphous SiNPs having Si–O–Si and Si–OH bonds, 0.347 cm^3^/g CPV (cumulative pore volume), and 240 m^2^/g SSA (specific surface area). The coating film has remarkable properties of enhancing the metabolic, proton pump activities and ROS scavenging of mung seedlings under salt stress. The study shows that the RH biogenic SiNPs can be efficiently applied, together with the optimized, beneficial alginate-based film, as plant biostimulants that alleviate saline stress from the first stages of plant development.

## 1 Introduction

The seeds require favorable conditions to germinate, and for proper development, they need nutrients and a stress-free environment. Unfortunately, the optimal seed germination and growing conditions have been diminishing, whereas the stress factors like drought, salinity, pollutants, and biotic factors have been increasing and diversifying (Tolisano and Del Buono, 2023).

Seed germination represents a cascade of morphological, physiological, and biochemical processes by which the seed embryo moves from a dormant state to an active state. The stages of the germination process include the imbibition process of seeds, with stimulation of the activity of various enzymes, the respiration process, the mobilization of seed reserve substances, and the outgrowth of the embryo from the soil (Srivastava, 2002). Both water uptake and respiration are intense at the beginning of the germination process because a significant increase in the water content of the seeds is necessary to maintain the high metabolic activities needed for proper germination. Therefore, it is very important to ensure a suitable environment for seed growth and development with improved crop quality and yield (Makhaye et al., 2021).

High salt concentration negatively affects the germination and the development of the seedlings, especially the root development, and the tolerance depends on the plant species and varieties (Hanumantharao et al., 2016). The reactive oxygen species (ROS) are significantly elevated due to the stress induced by salt. Salt can accumulate in the upper layer of the soil due to intensive evaporation of water during the dry season or from increased salinity of irrigation water. The impact of abiotic stress, such as salt stress, is amplified by climate change. Therefore, mitigation solutions to reduce the negative effects of high salt concentrations are needed, such as the use of plant biostimulants. Plant biostimulants are a class of products used for crop treatment, which have several agricultural functions, including increased crop tolerance to abiotic stress (Du Jardin, 2015; Regulation EU, 2019). Plant biostimulants enhance the sustainability of farming systems (Rouphael and Colla, 2020), optimizing the use of fertilizers (Halpern et al., 2015) and plant protection products (Pereira et al., 2021). Europe is the leading market for plant biostimulants, accounting for more than 40% of the world market share (Caradonia et al., 2019). The EU Regulation, which categorizes plant biostimulants among fertilizing products Regulation EU, 2019, entered into force in July 2022 and represents a framework that promotes sustainable and efficient utilization of plant biostimulants (Li et al., 2022). According to the standardized testing method under development, the effect of plant biostimulants must be demonstrated in the presence of abiotic stressors (Committees, 2021). Among biostimulants, there are various formulations based on biopolymers or bioactive nanoparticles such as silica nanoparticles lately reported, but less based on its combinations.

Alginate is a biopolymer well known for its excellent film-forming properties and is widely used in many industries such as biomedical, pharmaceutical, and food industries (Loureiro Dos Santos, 2017; Trica et al., 2019). Due to its ability to retain water, alginate seems to be an ideal candidate for the alleviation of drought and possibly salt stress and also for the slow release of various nutrients. Alginate, especially as alginate oligosaccharides, acts as an elicitor for plant defense mechanisms by modulating the level of stress-related hormones. This further activates a cascade of events that ultimately causes the upregulation of the genes involved in defense, antioxidant activities, plant interactions with organisms, growth, and development (Liu et al., 2013; Spadari et al., 2017; Salachna et al., 2018; Zhang et al., 2019; Saberi Riseh et al., 2022; Zhao et al., 2022). Alginate is a good candidate for coating films, including for seed coating, but for an optimal biocompatible filmogenic composition, proper plasticizers should be added, such as sorbitol and glycerol, which were used in this study.

Sorbitol is a sugar alcohol used as a plasticizer, which has been shown to have various effects, depending on dose, location, and context: it was shown to have and improve antioxidant activities in plants (Smirnoff and Cumbes, 1989; Cuin and Shabala, 2007; Singh et al., 2015; Di Stasio et al., 2018). Endogenous sorbitol has been correlated with plant tolerance to drought and/or salinity, being included in the list of polyols with osmoprotectant functions (Bohnert et al., 1995; Singh et al., 2015; Di Stasio et al., 2018), and the effects of exogenous sorbitol and other osmolytes strongly depend on the dose, mode of applications, and the presence or absence of abiotic stresses (Hasanuzzaman et al., 2019). Glycerol is another efficient plasticizer, and an osmolyte, better known as highly accumulating in algae under stress conditions (Sharma et al., 2014; Raymond et al., 2020). It is less studied as an osmolyte in plants, as only trace amounts were detected in higher plants (Gerber et al., 1988). Foliar application of glycerol improved salinity tolerance of maize plants (Kaya et al., 2013), activated the defense response in *Theobroma cacao* (Zhang et al., 2015), and had beneficial effects on ‘Chantenay’ carrot (*Daucus carota* L.) family Apiaceae, corn (*Zea mays* L.) family Poaceae, and spearmint (*Mentha spicata* L.) family Lamiaceae under normal conditions (Tisserat and Stuff, 2011). Exogenous glycerol was found to have dramatic effects on plants in some cases. Root application of glycerol affected the root development of *Arabidopsis* even at moderate concentrations (Hu et al., 2014).

Overall, sorbitol, in particular, and also glycerol can function endogenously as osmoprotectants in plants and algae, and when exogenously added at adequate doses, they can have beneficial effects (Sharma et al., 2014; Hasanuzzaman et al., 2019). They have been also used for osmopriming of seeds (Melekote Nagabhushan et al., 2022).

With respect to silicon (Si), most of its sources in soil are present as crystalline aluminosilicates, which are insoluble and not directly accessible to plants. The most efficient form of Si known to be available for uptake and translocation into the plant body is monosilicic acid (H_4_SiO_4_) (Richmond and Sussman, 2003). Nanoparticles (NPs) are used in agriculture for the targeted delivery of nutrients that are necessary for plant growth and development, the added amounts being low compared to those of macro-formulated fertilizers. The targeted delivery reduces the harmful environmental effect of chemical fertilizers and at the same time provides an increased uptake capacity by plant cells (Nafisi et al., 2015).

Silica nanoparticles (SiNPs) and SiO_2_xnH_2_O are some of the most efficient forms from which soluble Si species are constantly released in low concentrations (Bhat et al., 2021). Several recent studies indicated that some nanoparticles, including SiNPs, can also undergo uptake and translocation within the plant body, but the mechanisms are controversial (Kurczyńska et al., 2021; Pairault et al., 2022). Si is known to have two major functions in plants: a structural/mechanical function, which is associated with the translocation of Si through the apoplastic route, and a physiological function, which is associated with the translocation of Si through the symplastic route. In apoplast, it has a beneficial effect on plant protection (mechanical and physicochemical): increased resistance to pathogens and insects (Fauteux et al., 2006; Ferreira et al., 2015; Leroy et al., 2019), increased mechanical resistance (wind and intense rainfall and prevention of falling) (Guerriero et al., 2016; Luyckx et al., 2017), reduced effects of water stress (root development and increased turgescence) (Ali and Hassan, 2017; Verma et al., 2021), precipitation of heavy metals in apoplast, and reduction of phytotoxicity (Kim et al., 2014). In symplast, Si leads to plant biostimulation (physiological and biochemical) by priming and orchestrating plant defense pathways (Epstein, 1994); alleviating the symptoms of P, K, and Ca deficiency (Hu et al., 2021; Araújo et al., 2022); reducing the effects of moderate water stress (physiological drought and salinity) (Ali and Hassan, 2017; Avestan et al., 2019); and decreasing As (Khan et al., 2022), Cd (Farooq et al., 2013; Kabir et al., 2016), Al (De Jesus et al., 2017; Pontigo et al., 2017), Mn (Li et al., 2015), and Zn (Gu et al., 2012) toxicity. However, the mechanisms are not fully elucidated.

Under saline stress conditions, both alginate, especially as oligo-alginate, and SiNPs have been shown to act as plant biostimulants (Savvas and Ntatsi, 2015; Luyckx et al., 2017; Salachna et al., 2018; Zhu et al., 2019). Despite the fact that Si under various forms has been intensively studied, the effects of biogenic silica obtained from biomass have been less investigated on plants than other Si forms. The few studies available show promising results, and the biogenic SiNPs have similar properties to chemical SiNPs. Rice husk (RH) and rice husk ash (RHA) are good sources of silica, which were shown to release Si in soils more efficiently than other sources such as peat ash, coal ash, or ground granulated blast furnace slag (Hogan et al., 2019). RHA was reported to have positive effects on shoot and root biomass and chlorophyll content in chili plants (Dũng et al., 2016). RHA was recently shown to reduce the severity of the bacterial spot disease caused by *Xanthomonas vesicatoria* in sweet pepper plants (Awad-Allah et al., 2021) and had similar effects as sodium or calcium silicate, but less than Silixol on mango orchard (More et al., 2019). Nanosilica obtained from maize stalks increased the salt tolerance of Williams banana and improved its yield and quality (Ding et al., 2022). In-depth analyses comparing the biological activity of biogenic and chemical SiNPs are nevertheless lacking. In addition to pure SiNPs, macerates of biomass rich in Si have also yielded good results when applied to plants (Pairault et al., 2022).

The formulation of biostimulants as seed coating ensures a beneficial environment from the early stages of development (Qiu et al., 2020).

This study is focused on an alginate–glycerol–sorbitol (AGS) seed coating with embedded nanobiosilica from RH applied to mung seeds in the absence and presence of salt stress.

We obtained nanobiosilica from rice husk and embedded it in an optimized AGS film used for mung seed coating by a bottom-up (Wurster) spray process. We show that the coating alleviates salt-induced stress on mung beans from the first stages of seedling development.

## 2 Materials and methods

### 2.1 Materials

Rice husks (*Oryza sativa* cv. Keope) collected from a processor (Risso Scotti Danubio, Bucharest, Romania) were used as the source of biogenic silica nanoparticles. Mung bean (*Vigna radiata* L.) seeds were supplied by Amia International Otopeni, Romania, a local distributor of Vilmorin (Vilmorin, La Ménitré, France), and used as model seeds. The chemicals used in this study are presented in the **Supplementary Materials**.

### 2.2 Biogenic SiNP synthesis

Rice husks were ground using an electric herb grinder. The dilute acid hydrolysis of rice husks was carried out in a 5500 Parr hermetic reactor (Parr Instrument, Moline, IL, USA) with a capacity of 160 mL and heating power of 700 W and equipped with a Parr 4848 temperature controller and digital recording of the reaction parameters (T and p). The following parameters were used: 120°C ± 2°C, 14 ± 1 atm, 2 h, 90% of the reactor capacity volume, and a liquid/substrate (L/S) ratio of 5 (25 g of rice husk was mixed with 125 mL 0.1 N HCl) (Periasamy et al., 2018). After the hydrolysis reaction, the substrate was rinsed with double-distilled water until it reached neutral pH, and it was further dried overnight at 40°C. The final step involved the calcination of the dried substrate in a calcination furnace for 2 h at 650°C. The ash percent (w/w) was assessed using gravimetric analysis.

### 2.3 Physical–chemical analysis of RH intermediates and SiNPs

#### 2.3.1 Determination of chemical composition, constituents, and crystallinity

The C, N, and H contents were determined using a FlashSmart elemental analyzer (Thermo Fisher Scientific, Waltham, MA, USA) equipped with a thermal conductivity detector (TCD). The samples were burned at 950°C in an oxygen atmosphere (99.999% purity). A reference material, 2,5-bis(5-*tert*-butyl-2-benzo-oxazol-2-yl) (C = 72.52% ± 0.22%, N = 6.51% ± 0.09%, and H = 6.09% ± 0.08%), was used, with the ranges of the calibration curve being 0.008–0.172 mg for nitrogen (R^2^ = 0.9997), 0.089–1.914 mg for carbon (R^2^ = 0.9996), and 0.007–0.161 mg for hydrogen (R^2^ = 0.9995). Calibration curves were also checked for cysteine (C = 29.98% ± 0.28%, N = 11.66% ± 0.16%, and H = 5.03% ± 0.13%).

The amount of total Si was determined by X-ray fluorescence (XRF) spectroscopy, which was performed using an Olympus VANTA C spectrometer (Waltham, MA, USA) equipped with a 40-kV X-ray tube rhodium (Rh) anode, silicon drift detector (SSD), and 3-mm beam collimator.

##### 2.3.1.1 X-ray diffraction

X-ray diffraction (XRD) was performed using a Rigaku SmartLab diffractometer (Rigaku Corporation, Tokyo, Japan), operated at 40 kV and 200 mA emission current, using an incident Cu_Kα1_ radiation (λ = 1.54059 Å). The diffractograms were obtained continuously between 5° and 90°, with an extended interval compared to a similar study (Dubey et al., 2015), with a step of 0.02° and a scan speed of 4°/min. The crystallinity degree (XC), peak identification, and deconvolution were determined using the PDXL 2.7.2.0 software.

##### 2.3.1.2 Fourier transform infrared spectroscopy

Fourier transform infrared (FTIR) spectra were recorded in attenuated total reflectance (ATR) mode using IRTracer-100 FTIR (Shimadzu, Kyoto, Japan). The analyses were assessed by the acquisition of 45 scans with a resolution of 4 cm^−1^ in the mid-IR spectral range of 4,000–400 cm^−1^ (Dubey et al., 2015). The calibrations were conducted based on the methodology proposed by the manufacturer.

##### 2.3.1.3 Brunauer–Emmett–Teller

Morphological properties like specific surface area, specific pore volume, pore size (micro-, meso-, or macropores), and pore size distribution of biogenic SiNPs from rice husk were determined by nitrogen adsorption–desorption analyses on Autosorb-1 Quantachrome Nova 2200e Analyzer (Boynton Beach, FL, USA), using Type B long cell 9 mm with large bulb. Before N_2_ adsorption, the sample (0.0537 g) was degassed for 4 h under vacuum at 140°C, weighted before and after degassing, and measured under vacuum at 77 K (Rahman et al., 2009). Several available methods from the Quantachrome NovaWin version 11.03 software were employed to investigate the particularities of the sample: Brunauer–Emmett–Teller (BET), Barrett–Joyner–Halenda (BJH), non-local density functional theory (NL-DFT), t-method (t-plot), and Dubinin–Radushkevich (DR). The data reduction parameters were N_2_ adsorbate at 77.35 K, oxygen/zeolite adsorbent, thermal transpiration ON, de Boer method for T-method, ASILAR standard isotherm file, and NL-DFT equilibrium model for N_2_ at 77 K on silica cylindrical pores in the relative pressure range of 10^−7^–1. The main particularities of all the methods are reiterated in the **Supplementary Material**.

#### 2.3.2 Scanning electron microscopy–energy dispersive X-ray

The morphology of intermediates and elemental composition was investigated using a scanning electron microscope (SEM) Quanta 200 FEI (Amsterdam, The Netherlands) at 30-kV electron acceleration voltage, large field detector (LFD), and ×200 magnification (Dusevich et al., 2010). The elemental composition was assessed using an Octane Super 60-mm^2^ energy dispersive X-ray (EDX) detector (EDAX, Ametek Inc., Mahwah, NJ, USA).

#### 2.3.3 Transmission electron microscopy–EDX

The biogenic SiNPs were analyzed by transmission electron microscopy (TEM), and the elemental distribution was determined using the scanning transmission electron microscopy (STEM) mode coupled with the EDX detector X-MaxN 80T (Oxford Instruments, Abingdon, UK). TEM analysis was performed on Cu films coated with carbon film (Carbon Type-B, 200 mesh; Ted Pella, Newington, NH, USA) using a TECNAI F20 G2 TWIN Cryo-TEM transmission electron microscope (FEI, Austin, TX, USA) with an electron accelerating voltage of 200 kV. The grids were initially hydrophilized for 10 sec (model 1020; Fischione Plasma Cleaner, Export, PA, USA) in advanced vacuum and a binary gas atmosphere with O_2_ (25%)/Ar (75%) (Padgett et al., 2017). This procedure hydrophilizes the support based on carbon film and promotes the uniform deposition of nanoparticles. TEM analysis was performed in bright field (BF) mode.

#### 2.3.4 Dynamic light scattering analysis

Dynamic light scattering (DLS) analysis was assessed using AMERIGO™—Particle Size & Zeta potential Analyzer (Cordouan Technologies, Pessac, France), as per manufacturer’s instructions, using the high concentration head, which is based on the Dual Thickness Controller (DTC) technology. The analysis of the results was performed using the AmeriQ 3.2.3.0 software (more details can be found in Data Sheet 3 in the **Supplementary Material**).

#### 2.3.5 Quantification of the release of soluble silicon from SiNPs

To quantify the soluble Si released from SiNPs, 17.5 mL ultrapure water (SiNPs-W) or 50 mM NaCl solution prepared in ultrapure water (SiNPs-S) was poured over 18.25 mg SiNPs (the same ratio as in Section 2.4.4). The samples were analyzed both non-ultrasonicated (SiNPs-W and SiNPs-S) and ultrasonicated (SiNPs-Wu and SiNPs-Su) using a probe sonication (Sonics VCX 750, model CV334, Sonics & Materials, Newtown, CT, USA) with the following parameters: 20 kHz, 5 min, 80% amplitude, pulse 15 sec, and pause 30 sec (as in Section 2.4.2). The ultrasonication was performed at the beginning of the experiment. The released soluble silicon was assessed after ultracentrifugation of the samples at 400,000 rcf (CP100NX Ultracentrifuge, Hitachi Koki, Tokyo, Japan). The determination of soluble silicon was performed using the Supelco Silicate Test kit (1.00857.0001) (Uţoiu et al., 2018), with a concentration range of 0.5–500 mg/L Si (MQuant^®^Supelco, Merck, Darmstadt, Germany), according to the protocol provided by the manufacturer. The standard curve was carried out starting from a stock solution of 1,000 mg/L SiO_2_ (Supelco, Merck, Darmstadt, Germany).

### 2.4 Optimization and characterization of seed coating

#### 2.4.1 Design-Expert and dynamic mechanical analysis

A mixture design was applied with three factors, alginate, glycerol, and sorbitol, using the Design-Expert 11 software for the preparation of films (Trica et al., 2023). The films were prepared as described in **Supplementary Table 1**. After drying, the films were tested by dynamic mechanical analysis (DMA) Q800 (TA Instruments, New Castle, DE, USA). Tensile test specimens (L, length; W, width; δ, thickness) were obtained and mounted in the instrument. The traction tests that followed were performed in the following conditions: rectangular geometry, isothermal for 5 min, 30°C, and ramp force 0.2 N/min from 0.001 N to 18 N (Florian et al., 2018). The traction tests yielded Young’s modulus and the ultimate tensile strength (UTS) for each film.

#### 2.4.2 Dispersion of biogenic SiNPs

For the dispersion of SiNPs in alginate, we used probe sonication (Sonics VCX 750, model CV334, Sonics & Materials, Newtown, CT, USA) with the following parameters: 20 kHz, 5 min, 80% amplitude, pulse 15 sec, and pause 30 sec (Retamal Marín et al., 2017; Liu et al., 2022).

#### 2.4.3 Seed sterilization

The mung bean seeds were transferred to a sterile tube. In the first step, the seeds were sterilized with 95% ethanol for 3 min and gently mixed in the tube. After the ethanol removal, 5% sodium hypochlorite solution was added and stirred in the tube for another 3 min. After removing the hypochlorite solution, the seeds were washed with sterile double-distilled water (Sauer and Burroughs, 1986).

#### 2.4.4 Coating of mung bean seeds using the bottom-spray (Wurster) process

The coating process of the mung bean seeds was performed using a MINI-GLATT fluidized bed granulator (Glatt, Binzen, Germany) with some modifications compared to our previous work (Trica et al., 2023). A solution composed of 474.6 mg alginate, 175 mg glycerol, and 260.4 mg sorbitol in 35 mL sterilized double-distilled water was used, according to the optimum ratio between the three components found after the DMA/Design-Expert analysis. Five coating experimental variants were set up using 15 g of mung bean seeds and the abovementioned AGS solution, as follows: 1) Variant V0, AGS solution without SiNPs; 2) Variant V1, AGS solution + 1% SiNPs (w/w of total AGS organic substance); 3) Variant V2, AGS solution + 2% SiNPs (w/w); 4) Variant V3, AGS solution + 3% SiNPs (w/w); and 5) Variant V4, AGS solution + 4% SiNPs (w/w). The SiNP concentration was calculated as percent of the total weight of the organic fraction (910 mg) from the solution used for each coating. The film on each seed contained 1%, 2%, 3%, and 4% SiNPs.

As the process was carried out immediately after seed sterilization, a 2-min drying time of the seeds was set up in the granulator before coating. In addition to the five experimental coating variants, a control experiment with uncoated mung bean seeds, with water instead of the filmogenic solution spraying, was carried out. The settings of the granulator were a peristaltic pump solution flow rate of 4 mL/min, 2-bar spraying nozzle pressure, 40°C coating temperature, 45°C–50°C drying temperature, and 20–40 m^3^/h air volume for spray-drying. The parameters were adapted from our previously published study by modifying the debit of the pump, the nozzle pressure, and the air volume for spray-drying (Trica et al., 2023). Each coating took place for 15 min, representing the time required for all the AGS ± SiNP solution, or water in the case of control, to be sprayed inside the granulator. In the following, AGS film refers to the film without SiNPs.

#### 2.4.5 Water activity (aW) measurement

Water activity for the six experimental sets was measured (Baldet et al., 2009) using LabMaster-aw neo equipment (Novasina AG, Lachen, Switzerland), as per the manufacturer’s instructions. Three measurements were determined for each experimental set.

#### 2.4.6 SEM-EDX

SEM micrographs of uncoated and coated seeds were obtained using a TM4000Plus II tabletop electron microscope (Hitachi, Tokyo, Japan) at 5-kV electron acceleration voltage, backscattered-electron (BSE) detector, standard (M) vacuum mode, and ×600 magnification. The elemental composition was determined using an EDX detector AZtecLiveLite (Oxford Instruments, Abingdon, UK). The adjustments were made based on the methodology proposed by the manufacturer. The average width of the film was determined with the ImageJ software (Schindelin et al., 2012; Trica et al., 2023).

### 2.5 Plant biostimulant activity of the coating film

#### 2.5.1 Seed germination

Ten uncoated (C), AGS film-coated (V0) mung bean seeds, and mung bean seeds coated with the AGS film embedding different concentrations of SiNPs (V1, V2, V3, and V4) described in Section 2.4.4 were grown in both the absence and the presence of 50 mM NaCl. The seeds were placed in sterilized Magenta™ vessel GA-7 (Sigma-Aldrich, St. Louis, MO, USA), in which autoclaved 0.4% agar ± 50 mM NaCl had been previously poured and then set in a growth chamber with controlled light and temperature (ALGAETRON AG230, Photon Systems Instruments, Drásov, Czech Republic). The conditions for germination and seedling growth were set to a 16-h light/8-h dark cycle with 26°C temperature and 1,340 μE light intensity for the light period and 22°C temperature for the dark period (Trica et al., 2023). The experiment was stopped, and the seedlings were analyzed after 4 days after being placed into the agar medium. The analysis of the seedlings was performed at the end of the experiment (approx. 3-day-old seedlings): plant measurements, alpha-amylase activity, chlorophyll and carotenoid content, hydrogen peroxide (H_2_O_2_) content, l-proline content, nitric oxide (NO) content, lipid peroxidation [thiobarbituric acid reactive substance (TBARS)], activity of ROS scavenging enzymes, ROS detection and semi-quantitation in mung bean leaves using 2′,7′-dichlorodihydrofluorescein diacetate staining, and H_2_O_2_ detection in mung bean leaves using 3,3′-diaminobenzidine (DAB) staining. For the other analysis, the specific time point is specified in the corresponding subsection. Each experimental variant was tested in triplicate.

#### 2.5.2 Plant measurements

Mung bean seedlings were photographed, and the root and stem lengths were determined using the ImageJ software (Trica et al., 2023).

#### 2.5.3 Alpha-amylase activity

The investigation of α-amylase activity was performed following Hostettler et al. (2011), with each sample accompanied by a control in which the enzyme–substrate reaction was stopped by introducing the reagent before the substrate and was subjected to the same treatment. For the preparation of plant extracts, a solid:liquid ratio of 2:1 was used (2 g of fine powder was obtained by grounding the seedlings in liquid nitrogen and mixed with 1 mL of double-distilled water). The extracts were vortexed and centrifuged at 20,000 rcf and 4°C for 10 min, and the supernatant was incubated with the substrate (soluble 0.45% w/v starch solution in a 44 mM KH_2_PO_4 _+ 2 mM CaCl_2_ solution) in a 1:1 ratio. The samples were incubated for 10 min in a water bath at 30°C, and the reaction was stopped with Bernfeld reagent (Bernfeld, 1951) containing 1% 3,5-dinitrosalicylic acid in a 1:2 extract:reagent ratio. Subsequently, the samples were incubated in a digital dry bath (BSH1004, Benchmark Scientific, Sayreville, NJ, USA) at 95°C for 5 min. After cooling, the absorbance was read at 540 nm using a microplate reader (CLARIOstar BMG Labtech, Ortenberg, Germany) against a standard maltose curve prepared from a stock solution of 1 mg/mL maltose.

#### 2.5.4 Photosynthetic pigment analysis

Chlorophyll and carotenoid contents were determined using the equations described by Lichtenthaler and Buschmann (2001) for pure methanol solvent. Briefly, 50 mg of mung bean leaves was homogenized with 2 mL of pure methanol. The supernatant was read with a microplate reader for quantification of chlorophyll *a*, chlorophyll *b*, and carotenoids after centrifugation of the homogenate at 6,000 rcf for 15 min at 4°C.

#### 2.5.5 Medium acidification assay

The experiment was performed following Zandonadi et al. (2016) with some modifications regarding the agar concentration and the investigation of proton pump activity through image analysis. On the third day of seedling growth, a solution of 0.04 g/L bromocresol purple in double-distilled water was prepared and brought to pH 6.7 with 1 N NaOH solution. After the addition of agar to a final concentration of 0.75%, the solution was autoclaved and poured into sterile Petri dishes with a diameter of 90 mm. Mung bean seedlings were placed with the root partially embedded into the agar gel after the gel reached a temperature of approximately 30°C. The image acquisition was carried out after 24 h. The experimental variants were tested in triplicate. Semi-quantitative analysis of specific extracellular H^+^ level (seH^+^) was performed using the ImageJ software by measuring the area of the yellow region (adjust color threshold, make binary, and remove outliers) and the intensity of the yellow color (live histogram) and the root area and by applying the following formula:


$$se{\text{H}}^{+}=\frac{A_{y}\times I}{A_{r}},$$

where *A_y_* is the area of the yellow region, *I* is the color intensity of the yellow region, and *A_r_* is the root area. The total extracellular H^+^ level, i.e., without normalization to the root area, was also calculated.

#### 2.5.6 3-(4,5-Dimethylthiazol-2-yl)-2,5-diphenyltetrazolium bromide assay

The metabolic activity during germination was determined according to Pouvreau et al. (2013) with some modifications in order to adjust the experiment for larger seeds. The experiment was conducted in 24-well plates. Three seeds were placed in each well, and each experimental variant was performed in triplicate. For the experimental set without salt and the salt-stress set, 500 µL of sterile double-distilled water and 50 mM NaCl solution, respectively, were pipetted into each well. The 24-well plates were placed into the growth chamber for 24 h using the same parameters as in Section 2.5.1. Next, the seedlings were washed three times with double-distilled water and incubated in the dark with 770 µL of 0.45 mg/mL 3-(4,5-dimethylthiazol-2-yl)-2,5-diphenyltetrazolium bromide (MTT) solution for 24 h at 24°C. The solubilization of the tetrazolium salt was carried out by adding 1.4 mL of buffer prepared from 10% Triton X-100 and 0.04 M HCl in isopropanol and keeping the sealed plates for 24 h in the dark at 30°C and 100 rpm. Afterward, the liquid was transferred to 96-well plates and read at 570 and 630 nm. The absorbance at 630 nm was subtracted from the absorbance at 570 nm for turbidity correction.

#### 2.5.7 l-Proline content

l-Proline was assessed after a previously described method (Igiehon and Babalola, 2021) with minor modifications related to the ratio between the seedling and the sulfosalicylic acid, as well as to the centrifugation time. Briefly, 0.35 g of mung bean seedlings previously ground in liquid nitrogen was mixed with 5 mL of 3% sulfosalicylic acid. After 20 min of centrifugation at 1,520 rcf, 2 mL of supernatant was mixed with 2 mL of acid ninhydrin reagent and incubated at 100°C in a water bath (OLS Aqua Pro Shaking Water Bath, Grant Instruments, Cambridge, UK) for 1 h. The samples were transferred on ice for a few minutes, 4 mL of toluene was added, and after vigorous stirring, the samples were left to sit for 30 min in the dark for phase separation. The absorption of the organic phase was read at 520 nm (Ocean Optics UV-VIS-NIR, Orlando, FL, USA).

#### 2.5.8 Nitric oxide content

The NO content was determined according to Liu et al. (2007). Briefly, the mung bean seedlings were homogenized in 40 mM HEPES buffer (pH = 7.2) with a ratio of 1:10 (w/v). After the centrifugation of the homogenates at 5,000 rcf for 10 min at 4°C, the NO content was assessed using the Griess reagent system.

#### 2.5.9 Hydrogen peroxide content

Hydrogen peroxide content was determined according to Önder et al. (2020). Briefly, the seedlings frozen in liquid nitrogen were homogenized with 0.1% trichloroacetic acid (TCA). After centrifugation of the homogenate at 15,000 rcf for 15 min at 4°C, the supernatant was mixed with 10 mM K_2_HPO_4_ buffer, pH = 7, and 1 M KI. The reaction mix was incubated for 30 min at room temperature in the dark. Next, the samples were read at 390 nm using a microplate reader. The standard curve was subjected to the same steps starting from a stock solution of 9.8 mM H_2_O_2_.

#### 2.5.10 Detection of H_2_O_2_ in mung bean leaves using 3,3′-diaminobenzidine staining

The mung bean leaves were stained with DAB for the detection of H_2_O_2_ after a previously described method (Obrien, 2012).

#### 2.5.11 Lipid peroxidation assay (TBARS content)

Lipid peroxides were dosed using the TBARS method (Heath and Packer, 1968). After being frozen in liquid nitrogen, 0.1 g of mung bean seedling powder was mixed with 0.1% TCA at a solid/liquid ratio of 1:10. The extracts were vortexed and centrifuged at 20,000 rcf and 4°C for 10 min, and the supernatant was mixed with TBARS reagent (0.5% thiobarbituric acid in 20% trichloroacetic acid) at a 1:2 ratio. The samples were incubated at 95°C for 15 min in the BSH1004 digital dry bath. After cooling the samples on ice, the absorbance was read at 532 nm and 600 nm for turbidity correction. The standard curve was prepared from a stock solution of 200 µM MDA according to Aguilar Diaz De Leon and Borges (2020).

#### 2.5.12 Activity of ROS scavenging enzymes

The activity of key enzymes in ROS metabolism was determined according to Chen and Zhang (2016), with some modifications. For enzyme extraction, 0.25 g of mung bean seedlings previously frozen in liquid nitrogen and ground into a fine mixture was mixed with 2 mL of phosphate-buffered saline (PBS), pH 7.8. The samples were vortexed for 3 min and centrifuged at 20,000 rcf and 4°C for 20 min.

Briefly, for 1) superoxide dismutase (SOD) activity, the enzyme extract was mixed in a 1:19 ratio with the reaction mix consisting of 100 mM PBS, pH 7.8, 1 mM EDTA disodium salt, 130 mM methionine, 750 µM nitroblue tetrazolium (NBT), and 20 µM riboflavin. The mixture was transferred to the ALGAETRON light-controlled chamber at 74 µE for 9 min. The absorbance was measured using the microplate reader at 560 nm. The samples were followed by two controls in which PBS was added as enzyme extract replacement. One of the controls was subjected to light at the same time as the samples, and one control was kept in the dark. One unit of SOD activity was expressed as the amount of enzyme that inhibits 50% of NBT photoreduction. The SOD activity was expressed as units (U) per gram of seedling fresh weight instead of per mg protein.

For 2) guaiacol peroxidase (G-POX) activity, the enzyme extract was mixed in a 1:19 ratio with the reaction mix prepared by mixing 56 µL of 0.2% (v/v) guaiacol and 76 µL of 30% H_2_O_2_ stock solution in 100 mL of 100 mM PBS, pH 7. One unit of G-POX was expressed as the production of 1 nmol of tetra guaiacol/min (extinction coefficient ε = 26.6 mM^−1^ cm^−1^ at 470 nm). The absorbance increase at 470 nm was monitored for 5 min in the microplate reader.

For 3) catalase (CAT) activity, the enzyme extract was mixed in a 1:19 ratio with the reaction mix prepared by adding 155 µL of 30% H_2_O_2_ in 100 mL of 100 mM PBS, pH 7. One unit of CAT (µmol/min) was defined as the decomposition of 1 µmol H_2_O_2_/min, pH 7, 25°C. The decrease of absorbance at 240 nm was monitored for 5 min in the microplate reader. The extinction coefficient 43.6 M^−1^ cm^−1^ of H_2_O_2_ at 240 nm was considered for calculations.

For 4) glutathione reductase (GR) activity, the enzyme extract was prepared by mixing 0.15 g of mung bean seedlings previously frozen in liquid nitrogen with 1.5 mL of buffer (pH = 7.2, 50 mM Tris-HCl, 1 mM EDTA, 5% glycerol, and 5 mM MgCl_2_). The homogenates were centrifuged at 16,000 rcf for 20 min at 4°C. The reaction mix consisted of enzyme extract and buffer (pH = 7.2, 0.05 M Tris-HCl, 0.25 mM NADPH, 1 mM EDTA, and 1 mM oxidized glutathione) mixed in a 1:1 ratio. One unit of GR (nmol/min) was defined as the amount of GR that oxidizes 1 nmol of NADPH/min at 25°C. The absorbance was monitored for 20 min at 340 nm (Labudda et al., 2020).

#### 2.5.13 ROS detection in mung bean leaves using 2′,7′-dichlorodihydrofluorescein diacetate staining

2′,7′-Dichlorodihydrofluorescein diacetate (H_2_DCFDA) was used in order to visualize the intracellular ROS according to Georgescu et al. (2018). Briefly, the mung bean leaves were incubated in the dark at room temperature for 30 min with 2.5 μM H_2_DCFDA solution prepared in double-distilled water from a stock solution of 10 mM H_2_DCFDA in dimethyl sulfoxide (DMSO). Afterward, the leaves were washed with double-distilled water. Intracellular ROS were visualized using CELENA® X High Content Imaging System (Logos Biosystems, Annandale, VA, USA). Mean fluorescence intensity was measured in the ImageJ software using the mean intensity of the green channel with respect to the RGB color model. For the detection of total ROS in the seedlings, the seedlings were subjected to the same treatment used for leaf labeling. After the washing step, the seedlings were homogenized in PBS solution (1 seedling:5 mL PBS) using a mortar and pestle. The homogenate was centrifuged for 10 min at 4°C and 19,000 rcf. The fluorescence intensity was read using the microplate reader (485-nm excitation and 530-nm emission) and normalized to fresh seedling weight.

#### 2.5.14 Quantification of total silicon in mung seedlings

For the determination of Si in freeze-dried mung seedlings, 0.2 g of homogeneous powder was used for inductively coupled plasma–optical emission spectroscopy (ICP-OES) analysis. The resulting ash at 650°C was subsequently mineralized with 65% HNO_3_ and 30% H_2_O_2_ (8:2) in a Multiwave 3000 microwave digester (Anton Paar, Graz, Austria). After sample digestion, the obtained solution was transferred to a plastic volumetric flask and brought to a final volume of 25 mL with ultrapure water. The blank sample was prepared under the same conditions (Huang et al., 2004).

### 2.6 Statistical analysis

Statistical analysis and Pearson’s correlation were performed using the IBM SPSS 26 software.

## 3 Results

### 3.1 Characterization of RH intermediates and SiNPs

**Figure 1:**
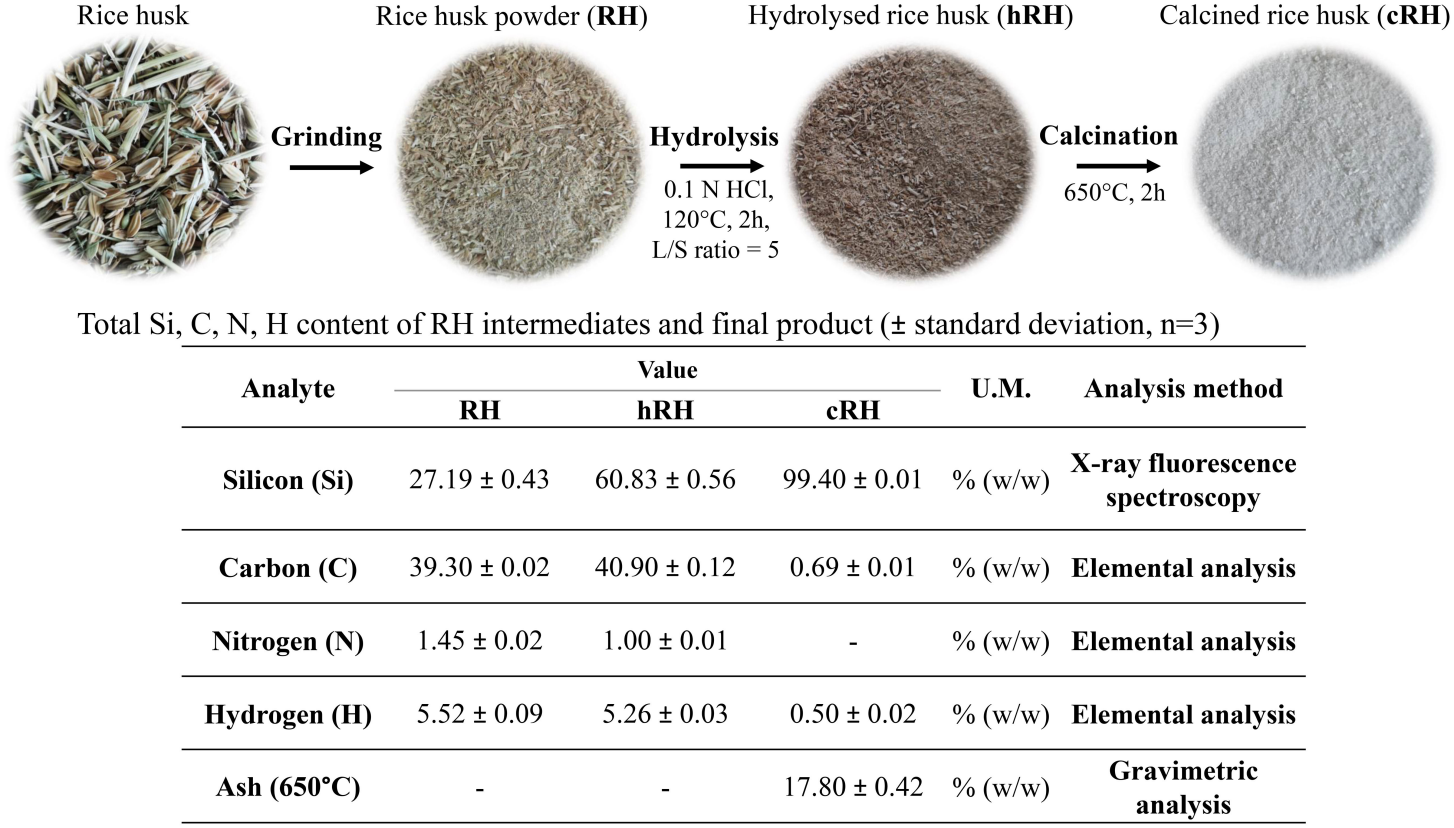
Schematic flow process of silica nanoparticle (SiNP) recovery from rice husk; Si, C, N, and H contents for initial rice husk (RH), hydrolyzed rice husk (hRH), and calcined rice husk (cRH).

The schematic representation and the aspect of the powders obtained at each step of the recovery process of biogenic silica nanoparticles from rice husks can be visualized in **Figure 1**. The dilute acid hydrothermal process led to a black powder due to the hydrocarbonization process of rice husk biomass. The calcination of the hydrolyzed powder for 2 h at 650°C resulted in the thermal carbonization of the lignocellulosic structure, and a white powder was obtained.

XRF analysis (**Figure 1**) revealed the presence of 27% Si in the initial, untreated RH. After the hydrolysis process (hRH), the amount of Si increased to 60%, and it further increased in the product (cRH) after calcination to 99%. Other elements determined by XRF can be found in the **Supplementary Material**. The C content slightly increased upon hydrolysis, from 39.3% in RH to 40.9% in hRH, and it dropped to 0.69% in cRH, as determined by the elemental analysis. The N content decreased from 1.45% in RH to 1% in hRH and was absent in cRH. The H content decreased from 5.52% in RH to 5.26% in hRH and 0.5% in cRH.

**Figure 2:**
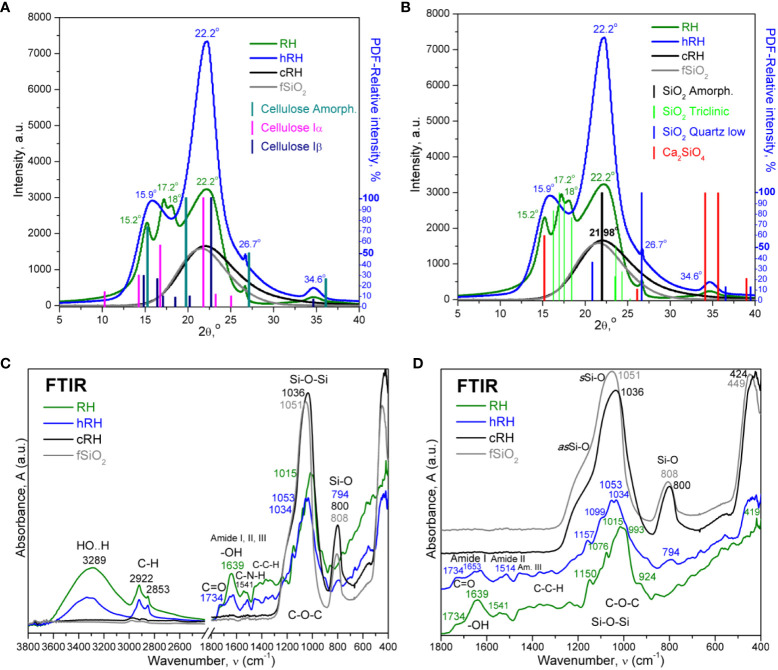
X-ray diffractograms and Fourier transform infrared (FTIR) analysis of rice husk samples: RH, untreated rice husk; hRH, hydrolyzed rice husk; cRH, calcined rice husk; fSiO_2_, commercial fumed silica. **(A)** Treatment step evaluation by X-ray diffraction (XRD) and identification of allomorphs. **(B)** Treatment step evaluation by XRD and identification of silica. The vertical lines represent the relative intensities of the species in the Powder Diffraction File (PDF) from the PDXL software. **(C)** FTIR full spectra. **(D)** FTIR fingerprint region.

The XRD of native RH revealed peaks specific for several allomorphs of silica such as opaline silica (also known as opal, phytoliths in plants, or biogenic amorphous silica), triclinic SiO_2_, calcium silicate (Ca_2_SiO_4_), low-quartz and possible melanophlogite (C_2_H_17_O_5_ * Si_46_O_92_), and diffraction peaks of semi-crystalline cellulose more visible in the hydrolyzed sample. Following the XRD analyses presented in **Figure 2**, it was observed that the triclinic silica (PDF card No. 01-082-1569) coexists structurally with amorphous silica (PDF card No. 00-029-0085), quartz low (PDF card No. 00-005-0490), calcium silicates like Ca_2_SiO_4_ (PDF card No. 00-039-0298), and melanophlogite (PDF card No. 00-025-0007), as well as with cellulose allomorphs Iα (PDF card No. 00-056-1719), Iβ (PDF card No. 00-056-1718), and amorphous cellulose (PDF card No. 00-060-1501). After hydrolysis (hRH), the triclinic silica was decomposed or solubilized together with hemicelluloses, proteins, and other thermally susceptible biocompounds. The main diffraction peaks in hRH were at 15.9° and 22.2°, corresponding mainly to the convoluted signals of calcium silicates, cellulose Iα and Iβ, overlaid on amorphous cellulose and amorphous silica. After calcination (cRH), the cellulose signals disappeared and left behind only the broad diffraction peak at 22° characteristic for amorphous silica (21.98° in PDF card No. 00-029-0085), which evidences the successful preparation of rice husk-derived biogenic SiNPs, similar with recently reported biogenic silica from different types of biomass, including RH (Schneider et al., 2020).

The crystallinity determined by X-ray diffraction (Xc) can be calculated using various methods (French and Cintron, 2013). In this study, the ratio between the areas of the crystalline peaks and the total area (crystalline and amorphous) was used (Park et al., 2010). The crystallinity increased with the reduction of the amount of soft/soluble organic matter, from 66% for the native RH sample to 87% crystallinity for the hydrolyzed sample hRH. The calcined sample cRH was almost completely amorphous (2% Xc) and similar to pyrogenic (fumed) commercial silica. More details about the values of specific diffraction peaks are provided in the **Supplementary Table 2**.

FTIR spectroscopy revealed the structural atom/group vibrations induced by IR radiant energy absorption and rotation vibrations that are characteristic for each bond at specific frequencies/wavenumbers correlated with their energy level (Rana et al., 2018). We employed the ATR-FTIR method to analyze the rice husk as raw material (RH), after acidic hydrolysis (hRH) and after calcination (cRH).

The initial, untreated sample RH presented peaks in both the diagnostic region and the fingerprint region. At high frequencies, between 3,600 cm^−1^ and 3,000 cm^−1^, the stretching vibrations of the covalent hydrogen bonds due to the reduced mass of X−H groups appeared as a broad peak, vibrating from higher to lower frequencies in the approximate order N−H, O−H, ≡C−H, ═C−H, Si−H, Ar−H, and −C−H, based on bond strength *k* and reduced mass *µ*. These vibrations presented decreased intensity in the hRH sample and were almost absent in the calcined sample cRH.

The free −OH groups associated with silanol bonds Si−OH were usually visible at approximately 3,700 cm^−1^, 3,760–3,700 cm^−1^ in our samples (**Figure 2C**; **Supplementary Figure 1**), and the hydrogen-bonded silanol groups vibrated in the region 3,650–3,350 cm^−1^ (Griffith, 1984; Aguiar et al., 2009; Rahman et al., 2009).

In the next region, 3,000–2,800 cm^−1^, the hydrocarbon groups −CH_3_ at 2,960 cm^−1^, −CH_2_ at 2,922 cm^−1^, and −CH at 2,853 cm^−1^ from the lignocellulosic biomass structure were visible. RH and hRH samples had similar absorption peaks, decreasing in intensity with the thermal treatment. These peaks were almost completely reduced in cRH.

The next region, 1,800–1,600 cm^−1^ (**Figure 2D**), included the C═O stretching vibration at 1,734 cm^−1^, which can arise from the carbonyl and uronic ester groups of hemicelluloses (Sheltami et al., 2012; Akinjokun et al., 2021), the −OH vibration in hemicelluloses, cellulose, and polyphenolic lignin at approximately 1,639 cm^−1^, less intense in hRH, and the amide I band (C═O stretching at 1,653 cm^−1^ and C−N at 1,628 cm^−1^), amide II band (N−H bending at 1,541 cm^−1^ coupled with C−N stretching at 1,514 cm^−1^), and amide III band for C−N and N−H deformation, combined with C−C and C−H between 1,460 cm^−1^ and 1,250 cm^−1^. The peaks at approximately 1,650 cm^−1^ included the aromatic skeletal vibrations characteristic of the lignin structure (Hafid et al., 2021). The intensities of the hydrolyzed sample hRH were reduced, which correlated with the loss of soluble compounds by acidic hydrolysis. The calcined sample lacks all absorption bands in this region, being comparable with the commercial fSiO_2_ spectrum.

In the fingerprint region 1,500–400 cm^−1^, the RH and hRH samples presented broad absorption bands characteristic for C−O−C in carbohydrates between 1,200 cm^−1^ and 850 cm^−1^ assigned to various C−O−C stretching and deformation vibrations like glycosidic bond, antisymmetric bridge oxygen, skeletal C−O vibration, and also Si−O bonds. Overlapping in this region were some other bands characteristic of silica bonds such as 1,076 cm^−1^ (asymmetric stretching vibration Si−O−Si) and 924 cm^−1^ assigned to Si−O− (H···H_2_O) bending vibration (Rahman et al., 2009). There were some shifts in wavenumbers in hRH compared to RH, and the main difference between RH and hRH is the absence of the 924 cm^−1^ band in the hydrolyzed sample. For fSiO_2_ and cRH, this region was dominated by the absorption band at 1,040 ± 20 cm^−1^ with a shoulder at approximately 1,180–1,200 cm^−1^ characteristic for the two transverse optical resonant modes of asymmetric stretching of tetrahedral Si−O−Si (Aguiar et al., 2009). The second intense absorption band at approximately 800 cm^−1^ was assigned to the Si−O bending vibration, and at approximately 450 cm^−1^ was assigned to the Si−O−Si in-plane rocking vibrations, and symmetric stretching vibrations of Si−O were assumed (Aguiar et al., 2009; Athinarayanan et al., 2015; Bakar et al., 2016; Tharani and Ananthasubramanian, 2023).

**Figure 3:**
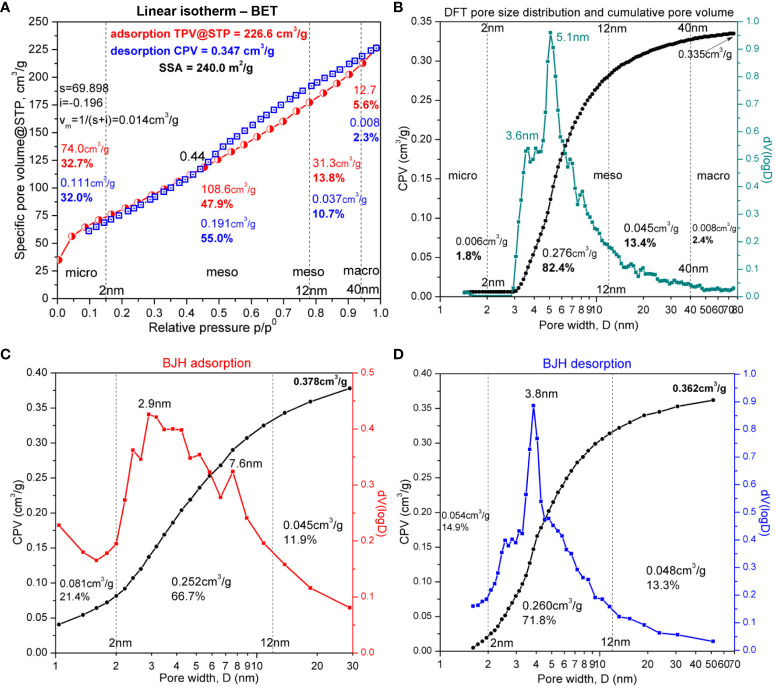
Brunauer–Emmett–Teller (BET) analysis. **(A)** Isotherm of cRH—calcined rice husk. **(B)** Density functional theory (DFT) and pore volume histogram of cRH—calcined rice husk. **(C)** Barrett-Joyner-Halenda (BJH) adsorption model of cRH. **(D)** Barrett-Joyner-Halenda (BJH) desorption model of cRH.

The N_2_ adsorption–desorption isotherm from the BET analysis of the sample cRH (mainly SiNPs), depicted in **Figure 3A**, has a hybrid appearance between type II and type IVa IUPAC adsorption isotherm (Thommes et al., 2015; Fu et al., 2021). The elements of type II isotherm were observed at the extremities of the experimental isotherm at low and high relative pressure p/p^0^. At low relative pressure, the inflexion B-point (or “knee”) can be observed (Brunauer et al., 1938) at approximately 0.045 p/p^0^, which indicates the monolayer adsorption limit, with a total pore volume TPV@STP of 56.2 cm^3^/g corresponding to micropores smaller than 1.4 nm. The total micropore volume for pore diameters smaller than 2 nm (74.0 cm^3^/g TPV@STP) is mentioned in **Figure 3A** in the corresponding region. The volume of monolayer (v_m_) was calculated using the Micropore BET Assistant between the limits 0.004–0.17 p/p^0^ and the formula mentioned in the Quantachrome NovaWin software (displayed in **Figure 3A**), v_m_ = 1/(s + i), where “s” is the slope and “i” is the intercept, resulting in v_m_ = 0.081 cm^3^/g adsorbed N_2_ in micropores as monolayer. At the highest relative pressure, close to p/p^0^ = 1, the adsorption branch was slightly concave, suggesting that the thickness of the adsorbate multilayer tends to increase without limit (Thommes et al., 2015) and without achieving saturation, a fact that points out the presence of macropores, evaluated to be approximately 5.6% v/v from TPV@STP for macropores with diameter D > 40 nm. The characteristic shape of type IVa isotherm is the inflexion B-point (or “knee”) as the upper limit of monolayer adsorption in micropores combined with the hysteresis formed by the adsorption and desorption branches in the upper half of the relative pressure. The type IVa isotherm describes the monolayer adsorption combined with capillary condensation in cylindrical mesopores wider than 4 nm (Thommes et al., 2015).

The physisorption curve has a H3-type hysteresis, characteristic of incompletely filled pores with N_2_ condensate, ranging from 0.44 to 0.99 p/p^0^, with the lower limit being the cavitation-induced p/p^0^ point (Thommes et al., 2015). The hysteresis in the relative pressure range 0.44–0.99 p/p^0^ appears due to capillary condensation in mesopores. The adsorption branch of the hysteresis corresponds to delayed condensation in mesopores due to metastable adsorption films associated with nucleation barriers, while the desorption branch corresponds to the equilibrium of liquid–vapor transitions (Thommes and Cychosz, 2014). Therefore, at a value close to 0.95 for p/p^0^ on the desorption branch, the volume of liquid N_2_ can be calculated at equilibrium with the vapor phase by assuming that the mesopores are filled with N_2_ in the bulk liquid state (Thommes and Cychosz, 2014). The volume of liquid N_2_ calculated at 0.97 p/p^0^ on the desorption branch, using the tag volume at the corresponding experimental point, was 0.347 cm^3^/g for the BET method, close to 0.36 cm^3^/g obtained in a similar study (Schneider et al., 2020), and 0.335 cm^3^/g for the NL-DFT method presented in **Figure 3B**. The values were computationally assigned by the software to the total pore volume for pores with diameters less than 67.24 nm.

In **Figure 3A**, it can be observed that cRH had a specific total pore volume at standard temperature and pressure (STP) of 226 cm^3^/g and a specific surface area of 240 m^2^/g, comparable with a 297 m^2^/g value for similar biogenic SiNPs obtained from RH by hydrolysis with citric acid and gradual calcination from 310°C to 599°C (Schneider et al., 2020).

SiNPs obtained by hydrolysis and calcination showed a relatively homogeneous distribution of mesopores between 2.5 nm and 20 nm, almost Gaussian centered at approximately 5 nm, with the majority of pores smaller than 10 nm (**Figure 3**).

All simulation methods used resulted in the identification of micropores (<2 nm), mesopores (2–40 nm), and some macropores (>40 nm), with the exception of the DR method, which resulted in 0% macropores. Although the general limit between mesopores and macropores is approximately 50 nm (Thommes et al., 2015), the value of 40 nm was chosen mainly to compare BJH with the other methods. This was because BJH gave only a few points over 40 nm in our case, one point at 94.1 nm for the adsorption branch (**Figure 3C**) and two points at 51.4 nm and 109.4 nm for the desorption branch (**Figure 3D**). The data for micropores and mesopores are presented in **Figure 3** and **Supplementary Table 3**. The volume of micropores determined by the fit of the linear region with the BET method (0.111 cm^3^/g) was similar to the value obtained by the DR method (0.115 cm^3^/g). The next closest value (0.081 cm^3^/g) was obtained by applying the BJH method on the adsorption curve (**Figure 3C**) and was the same as the volume of monolayer, v_m,_ reported above. NL-DFT significantly underestimated the volume of micropores (**Figure 3B**). The specific surface area of micropores was 69.23 cm^3^/g, as determined by the t-plot. The volume of mesopores was similarly estimated by the BJH and NL-DFT function (approx. 0.3 cm^3^/g) but was significantly underestimated by the DR method (0.103 cm^3^/g). The mesopore volume determined from the BET method (0.191 cm^3^/g) was the average between the value from BJH/NL-DFT and the DR method. The following values were determined for the cumulative macropore volume with each method: MV_BET_ = 0.008 cm^3^/g, MV_DFT_ = 0.008 cm^3^/g, MV_BJHa_ = 0.023 cm^3^/g, and MV_BJHd_ = 0.004 cm^3^/g. All in all, the simulation that seems to reflect most accurately all pore types, in this case, is the BJH function applied on the adsorption isotherm.

SEM-EDX analysis revealed that following the calcination, there was a reduction in the amount of organic matter and reduced particle size from 100–200 μm in the untreated/initial RH to <50 μm after the calcination process (**Supplementary Figure 2**, **Supplementary Table 4**).

**Figure 4:**
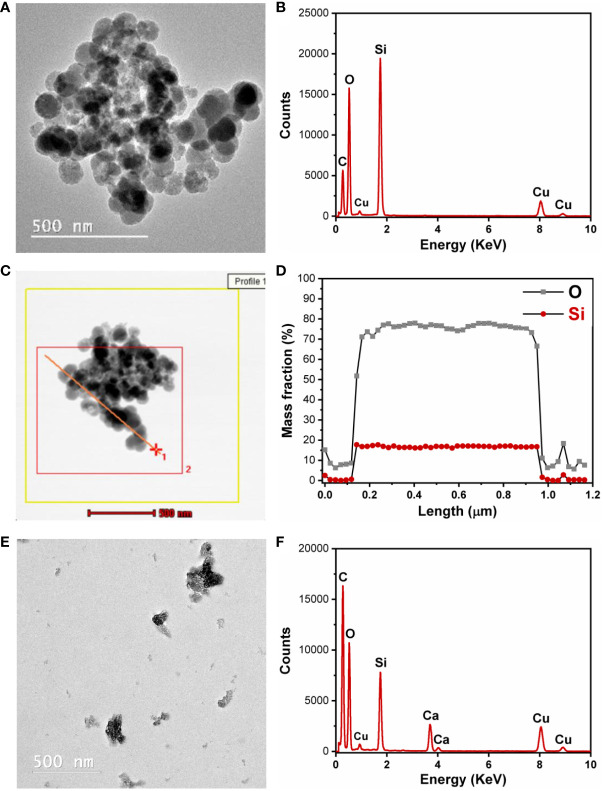
Transmission electron microscopy–energy dispersive X-ray (TEM-EDX) analysis. **(A)** Silica nanoparticles (SiNPs) from rice husk. **(B)** EDX spectrum of SiNPs. **(C)** Scanning transmission electron microscopy (STEM)–EDX analysis for achieving a linear profile composed of 50 spectra. **(D)** The composition profile obtained after STEM-EDX analysis. **(E)** SiNPs from rice husk in alginate solution after probe sonication. **(F)** EDX spectrum of SiNPs in alginate solution after probe sonication.

TEM analysis provides qualitative information about the morphology of nanoparticles such as shape and size. The STEM module coupled with the EDX detector can give information about the variation of the elements that are present in the sample in the analyzed area. Quasi-spherical nanoparticles of approximately 50 nm were synthetized, but they were mostly in an aggregated state (**Figure 4A**). The EDX spectrum is presented in **Figure 4B**. The analysis in STEM/EDX mode allowed us to achieve a linear profile composed of 50 spectra, which can be summed up to give a global spectrum of the analyzed area (**Figure 4C**). The main components from rice husk SiNPs were silicon and oxygen, and there were no major variations based on the linear profile achieved by STEM/EDX analysis (**Figure 4D**). Because the SiNPs from RH were mostly agglomerated, a stabilizing process and agent were necessary, and the SiNPs were dispersed in alginate. The presence of alginate in the spaces between the SiNPs partially prevents the agglomeration of SiNPs (**Figure 4E**). The EDX spectra of SiNPs embedded in alginate are presented in **Figure 4F**.

The mean diameter of SiNPs determined by DLS using the SBL function provided by the software is shown in **Table 1**. This function and the Pade–Laplace function gave the best fitting. The residuals from the Cumulants function were higher than the threshold, but this model provided the value for the polydispersity index of 0.2785. The values of the mean diameter resulting from the Pade–Laplace were 109.71 nm (95% volume) and 436.26 nm (5% volume). The full data of the DLS analysis are provided in the **Supplementary Material**.

**Table 1:** Dynamic light scattering (DLS) analysis of silica nanoparticles (SiNPs) from rice husk (RH).

Mean diameter (nm)	Intensity (%)	Volume (%)	Number (%)
86.21			100
130.72		100	
552.79	100		

**Figure 5:**
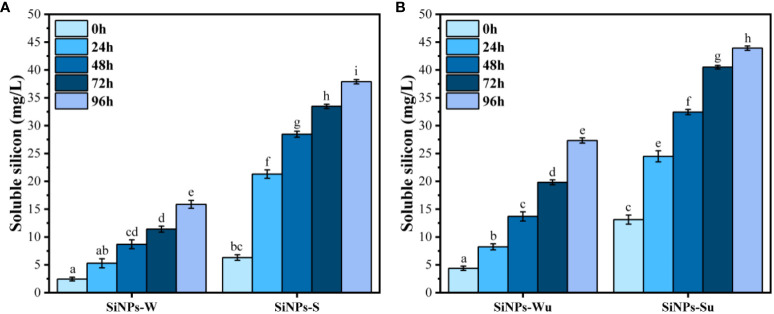
Soluble silicon release from silica nanoparticles (SiNPs): SiNPs-W, SiNPs in water; SiNPs-S, SiNPs in 50 mM NaCl; SiNPs-Wu, ultrasonicated SiNPs in water; SiNPs-Su, ultrasonicated SiNPs in 50 mM NaCl. **(A)** Soluble silicon from SiNPs. **(B)** Soluble silicon from ultrasonicated SiNPs ( ± error bars, α = 0.05, n = 3; different letters indicate statistically significant differences between samples).

**Figure 5** shows the release of soluble Si from SiNPs in time, in the absence and presence of 50 mM NaCl (the salt concentration used later for inducing abiotic stress on mung beans). A constant release of soluble silicon was observed in time, with the concentration increasing day to day during the 4 days of testing. The release was slightly higher for the ultrasonicated samples (**Figure 5B**) compared to the non-ultrasonicated samples (**Figure 5A**) and significantly higher in the presence of 50 mM NaCl compared with pure water for both ultrasonicated and non-ultrasonicated samples.

### 3.2 Optimization and characterization of the seed coating

The traction tests yielded Young’s modulus and the UTS for each film (**Supplementary Table 1**). Young’s modulus was best described by a modified quadratic Scheffe model, which, apart from the linear factors, also considered interaction effects A–B and A–C. The large max-to-min ratio suggested that a data transformation is required. The natural log function was used. This model fitted the data considering the p-values for the terms expressed in **Supplementary Table 5**. This was also confirmed by the R^2^ and adjusted R^2^ values (0.9875 and 0.9707, respectively). A low p-value corresponding to the lack of fit test suggests that the design could be augmented to accurately represent the data.

UTS was modeled by a linear Scheffe model with R^2^ and adjusted R^2^ values of 0.8671 and 0.8139, respectively. In this case, no transformation was required. The ANOVA for this response variable is shown in **Supplementary Table 6**, where a p-value for the model is explained by the linear term. The lack-of-fit test has a p-value of 0.2814, in this case, pointing to a model that fits the data.

**Figure 6:**
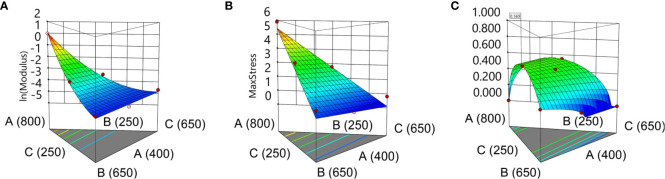
Design-Expert. **(A)** $\ln(YM[MPa])=f(X_{1},\;X_{2},\;X_{3})=1.04\cdot X_{1}-3.97\cdot X_{2}-3.86\cdot X_{3}-3.66\cdot X_{1}\cdot X_{2}-4.84\cdot X_{1}\cdot X_{3}.$ **(B)** $UTS[MPa]=f(X_{1},\;X_{2},\;X_{3})=5.43\cdot X_{1}+0.7301\cdot X_{2}+0.1088\cdot X_{3}$ **(C)** Desirability function with optimum point at [Min (YM), Max (UTS)].

Considering that the statistical models for the two response variables are adequate, a desirability function can be used to determine the optimum mixture of ingredients. This function, which is automatically generated through the Design-Expert 11 software, considers that the optimum point should be found in the initial space defined by the initial simplex centroid design, minimizing Young’s modulus while maximizing the ultimate tensile strength. The two models have equal weights with respect to the desirability function. Intuitively, the optimum model (maximum desirability) should give the most elastic film, which also presents relatively high UTS. This corresponds to the practical observations that suggest that the seed coating process requires an elastic coating formula that also withstands moderately high UTS values (seed-to-seed impact or seed-to-wall impact). The optimum mixture with respect to the previously defined desirability was found by mixing 678 mg of sodium alginate, 250 mg of glycerol, and 372 mg of sorbitol, having an estimated value of 0.111 MPa for Young’s modulus and 3.245 MPa for UTS. The two statistical models corresponding to each response variable are shown as a surface in **Figure 6A** for ln(YM) and **Figure 6B** for UTS. The desirability function on the design space is shown in **Figure 6C**. The corresponding equations YM and UTS are given under the 3D surface plots as a function of the composition for the three ingredients expressed in terms of L_Pseudo Components.

**Figure 7:**
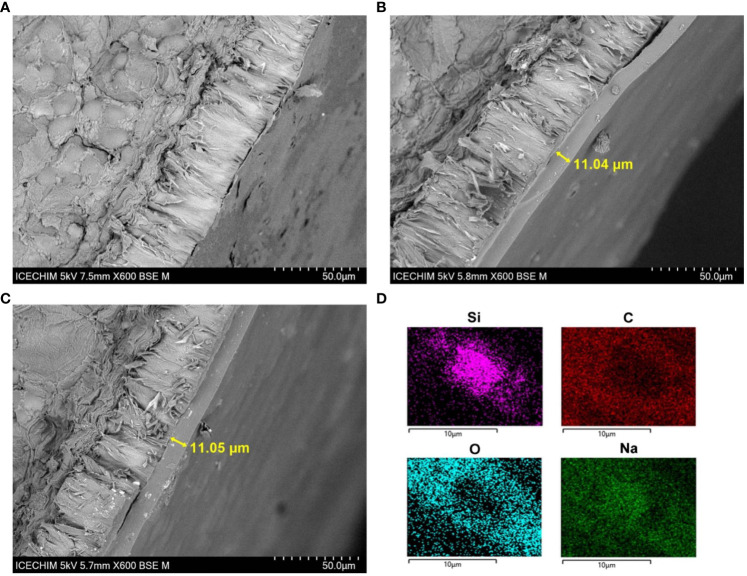
Scanning electron microscopy (SEM) micrographs of cross-sectioned mung bean seeds. **(A)** Uncoated. **(B)** Alginate–glycerol–sorbitol (AGS) coating. **(C)** AGS + silica nanoparticle (SiNP) coating. **(D)** Elemental composition of AGS + SiNP coating.

SEM micrographs of cross-sectioned mung bean seeds indicate that the seed coating has a uniform distribution (**Figures 7B, C**) with an average size of 11 µm. Using the EDX detector, we obtained the composition of the seed coating with embedded SiNPs, and the main detected elements were Si, C, O, and Na. Some large phytoliths could be clearly distinguished in the film (**Figure 7D**). **Figure 7A** shows the cross-section of uncoated mung bean seed.

Water activity is a measure of the amount of unbound/free water in the sample of interest. We wanted to see if the coating process had an impact on the water activity. The results presented in **Supplementary Table 7** indicate that there was no change in the free water content following the application of different treatments.

### 3.3 Seedling growth and development

**Figure 8:**
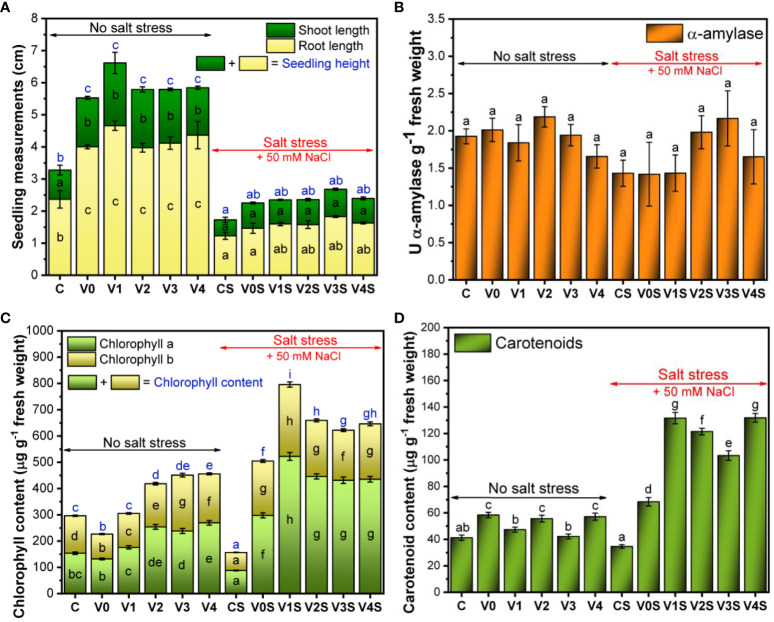
Plant growth and development: C, control, no salt stress (uncoated mung bean seeds); V0, alginate–glycerol–sorbitol (AGS) solution seed coating, no salt stress; V1, AGS solution + 1% silica nanoparticle (SiNP) seed coating, no salt stress; V2, AGS solution + 2% SiNP seed coating, no salt stress; V3, AGS solution + 3% SiNP seed coating, no salt stress; V4, AGS solution + 4% SiNP seed coating, no salt stress; CS, control, salt stress (uncoated mung bean seeds); V0S, alginate solution seed coating, salt stress; V1S, alginate solution + 1% SiNP seed coating, salt stress; V2S, alginate solution + 2% SiNP seed coating, salt stress; V3S, alginate solution + 3% SiNP seed coating, salt stress; V4S, alginate solution + 4% SiNP seed coating, salt stress. **(A)** Plant measurements. **(B)** α-Amylase activity. **(C)** Chlorophyll content. **(D)** Carotenoid content ( ± error bars, α = 0.05, n = 3; different letters indicate statistically significant differences between samples).

The root length of the mung bean seedlings showed a statistically significant reduction in the presence of 50 mM NaCl as compared to the absence of salt stress. There was no statistically significant change in the shoot length induced by salt in comparison to normal conditions. In the absence of salt, coating the seeds with the AGS ± SiNP film induced a statistically significant increase of both root and stem length compared to the uncoated seeds (C), with no significant changes between the experimental variants V0–V4. In the presence of 50 mM salt, this stimulation effect was only marginally significant and only in the case of the roots for the AGS + SiNP-coated seeds, i.e., the V1–V4 experimental variants compared with control, without significant differences between the SiNP doses (**Figure 8A**). The images of selected seedlings can be found in **Supplementary Figures 3, 4**.

Neither salt nor the film had a statistically significant effect on the α-amylase activity (**Figure 8B**). There was a tendency toward lower α-amylase activity under salt stress (1.43 U α-amylase g^−1^ fresh weight) compared to no salt (1.92 U α-amylase g^−1^ fresh weight), which seemed to be overcome by the treatment with the 2% and 3% SiNP AGS films (1.98 U α-amylase g^−1^ fresh weight and 2.16 U α-amylase g^−1^ fresh weight, respectively).

Chlorophyll *a* was present in higher concentration in comparison with chlorophyll *b*, but the pattern of increase/decrease as a function of treatment was similar (**Figure 8C**). In the absence of salt, the total chlorophyll content of the uncoated mung bean seedling (C) was 296.1 ± 7.1 µg/g fresh weight (FW) with a significant decrease to 226.5 ± 5.6 µg/g FW in the case of AGS treatment (V0). Following the AGS + SiNP treatment, there was a significant increase in chlorophyll content as the dose of SiNPs increased, reaching 455.5 ± 12.3 µg/g FW in the case of AGS + 4% SiNP coating (V4). The differences became marginally significant between V2 and V3, and V3 and V4 variants. In the presence of 50 mM NaCl, the chlorophyll content of the uncoated control (CS) was significantly lower (156.7 ± 2.5 µg/g FW) in comparison with the content obtained in the absence of salt. The AGS treatment (V0S) induced a significant increase in chlorophyll content of up to 504.6 ± 14.6 µg/g FW, followed by a significant further increase in the presence of AGS + SiNP treatments, with the maximum content of 795.6 ± 24.3 µg/g FW reached in the case of AGS + 1% SiNP (V1S) treatment. The chlorophyll content decreased in the case of higher SiNP concentration variants but remained significantly above CS. In the presence of salt, chlorophyll *b* was more affected than chlorophyll *a* (**Figure 8C**).

The carotenoid content of the uncoated mung bean seedlings (C) was 41.2 ± 1.9 µg/g FW. The AGS treatment induced an increase in the carotenoid content up to 58.3 ± 2.0 µg/g FW, which remained relatively constant following the treatment with SiNPs, with a slight apparent hormesis. In the presence of salt stress, there was a decrease in the carotenoid content of the uncoated mung bean seedlings (34.6 ± 1.3 µg/g FW for CS). Following the AGS treatment, the carotenoids increased up to 68.4 ± 3.1 µg/g FW, with a further increase up to 131.6 ± 4.2 µg/g FW in the case of AGS + 1% SiNP (V1S) treatment, which remained constant for V4S (**Figure 8D**). The intermediary 2% and 3% SiNPs stimulated less the carotenoid content, suggesting a hormetic behavior.

### 3.4 Metabolic and biochemical activities of the developed seedlings

**Figure 9:**
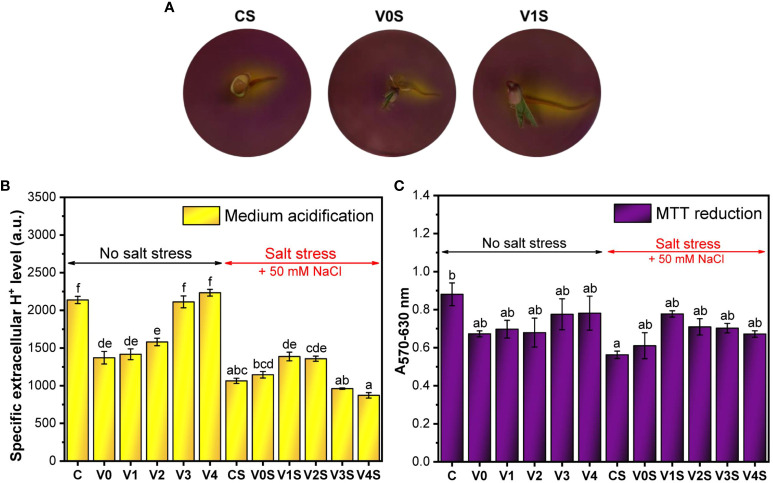
Metabolic activity of mung bean seedlings: C, control, no salt stress (uncoated mung bean seeds); V0, alginate–glycerol–sorbitol (AGS) solution seed coating, no salt stress; V1, AGS solution + 1% silica nanoparticle (SiNP) seed coating, no salt stress; V2, AGS solution + 2% SiNP seed coating, no salt stress; V3, AGS solution + 3% SiNP seed coating, no salt stress; V4, AGS solution + 4% SiNP seed coating, no salt stress; CS, control, salt stress (uncoated mung bean seeds); V0S, alginate solution seed coating, salt stress; V1S, alginate solution + 1% SiNP seed coating, salt stress; V2S, alginate solution + 2% SiNP seed coating, salt stress; V3S, alginate solution + 3% SiNP seed coating, salt stress; V4S, alginate solution + 4% SiNP seed coating, salt stress. **(A)** Agar medium acidification. **(B)** Specific extracellular H^+^ level. **(C)** MTT reduction ( ± error bars, α = 0.05, n = 3; different letters indicate statistically significant differences between samples).

**Figure 9B** shows that in the absence of salt stress, the germinated seeds coated with the AGS film without SiNPs (V0) had a significantly lower seH^+^ level compared to the uncoated germinated seeds (C). There was an increase in seH^+^ following coating with the AGS film embedding SiNPs compared with simple AGS, an increase that was SiNP concentration-dependent. The seH^+^ at 3% and 4% SiNP doses (V3 and V4, respectively) reached the value obtained in the absence of coating (C) (**Figure 9B**). Under salt stress, seH^+^ decreased significantly, below V0. The seedlings from the seeds with the AGS films, especially embedding SiNPs at 1% and 2%, partially recovered the seH^+^, but the latter could not reach the value of the uncoated seeds in the absence of salt (**Figures 9A, B**). The total released H^+^ (eH^+^) was not significantly affected by the AGS film but was significantly affected by salt stress (**Supplementary Figures 5, 6**). SiNPs significantly increased the total H^+^ in the absence of salt only at 2% and 3% SiNP concentration. The effect in the presence of salt was not statistically significant (**Supplementary Figure 6**).

A similar pattern to seH^+^ is observed for the MTT reduction, but the effects of salt or coating were not as significant as in the case of seH^+^ (**Figure 9C**).

**Figure 10:**
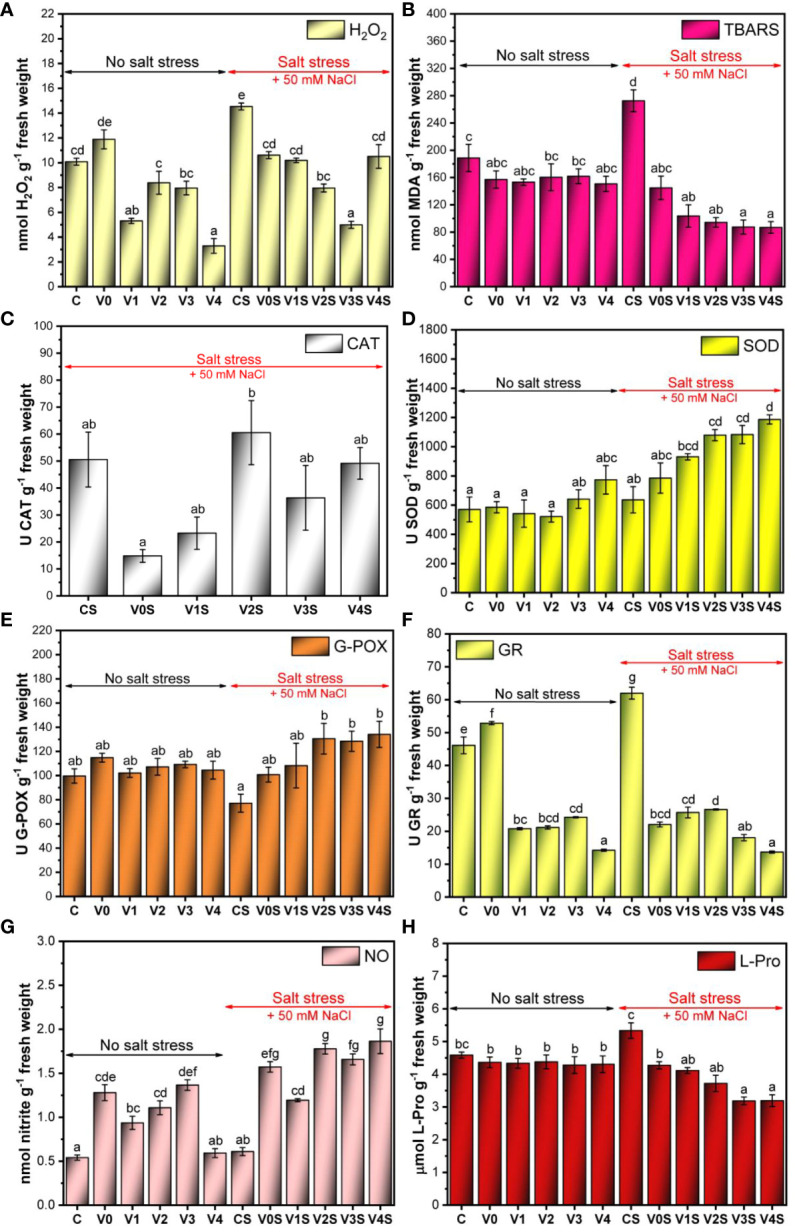
Reactive oxygen species (ROS) metabolism in mung bean seedlings: C, control, no salt stress (uncoated mung bean seeds); V0, alginate–glycerol–sorbitol (AGS) solution seed coating, no salt stress; V1, AGS solution + 1% silica nanoparticle (SiNP) seed coating, no salt stress; V2, AGS solution + 2% SiNP seed coating, no salt stress; V3, AGS solution + 3% SiNP seed coating, no salt stress; V4, AGS solution + 4% SiNP seed coating, no salt stress; CS, control, salt stress (uncoated mung bean seeds); V0S, alginate solution seed coating, salt stress; V1S, alginate solution + 1% SiNP seed coating, salt stress; V2S, alginate solution + 2% SiNP seed coating, salt stress; V3S, alginate solution + 3% SiNP seed coating, salt stress; V4S, alginate solution + 4% SiNP seed coating, salt stress. **(A)** H_2_O_2_ content. **(B)** Thiobarbituric acid reactive substance (TBARS) content. **(C)** Catalase (CAT) activity. **(D)** Superoxide dismutase (SOD) activity. **(E)** Guaiacol peroxidase (G-POX) activity. **(F)** Glutathione reductase (GR) activity. **(G)** Nitric oxide (NO) content. **(H)** l-Proline (L-Pro) content ( ± error bars, α = 0.05, n = 3; different letters indicate statistically significant differences between samples).

There was a marginally significant increase in the H_2_O_2_ content in the absence of salt stress for the AGS treatment (V0), followed by a significant decrease for V1 (AGS + 1% SiNPs) and V4 (AGS + 4% SiNPs) compared to C. In the case of V2 and V3, the H_2_O_2_ content was situated just slightly below the control value, remaining relatively constant between the two variants (8 nmol H_2_O_2_ g^−1^ FW). In the presence of 50 mM NaCl, the uncoated mung bean seedlings (CS) had the highest H_2_O_2_ content (14.5 nmol H_2_O_2_ g^−1^ FW). The AGS coating (V0S) as well as the AGS + SiNP treatments induced a significant decrease in the H_2_O_2_ content compared to CS. The lowest H_2_O_2_ concentration (5 nmol H_2_O_2_ g^−1^ FW) in the presence of salt was recorded for the V3S variant (AGS + 3% SiNPs). The highest dose of SiNPs, i.e., AGS + 4% SiNPs (V4S) increased the H_2_O_2_ content up to the level of the V0S and C variant (**Figure 10A**). The quantitative determinations of H_2_O_2_ in mung bean seedlings correlated with the images acquired following the DAB leaf staining for H_2_O_2_ detection (**Supplementary Figure 7**).

In the absence of salt stress, the level of lipid peroxides in the cell was kept relatively constant, with a marginally significant decrease in the case of the AGS film coating (V0–V4) compared with control (C) and no apparent influence of SiNPs (**Figure 10B**). Salt stress induced a significant increase in lipid peroxides from 188 nmol (C) to 272 nmol MDA g^−1^ FW in the uncoated control (CS). Simple AGS coating (V0S) induced a marked decrease in MDA compared to CS, down to 145 nmol MDA g^−1^ FW, and SiNPs further decreased the MDA in a SiNP dose-dependent manner (103 nmol MDA g^−1^ FW in V1S, 94 nmol MDA g^−1^ FW in V2S, 87 nmol MDA g^−1^ FW in V3S, and 86 nmol MDA g^−1^ FW in V4S).

We investigated the activity of four main enzymes involved in ROS scavenging, CAT, SOD, G-POX, and GR, as can be seen in **Figure 10**.

The untreated control exhibited a CAT activity of 50.5 U CAT g^−1^ FW in the presence of salt stress (CS). Following treatment with AGS (V0S), the CAT activity decreased significantly to 14 U CAT g^−1^ FW (**Figure 10C**). The presence of SiNPs recovered the CAT activity to values in the range of the control (CS), with a maximum of 2% SiNPs. There was no catalase activity in the absence of salt stress; therefore, the experimental data set was not included in the graph.

The SOD activity was relatively constant in the absence of salt (521-570 U SOD g^−1^ FW), with a slight increase at 3% SiNPs (V3, 641 U SOD g^−1^ FW) and 4% SiNPs (V4, 772 U SOD g^−1^ FW). Under salt stress, the uncoated control CS showed an activity of 636 U SOD g^−1^ FW. The AGS seed coating treatment (V0S) induced an increase in the SOD activity to 784 U SOD g^−1^ FW, which was marginally significant compared to the uncoated seeds (**Figure 10D**). Further increases appeared in the SiNP AGS seed coating treatments in a SiNP dose-dependent manner (935 U SOD g^−1^ FW in V1S, 1080 U SOD g^−1^ FW in V2S and V3S, and 1186 U SOD g^−1^ FW in V4S).

In the absence of salt stress, G-POX showed an activity between 100 U and 115 U g^−1^ FW, and the differences between coated and uncoated seeds were not statistically significant. A marked decrease in G-POX activity was noted in the untreated control (CS) under salt stress (77 U G-POX g^−1^ FW). Following AGS (V0S) and 1% SiNP–alginate (V1S) seed coating treatments, the G-POX activity increased to values in the range of those found in the absence of salt stress (100 U G-POX g^−1^ FW in V0S and 108 U G-POX g^−1^ FW in V1S). Further increase in G-POX activity was obtained for 2% SiNPs, 3% SiNPs, and 4% SiNP AGS seed coating treatments compared with 1% SiNPs, with an apparent activity saturation after 2% SiNPs (**Figure 10E**).

In the absence of salt stress, GR activity slightly increased after the AGS treatment (V0) in comparison with uncoated mung bean seedlings (C). All the AGS + SiNP treatments decreased the GR activity from 46 U/g FW in C to 24 U/g FW and below, the most significant decrease being recorded in the case of AGS + 4% SiNP treatment (V4). In the presence of 50 mM NaCl, the uncoated mung bean seedlings (CS) had the highest GR activity, more than 60 U/g FW (**Figure 10F**). A significant decrease compared to CS was observed following the AGS treatment (V0), with a subsequent significant decrease following the treatments with the highest concentrations of SiNPs (V3S and V4S).

The NO content increased in the case of V0 in the absence of salt stress in comparison with the uncoated mung seedlings (C), from 0.5 nmol/g FW to 1.25 nmol/g FW. The treatment with the lowest concentration of SiNPs (AGS + 1% SiNPs, V1) induced a significant decrease in the NO content compared to V0. By increasing the concentration of SiNPs up to 3%, the amount of NO increased in a SiNP dose-dependent manner, reaching the value noticed in the case of AGS treatment (V0). At the highest concentration of SiNPs (AGS + 4% SiNPs, V4) a significant decrease down to the level of control (C) was observed. In the presence of salt stress, the NO content in the case of the uncoated mung seedlings (CS) was only slightly higher compared to that in the same control in the absence of salt. The AGS coating ± SiNPs induced a significant increase in the NO content, but in the case of V1S, the NO concentration was lower in comparison with that of the other seed coating treatments (**Figure 10G**).

In the absence of salt stress, there were no significant changes in the l-proline content as can be seen in **Figure 10H** (an average of 4.3 µmol l-Pro g^−1^ FW in all cases), except V0, which slightly decreased it compared with the control, with no further changes for V1-V4. Under salt stress, the l-proline content increased in the uncoated control (5.3 µmol l-Pro g^−1^ FW in CS) and decreased for mung beans subjected to simple AGS seed coating (4.3 µmol l-Pro g^−1^ FW in V0S). Seed coatings based on AGS and SiNPs led to further SiNP dose-dependent decrease in the proline content (4.11 µmol g^−1^ FW in V1S, 3.7 µmol g^−1^ FW in V2S, 3.18 µmol g^−1^ FW in V3S, and 3.19 µmol g^−1^ FW in V4S).

**Figure 11:**
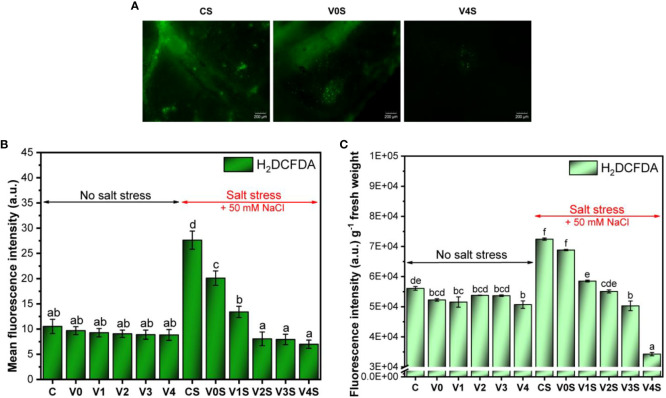
Reactive oxygen species (ROS) accumulation in mung bean seedlings: C, control, no salt stress (uncoated mung bean seeds); V0, alginate–glycerol–sorbitol (AGS) solution seed coating, no salt stress; V1, AGS solution + 1% silica nanoparticle (SiNP) seed coating, no salt stress; V2, AGS solution + 2% SiNP seed coating, no salt stress; V3, AGS solution + 3% SiNP seed coating, no salt stress; V4, AGS solution + 4% SiNP seed coating, no salt stress; CS, control, salt stress (uncoated mung bean seeds); V0S, alginate solution seed coating, salt stress; V1S, alginate solution + 1% SiNP seed coating, salt stress; V2S, alginate solution + 2% SiNP seed coating, salt stress; V3S, alginate solution + 3% SiNP seed coating, salt stress; V4S, alginate solution + 4% SiNP seed coating, salt stress. **(A)** Fluorescence microscopy observations of H_2_DCFDA-mediated fluorescence in seedling leaves. **(B)** Mean fluorescence intensity (leaf ROS). **(C)** Total seedling ROS detection ( ± error bars, α = 0.05, n = 3; different letters indicate statistically significant differences between samples).

Following the determination of H_2_DCFDA-mediated fluorescence to detect total ROS in mung bean leaf and total ROS in seedlings, our results indicated a basal level of ROS in the control, the leaf ROS unaffected and the total ROS slightly reduced by coating, in the absence of salt stress (**Figures 11A–C**, **Supplementary Figure 8**). A significant increase in ROS level was induced by salt stress in the uncoated mung bean leaves (CS), approximately three times more in leaves and 50% more ROS in seedlings than for C without salt (**Figures 11B, C**). The simple AGS seed coating (V0S) produced a significant decrease in leaf ROS and a slight decrease in the seedling ROS. The seed coatings based on AGS and SiNPs further induced a SiNP dose-dependent significant decrease in ROS level that became lower than the one in the absence of salt (**Figures 11A-C**).

### 3.5 Total silicon content in the mung seedlings

The total silicon content was in general higher in the mung seedlings treated with SiNPs compared with the mung seedlings in the control or treated only with the AGS film (**Table 2**). The content was not linearly correlated with the SiNP concentration tested, suggesting that the Si absorption in the seedlings is not merely a passive process but involves some active mechanisms. A possible influence of NaCl on the Si absorption in the seedlings was not clearly evident.

**Table 2:** ICP-OES analysis of total silicon content from freeze-dried mung seedlings.

Analyte	Value (mg/kg dry weight) ± standard deviation (SD)											
	C	V0	V1	V2	V3	V4	CS	V0S	V1S	V2S	V3S	V4S
**Si (λ = 251.611 nm)**	33.3 ± 2.7 a*	54.2 ± 7.0 b	99.8 ± 3.4 d	169.1 ± 4.0 ef	158.0 ± 7.4 e	190.0 ± 5.5 g	44.7 ± 2.3 ab	59.6 ± 2.6 b	78.3 ± 6.3 c	107.9 ± 1.1 d	175 ± 6.8 fg	162 ± 5.8 ef

ICP-OES, inductively coupled plasma–optical emission spectroscopy.

* Different letters indicate statistically significant differences between samples.

## 4 Discussions

### 4.1 Mung seeds can be efficiently coated with alginate-based film embedding biogenic SiNPs from rice husks

We report the first study, to the best of our knowledge, of using biogenic SiNPs as a plant biostimulant ingredient in an optimized seed coating film. The SiNPs obtained from RH in our study are similar to previously reported SiNPs obtained by similar processes (Athinarayanan et al., 2015; Bakar et al., 2016), but we provide here a more in-depth characterization of the SiNP properties important for the biological activity and of the process that generates the nanoparticles. By acidic hydrolysis, the proteins, the soluble fibers, the hemicellulose, and part of the amorphous cellulose of RH are denatured. By further calcination in air at 650°C, the broad peak between 3,600 cm^−1^ and 3,000 cm^−1^ and the peaks in the region 1,800–1,600 cm^−1^ of FTIR spectra disappeared because the ligno-cellulosic structure is thermally decomposed, and the remaining ash is represented by SiNPs, with silanol group signals at approximately 3,730 cm^−1^. The obtained SiO_2_ had a purity of 99% as determined by XRF. The dilute acid hydrolysis removes not only part of organic matter but also inorganic impurities that have been proposed to favor the formation of crystalline forms of SiNPs during calcination (Tharani and Ananthasubramanian, 2023). Dilute acid pretreatment combined with calcination at temperatures below 900°C leads to the formation of only amorphous SiNPs (Bakar et al., 2016).

Biochemical processes that occur at the interface between substrate and liquid environment are strongly dependent on the interface area. Textural properties like specific surface area, porosity, pore size, and particle size are relevant for the bioactivity of biomaterials such as the biogenic SiNPs from RH in our case. The acid pretreatment was previously shown to induce higher surface area and higher porous structure of SiNPs than without acid leaching (Bakar et al., 2016). Similar or higher surface area, total pore volume, and pore diameters of SiNPs from RH to those reported by other studies (Bakar et al., 2016; Tharani and Ananthasubramanian, 2023) were obtained in our case. The specific surface area of 240 m^2^/g for our mesoporous biogenic SiNPs assures a highly active interface for the release of orthosilicic acid Si(OH)_4_ and interaction with the bioactive compounds from the seed coating solution. The release of Si(OH)_2_ is demonstrated to occur steadily during the 4 days of our experiment (**Figure 5**), and this is in agreement with previous studies, which claim that SiNPs are in equilibrium with the soluble forms of Si in aqueous solutions (Spitzmüller et al., 2023).

It is possible that the micropores and small mesopores (<12 nm) physically adsorb the glycerol and sorbitol molecules or establish hydrogen bonds with their functional groups. The adsorption in micropores and mesopores might facilitate the slow release of these compounds to the seeds. The macromolecules of alginate could interact with the macropores and large mesopores (12–40 nm), creating a SiNP–alginate bioactive nanocomposite coating. The presence of macropores is indicated by the lack of saturation at the maximum relative pressure in the adsorption isotherm (**Figure 3A**). The hysteresis of type H3 present at desorption could indicate the formation of slit-like pores (Tharani and Ananthasubramanian, 2023).

Particle size distribution strongly influences the physicochemical and optical properties of SiNPs, as well as their biological activity. Comparable results were reported for synthetic SiNPs in the particle range 7–400 nm (Rahman et al., 2009), with our SiNPs being placed in the range of 50–150 nm mean particle size, with the formation of some aggregates as well, and with corresponding pore volume and surface area. The temperature of calcination was shown to influence the size of SiNPs from biomass; i.e., the higher the temperature, the smaller the size (Athinarayanan et al., 2015). Previous studies reported SiNPs from RH with 10–30-nm diameters depending on the calcination temperature. Other studies reported silica aggregates as nanoscale spheres with 100- and 150-nm diameters, with the process involving also the alkaline treatment of the ash (Tharani and Ananthasubramanian, 2023). The aggregation in clusters is probably induced by calcination due to the removal of organic material. In our case, the dispersion in alginate reduced the aggregation.

Alginate-based coating of seeds has been recently reported in the scientific literature, but it was in general achieved by dip or spray method followed by drying in a drying oven, or in some cases, the coating method is not mentioned (De Castro et al., 2020; Behboud et al., 2021; Skrzypczak et al., 2021). This method does not guarantee homogeneous coating of the seed, and the drying performed on solid surfaces affects the coating on the seed surface in contact with the solid surface. We have recently optimized an alginate-based film coating on mung beans, but with a different composition than in the present study and without SiNPs, using the Wurster process (Trica et al., 2023). We showed that the coating did not impact the seed germination, but we did not go more in-depth in the seedling characterization. There are no other available studies that included pre-optimization of the film properties necessary for the best coating or reported seed coating with SiNP-embedding film, to the best of our knowledge. Our approach of seed coating using a fluidized bed granulator resulted in a homogeneous alginate film with or without embedding biogenic SiNPs, as revealed by SEM analysis (**Figure 7**). The preliminary optimization of the film properties necessary for an optimal coating significantly improved the process. Our approach is the first of its type to be reported in the scientific literature, to the best of our knowledge.

### 4.2 Biostimulant activity of embedded SiNP seed coating

#### 4.2.1 The AGS film and its formulation with SiNPs show biostimulant effects on mung seedlings

In the absence of saline stress, the AGS film (variant V0) significantly increases the shoot and root length, and the addition of SiNPs in the AGS film (variants V1–V4) does not induce other significant changes compared with the V0 variant (**Figure 8A**). The AGS film does not significantly affect the activities of α-amylase, the antioxidant enzymes, SOD and G-POX, but the levels of lipid peroxidation (TBARS) and chlorophyll are slightly decreased; the carotenoids, GR activity, and H_2_O_2_ level are slightly increased in the absence of salt. SiNPs do not induce further changes in α-amylase, TBARS, G-POX, and SOD, except for the SOD activity, which increases slightly at 3% and 4% SiNPs (**Figure 10C**). The l-Pro is slightly decreased by the AGS film, but not by SiNPs (**Figure 10A**). A lower l-Pro level than for the control is observed in the presence of salt as well.

The most significant effects of the AGS film in the absence of salt are 1) the decrease of the specific extracellular H^+^ level (seH^+^) into the medium, an effect that is alleviated by SiNPs in a dose-dependent manner, reaching control values at 3% and 4% SiNPs (**Figure 9B**), and 2) the increase of NO level. The overall metabolic activity quantified by the MTT assay also shows a decrease in the presence of AGS compared to the control in the absence of salt. There is no further significant change in the MTT assay in the presence of SiNPs. The MTT test is based on the reduction of the yellow-colored compound MTT to a dark-blue formazan (Pouvreau et al., 2013). The reduction of MTT by mitochondrial enzymes (especially succinate dehydrogenase) is an index of cell/mitochondrial integrity.

The most significant effects of SiNPs in the absence of NaCl are on the chlorophyll content, which increases, on the specific extracellular H^+^ level, in which case it reverses the effect of the AGS film at SiNP concentrations higher than 2% in the film (**Figure 9**), and, on the GR activity and H_2_O_2_ level that significantly decrease in the presence of SiNPs (**Figure 10**). There is a hormetic behavior of the carotenoids induced by SiNPs in the absence of salt. With respect to NO, the SiNPs first induce a decrease at 1% SiNPs, followed by an increase at higher SiNP concentrations.

The tested NaCl concentration of 50 mM significantly affected almost all parameters measured, acting as an abiotic stress factor: the shoot length, the root length, chlorophyll, carotenoids, and the metabolic activity decreased. l-Pro, the lipid peroxidation, and ROS level significantly increased. The α-amylase slightly decreased, but not statistically significant.

Under saline stress, most of the biochemical and physiological effects of the AGS film and SiNPs on mung seedlings are much more significant than in the absence of salt. The only parameter that is not changed by AGS or SiNPs compared with the control is the α-amylase (**Figure 8B**). The coating effects on the seedling growth are not as significant as in the absence of salt, and the growth of seedlings does not reach the unstressed control (**Figure 8A**). Starch is a mixture of amylose and amylopectin, which is found as insoluble granules in chloroplasts (temporary storage) and amyloplasts (long-term storage). During the metabolism of starch, metabolic energy is produced by the enzymatic removal of glucose from the non-reducing ends of the amylose and amylopectin cluster, with α-amylase being one of the most important enzymes responsible for starch hydrolysis (Bernfeld, 1951; Hostettler et al., 2011; Tomasik and Horton, 2012). All other parameters, photosynthetic pigments, metabolism, the l-Pro level, lipid peroxidation, the activities of the antioxidant enzymes, and ROS level are significantly influenced by both the AGS film and SiNPs. Lipid peroxidation involves the attack of free radicals against lipids, particularly polyunsaturated fatty acids (PUFAs) (Ayala et al., 2014).

There are several correlations between parameters in both the absence and especially the presence of salt stress, as determined by Pearson’s correlation analysis (**Supplementary Tables 8, 9**). In the absence of salt, the growth parameters correlate negatively with l-Pro, TBARS, leaf ROS, and total ROS; the pigments correlate positively with eH^+^ and negatively with GR activity and leaf ROS, the last two being positively correlated to each other; MTT correlates positively (+) with seH^+^ and negatively (−) with G-POX; l-Pro, TBARS, and ROS correlate positively; H_2_O_2_ correlates positively with GR activity. These correlations are in general straightforward: ROS induce lipid peroxidation (TBARS), production of l-Pro, and activation of GR, which induces the production of the antioxidant GSH. Stress and consequently ROS affect growth and metabolism, which are positively correlated with each other. It is worth mentioning that chlorophyll *b* correlates (+) additionally with seH^+^ (marginally significant), which indicates a higher degree of correlation of chlorophyll *b* with the metabolism of the seedling than for the other pigments. In the presence of salt, the number of correlations increases. The growth parameters correlate additionally (+) with the photosynthetic pigments, NO, SOD, and G-POX and (−) with H_2_O_2_ and GR activity; α-amylase correlates (−) with H_2_O_2_; the pigments correlate additionally (−) with l-Pro and TBARS; SOD, G-POX, and NO are (+) correlated with each other and correlate (−) with ROS and GR. MTT retains only the (+) correlation with seH+, both of them being non-significantly correlated with the other parameters. CAT activity does not correlate significantly with any of the other parameters. The correlations confirm the expected outcome, i.e., the salt stress induces ROS that affect the seedling growth and metabolism, activating the defense mechanism represented by l-Pro, GR activity, and CAT. The film coating efficiently reduces ROS by activating the antioxidant enzymes SOD and G-POX and significantly induces NO production while reducing the l-Pro content and GR activity, whereas the CAT activity remains high when the film with SiNPs is applied. In this context, in addition to the ROS level, increased GR and l-Pro can be seen as markers for saline stress, whereas increased SOD, G-POX, and pigments, accompanied by reduced ROS, indicate biostimulant activity. The CAT activity has a rather heterogeneous behavior.

Most of the effects observed are in accordance with recently reported works involving Si and alginate (Al Murad and Muneer, 2022; Mir et al., 2022; Mukarram et al., 2022) and/or as expected, although there is no similar formulation available until now, to the best of our knowledge. In the case of Si, there is an increased interest in its application under different formulations to mitigate the abiotic stress on plants, including salt stress. Recent studies investigated the effects of either radicular or foliar application on different plant species and cultivars at different stages of development, with limited information on the effects induced from the first stages of development, at least in the case of beans (Farhangi-Abriz and Torabian, 2018; Ahmad et al., 2019; Naguib and Abdalla, 2019; Rady et al., 2019; El Moukhtari et al., 2022; Shen et al., 2022; Ellouzi et al., 2023; Peña Calzada et al., 2023). Dhiman et al. and El Moukhtari et al. recently reviewed the multifaceted and interesting effects of Si on plants and the missing links for fully understanding the mechanism of action (Dhiman et al., 2021; El Moukhtari et al., 2022). Si applied as sodium silicate or commercial chemical SiNPs increased the salt stress tolerance of *Lathyrus odoratus* by seed priming, inducing lower levels of proline, lipid peroxidation, ROS, and Na^+^ uptake in plants and increasing the antioxidant enzymatic activities and K^+^ and K^+^/Na^+^ ratio (El-Serafy et al., 2021). Similar results were obtained in the case of *Hordeum vulgare* L (Ellouzi et al., 2023). Most studies, including on mung beans, obtained similar results, except when it comes to the level of proline, which either decreases or increases in the presence of Si. Some other differences compared with other studies, such as the effect on the antioxidant enzymes, were found in the case of cucumber, where the enzymatic activities were decreased by Si compared to control (Gou et al., 2020). There is no systematic experimental comparison between plant species, timing, seedling age, and Si forms of application, to the best of our knowledge, and many studies do not make a comparison between Si effects in the absence and presence of salt stress in order to distinguish between biostimulant and other types of effects. In our case, AGS in particular and also SiNPs decrease the lipid peroxidation and the l-proline content, with saturation at 3% SiNPs. AGS and SiNPs partially compensate the decrease in the metabolic activity and seH^+^, but SiNPs present a maximum at 1%–2% after which the dose becomes too high or other mechanisms compensate and the effect reverses. There is an increase of SOD and G-POX activities induced by AGS and further by SiNPs with saturation after 2% SiNPs. The increase of the antioxidant enzymatic activities is negatively correlated with the ROS level, except for the GR activity, which is positively correlated. Silicon was shown to be involved in the activation of enzymes that lead to ROS scavenging and consequent decrease of lipid peroxides content, improvement of membrane integrity, and prevention of electrolyte leakage (Liang et al., 2003, 2006) and also in increasing the activity of enzymes involved in respiration processes for a higher ATP content to be hydrolyzed by specific enzymes for nutrient uptake (Zhang et al., 2011).

The data indicate that the AGS film and SiNPs act as plant biostimulants when applied as a homogeneous coating film on mung seeds using a fluidized bed granulator [bottom-spray (Wurster) process]. Most effects of AGS and Si are more significant and with a higher degree of correlation under salt stress than under normal conditions in our study.

#### 4.2.2 The AGS film might have a multi-action effect on metabolic activity

The decrease of seH^+^ and overall metabolic activity induced by AGS alone in the absence of salt and the decrease of seH^+^ by NaCl was a somewhat surprising result. Some of these data cannot be compared with other studies due to the lack of information in this respect. Our hypothesis is that we could have in fact several types of actions induced by AGS that overlap, increasing the complexity of the system: 1) a possible initial effect of (osmo)priming induced by the film before germination, probably correlated with the biostimulant effect observed; 2) a weak stressor effect during germination induced by the film; and 3) a possible plant growth promotion effect of the film.

Due to their capacity to reduce the water potential, sorbitol and glycerol are two of the organic compounds used for seed osmopriming, by which seeds are treated with these osmolytes and then washed, and the seeds are dried before germination. In this way, the pre-germination metabolism is activated by small doses of available water, and the germination, plant growth, and tolerance to stress are improved (Melekote Nagabhushan et al., 2022). As the equilibrium between the positive and negative activities of these polyols depends on their concentration, usually, concentrations up to a maximum of 100 mM, depending on the context, are used for obtaining osmoprotectant effects. Higher concentrations can exert toxic effects, and the osmolytes can be used to simulate drought stress, but the effects are not as pronounced as those of PEG, and sorbitol was reported to upregulate the antioxidant enzymes in rice leaves (Hsu and Kao, 2003). In some cases, negative and positive effects were reported in the absence and presence of other stress forms, respectively (Wang et al., 2022).

The positive outcome observed in our study on the plant development indicates an (osmo)priming effect of the AGS film. After germination, the AGS film is dissolved in the medium, and the organic components probably diffuse slowly and gradually through the agar medium away from the seed. Therefore, we believe that the direct effect should be less pronounced than the (osmo)priming effect. All three components of AGS could contribute to this priming effect, the organic compounds from the film by inducing a low external water potential (osmopriming) and alginate additionally by the presence of small quantities of Na^+^, acting as a weak stressor. Seed priming by osmotic adjustment (osmopriming) is already a commercially available method and was recently reviewed (Melekote Nagabhushan et al., 2022).

The inhibition of the metabolic activity could be the result of a certain degree of stress level of the AGS film (maybe a combination of osmo-stress and Na^+^ stress from alginate), but the stress does not surpass the beneficial effects. In the presence of salt, the AGS film has a different behavior, slightly alleviating the metabolic inhibition induced by salt. One explanation could be that the film, especially alginate, retards the Na^+^ diffusion to the plant cells. More optimized compositions in the future, depending on the contribution of each component, could have even better effects.

Proton pumping seems to be involved in osmoregulation, and exogenous osmolytes have been shown to affect membrane permeability, activity of proton pump enzymes, and proton efflux (Reinhold et al., 1984; Lew and Spanswick, 1985; Van Bel and Patrick, 1985; Shanko et al., 2003; Chiu et al., 2006), but the subject has not been sufficiently investigated. The effects of osmolytes on proton pump enzymes investigated *in vitro* seem to be rather an indirect process resulting from changes in membrane structure (Chiu et al., 2006). Proton pump activity can be considered a marker of membrane integrity and plant metabolism status. In order to maintain the transport of different mineral nutrients in symport with H^+^ ions, proton pumps use the ATP energy (Zhang et al., 2011; Cosse and Seidel, 2021).

The effects of salt on the activities of proton pumps (PPs) either from the plasma membrane (PM) or from tonoplast (T) are in some cases contradictory. Most of the studies available until now reported stimulation of PM and T proton pumps (Kłobus and Janicka-Russak, 2004; López-Pérez et al., 2009; Janicka-Russak et al., 2010, 2013), but some studies reported no effect (in the case of salt-resistant plant species) or inhibition of proton pumps, which was time-dependent in some cases (Shan'ko and Babakov, 2002; Zörb et al., 2005; Kabała and Kłobus, 2008; Pitann et al., 2009; Wakeel et al., 2010). Tonoplast communicates with the plasma membrane through the cytosol, and the processes in one can affect the processes in the other (Horaruang et al., 2020). We have to emphasize here that most quantitative PP activities have been investigated *in vitro*, either in extracted vacuoles or in plant tissue sections. There are no quantitative assays available for *in vivo* estimation of PP activities, but only qualitative (the acidification of agar medium) on which our semi-quantitative assay is based as well. This assay, based on medium acidification, reflects the interplay between the activities of several ion transporters (proton pumps and Na^+^/H^+^ exchangers), membrane passive diffusion, and other *in vivo* processes, which could affect the H^+^ equilibria. The assay has not been tested before for investigating the effects of salt stress or osmolytes, to the best of our knowledge. Under high levels of exogenous Na^+^ ions, both the proton pumps and Na^+^/H^+^ antiporters are upregulated (Miranda et al., 2017). The PM proton pumps expel H^+^ from the cells and maintain the PM potential gradient necessary for the Na^+^/H^+^ antiporter (SOS1) to efficiently exchange extracellular H^+^ with intracellular Na^+^.

Previous studies suggest that an excess of salt inhibits the proton pump due to an increased level of ROS and lipid peroxides that have a negative effect on membrane integrity (Liang et al., 2006; Cuin et al., 2011). Moreover, continuous active extrusion of Na^+^ would increase the gradient, leading to a more and more energetically expensive process cycle that in the long run will amplify salt toxicity and irreversibly affect the plant. Halophytes might be able to better adapt and tolerate salt stress by being more capable of reducing the influx and/or sequestering Na^+^ in vacuoles rather than expelling Na^+^ through the plasma membrane (Cuin et al., 2011).

In our study, we established a semi-quantitative *in vivo*-induced seH^+^ independent of root area, using medium acidification and ImageJ analysis. Both the AGS film and NaCl induced inhibition on the specific activity when applied individually, but the total acidification of the medium was inhibited only by NaCl due to the reduced root area. The AGS has a positive effect on root length, which compensates for the reduced seH^+^, and as a consequence, the overall medium acidification is similar to the control. In the case of the AGS film, the small quantities of Na^+^ ions present in alginate could be partially responsible for the reduced seH^+^ observed. We propose that the extracellular H^+^ level is the result of the fine regulation between proton pumps and Na^+^/H^+^ antiporter, i.e., the level by which each of them is stimulated and the new equilibria that are established in time. In our case, the intracellular uptake of H^+^ through the antiporter seems to overpass the extracellular efflux by proton pumps in the presence of the AGS film or NaCl. Our hypothesis is that during the initial phases of salt stress, the plant is able to keep the pH gradient necessary for Na^+^ efflux from the cells, but as the stress is prolonged or becomes more severe, the pH gradient is reduced probably because of changes in transporter activities. There is a need for more in-depth studies and more precise *in vivo* assays in order to accurately establish the overall effects. Mathematical modeling and system biology could also significantly contribute in this respect (Horaruang et al., 2020).

Alginate is usually applied to plants as alginate oligosaccharides and more often by foliar spraying than by root treatment. Alginate oligosaccharides can be absorbed in plants and act as internal modulators of plant metabolism. These applications usually lead to increased metabolic activity and increased l-Pro levels. Only a few reports on alginate used as seed coating exist until now, and not all of them refer to metabolic activity. Therefore, a direct comparison with other studies is difficult to make.

In our case, the short time frame of the experiment (4 days) probably resulted in only small quantities of alginate oligosaccharides, considering also the lack of microorganisms in the agar medium and previous reports that claim it takes 120 days for 20% of alginate-based hydrogels to be decomposed in soil (Song et al., 2020). Therefore, if alginate contributes to the coating effects observed, the mechanism could be different than the mechanisms reported for alginate oligosaccharide-based bioproducts, especially since the latter have been mainly applied foliarly. As alginate resides close to the seed during germination and growth due to its application as a coating film and swells in the presence of water, it could retain water close to the seed, at least during the first stages of germination. How much water was available for the seed was difficult to estimate. The observation that the AGS film induced an increase in the root length without an increase in the α-amylase activity, in the absence of salt, can be explained and supports the idea that the alginate polymer acted mainly by enhancing the water-holding capacity of the seeds rather than inducing α-amylase activity. The seed germination and radicle growth depend on water availability (Bewley, 1997). Water-absorbent polymers regulate water availability to seeds and water uptake (Pedrini et al., 2017) and improve seedling/radicle growth (Pathak and Ambrose, 2020; Amirkhani et al., 2023). Alginate-derived oligomers enhance germination by amylase induction (Hu et al., 2004). The effect on the root length under salt stress was only marginally significant and more correlated with the α-amylase activity than in the absence of salt, for both the AGS film and SiNPs. In the case of alginate, the specific contribution each of the of priming/biostimulant, weak stressor, and/or plant growth promoting activity to the overall alginate effect is less evident. Nevertheless, it would imply that alginate could have positive effects on plants even in the absence of generation of significant amounts of oligosaccharides. The few previous studies that applied intact alginate for a short period of time support this hypothesis, with alginate having, for example, positive effects on sweet corn (*Z. mays* var. *saccharata*) germination in the absence or presence of osmotic stress (Behboud et al., 2021).

A recent study used alginate films reticulated with Ca^2+^ ions to coat bean seeds (*Phaseolus vulgaris* L.) (De Castro et al., 2020). In comparison to our film coating, the crosslinked film delayed seed germination, which indicates that reticulation has important disadvantages. They speculated that the delay observed is related to the film forming a barrier that decreases the absorption of H_2_O and the passage of O_2_. STEM EDX analysis revealed some contents of Ca^2+^ in the alginate used for SiNP dispersion and for coating (**Figure 4F**), but the amounts are not sufficient to significantly reticulate the film. Although our film is probably able to quickly break down at germination because it is not significantly reticulated, we believe that it interacts with and slows down the transport of exogenous Na^+^ during germination and seedling growth process, therefore slightly alleviating the drastic decrease in metabolic activity induced by salt. The mechanism could involve changes in the surrounding environment of the roots and would be different than that of alginate oligosaccharides that are able to be transported into plants, influencing the physiology of the plants.

Our results support the hypothesis that in the absence of stress, alginate acts as a proton pump inhibitor. One explanation could be the formation of a polymeric barrier consisting of chains of β-d-mannuronic (M) and α-l-guluronic (G) acids linked by glycosidic bonds 1→4, monomers that are sequentially distributed in each repetitive subunit (Loureiro Dos Santos, 2017). In the presence of Ca^2+^, alginate forms a hydrogel with an open network, in which divalent cations interact cooperatively and ionically with the G monomers (Bierhalz et al., 2014). Therefore, this polymeric barrier could inhibit the proton pump conformational changes and the homeostasis of the whole system. Additionally, a hypothesis for counteracting the effect produced by alginate, by including SiNPs in the seed coating, could involve the intercalation polymerization of silicic acid and the formation of large chains that lose the ionic bonds established between Na^+^ ions and alginate, and dislocate Na^+^ ions, acting as a crosslinker that creates gaps in the polymeric network, allowing the pump to resume its activity.

#### 4.2.3 The effects of alginate-SiNP biostimulant on the antioxidant defense system seem to depend on multiple factors

With respect to l-Pro, the induced decrease by AGS could have two explanations: 1) osmoprotection by the sorbitol and maybe glycerol uptake from the medium into the seedlings, so the seedlings do not need a high level of l-Pro, and 2) the form of alginate application, e.g., as intact alginate and application at seed. As mentioned above, alginate could act directly on the availability and transport of sodium ions and therefore could reduce the saline stress, therefore reducing the l-Pro needed to protect the plant. The application of alginate oligosaccharides usually leads to an increase in l-Pro levels. It is also possible that alginate alone could increase l-Pro and that this effect is overcome by the opposite effect of sorbitol and glycerol.

An alternative or simultaneous mechanism in the case of l-Pro could be related to the time frame of the treatments, the development and metabolic stage of the seedlings, and the type of the seeds. This hypothesis comes from previous and present observations related to the effect of Si on l-Pro levels in the presence of saline stress, which could also apply to alginate. The influence of Si on l-Pro is somewhat controversial. In the literature, there are reports of both increased and decreased accumulation of l-Pro following treatment with Si under salt or drought stress (Zhu et al., 2020; Basilio-Apolinar et al., 2021). Research on okra (*Abelmoschus esculentus*), *Glycyrrhiza uralensis*, *P. vulgaris*, and *Capsicum annuum* found that Si protected the plants against saline stress by inducing higher content of free l-Pro (Soares Pereira et al., 2013; Abbas et al., 2015; Rady et al., 2019; Cui et al., 2021), but the decreased level of l-Pro induced by Si was reported for wheat and soybean (Lee et al., 2010; Bybordi, 2015; Singh et al., 2022). In a recent study, the modulation of l-Pro by Si in cucumber seedlings was found to be time-dependent, i.e., L-Pro increased compared to control during the first days after NaCl + Si treatment, and started to decrease 6 days after the treatment (Zhu et al., 2020). The authors speculated that Si first acted by one mechanism, boosting the l-Pro content to alleviate the salt stress, after which, probably because of the alleviation of stress injury, a new metabolic equilibrium might be established to keep l-Pro at adequate levels in plant tissues. The decrease in l-Pro could be also an outcome of the inhibition of Na^+^ and Cl^−^ uptake by Si, an inhibition reported in previous studies (Shi et al., 2013; Zhu and Gong, 2014; Al Murad and Muneer, 2022). l-Pro, an essential amino acid, is involved in various signaling pathways under stress conditions (Liang et al., 2013), in addition to its osmoprotection function. In our case, alginate and SiNPs induced a decrease of l-Pro in mung beans 4 days after germination, when the treatments were applied before germination. Therefore, we propose that there is more than one mechanism of action for alginate and Si and that multiple parameters are involved and have to be taken into consideration: plant type, mode of biostimulant application, plant development stage, time frame of biostimulant action, etc.

Complex behavior can be found also in the antioxidant enzymes. SOD, G-POX, and CAT are some of the main enzymes involved in ROS scavenging. SOD leads to the production of hydrogen peroxide through the dismutation of the superoxide anion. Hydrogen peroxide is subsequently converted by G-POX and CAT into water and oxygen (Liang et al., 2003). GR, however, contributes to maintaining ROS homeostasis by keeping the balance between the oxidized (GSSG) and reduced (GSH) forms of glutathione, thereby using glutathione for parallel scavenging (Couto et al., 2016). As several enzymes are implicated in keeping ROS levels under control, their gene expression is tightly regulated, and each enzyme is more or less active depending on the context. In the literature, there is no homogeneous trend in the behavior of the enzymes, and it is difficult to model all situations in a general systemic model due to the multitude of different conditions. The antioxidant system is in continuous dynamics, and processes such as germination and seedling establishment impose significant changes (Chen and Arora, 2011). (Osmo)priming and biostimulants have significant effects on the antioxidant enzymes. Why certain enzymes are sometimes activated while others with similar functions are inhibited is not yet very clear at the mechanistic level.

Most of our data are as expected; almost all activities are kept at the base level of the control irrespective of coating in the absence of salt. The reduction of H_2_O_2_ in the absence of salt did not need the activity of catalase; this task is accomplished by GR, which maintains a high GSH/GSSG ratio, G-POX, and probably other enzymes involved in the glutathione–ascorbate cycle such as ascorbate peroxidase. Moreover, from Pearson’s correlation, the H_2_O_2_ level was significantly correlated with the GR activity. Interestingly, salt stress activated catalase and slightly inhibited G-POX. This is probably because catalase is much more efficient than G-POX, reducing two molecules of H_2_O_2_ in one reaction. Catalase was reported to be the most responsive enzyme at pretreatment of maize with H_2_O_2_ (Gondim et al., 2012), indicating that high levels of H_2_O_2_ need high expression of catalase. Increased CAT activity was obtained previously for six genotypes of mung bean treated with 100 and 150 mM NaCl, and the CAT activity decreased at 200 and 250 mM NaCl (Alharby et al., 2019). In another study, 150 mM NaCl induced inhibition of all enzymes in mung seedlings when analyzed after 18 days of seedling growth (Dong et al., 2022), which could suggest that the plants are severely affected at high and/or long salt stress.

The reduction of CAT and increase of G-POX activity in AGS-coated seedlings compared to control, under salt stress, could be related to the proposed reduced availability of Na^+^ ions to plants, (osmo)priming effects, and/or biostimulant effects of AGS. Practically, the seedling condition seems to almost return to the control in the absence of salt. SOD seems to be more sensitive, as this enzyme is more responsive to the positive effect of AGS than the other two enzymes. This is probably because superoxide is more harmful than H_2_O_2_, and it is critical to be kept under control. SOD is responsive to the highest doses of SiNPs tested (3%–4%) in the absence of salt (**Figure 10D**). An intriguing effect is the reduction of the GR activity by SiNPs. Previous studies reported a decrease, an increase, or no change of GR by Si in plants at different development stages and subjected to abiotic stress, such as salt, draught, and/or heavy metals (Siddiqui et al., 2014; Zhang et al., 2017; Das et al., 2018). In fact, most antioxidant enzymes have a heterogeneous behavior previously reported by various studies, and the exact causes and mechanisms behind these observations are not fully elucidated. In our case, future planned, more in-depth investigations hopefully will give more answers. As mentioned before, SiNPs were very efficient in stimulating all the other enzyme activities and reducing the ROS levels.

Considering the sizes of the SiNPs obtained in this study, the effects observed were probably induced mostly by the released Si(OH)_4_, as observed in **Figure 5**. As mentioned in the Introduction, the classical belief that only the soluble forms of Si can be translocated to plants has been challenged by new studies that propose translocation of small NPs as well (Kurczyńska et al., 2021). The average sizes of our SiNPs would make most of them difficult to translocate to the seedlings. Nevertheless, we proved that the seedlings do get enriched in Si upon coating the seeds with SiNPs compared with the controls without SiNPs (**Table 2**); therefore, the translocation definitely exists under one form and/or another.

There is a need for more in-depth studies in order to fully understand the mechanism of the different components of our tested formulation. Transcriptomics and proteomics are promising tools that can provide information on the interactions and effects at the molecular level (Dhiman et al., 2021), which we plan to address in the following studies. It is also important to continue the investigation in real-life conditions such as the coated seeds planted on a growth substrate, grown in greenhouses or on fields, in order to assess the full effects of bean production yield and quality under the possibility of having multiple abiotic and biotic stress forms. As we have recently shown, we are able to control the thickness of the film (Trica et al., 2023), and optimizing the thickness, but also the SiNP dose, might give an even better output than the one presented in the current study.

## 5 Conclusions

Amorphous SiNPs with 99% purity, of approximately 50 nm, with Si−O−Si and Si−OH bonds, 0.347 cm^3^/g CPV, and 240 m^2^/g SSA, were biosynthesized from RH and embedded in an AGS film optimized by mixture design. Using the Wurster process, we obtained a homogeneous AGS film with or without embedding biogenic SiNPs, as revealed by SEM analysis. The formulation of nanobiosilica in an AGS seed coating resulted in the alleviation of salt stress on mung seedlings by increasing the activity of enzymes involved in the plant defense system, decreasing the level of reactive oxygen species and lipid peroxides, compensating the necessary l-proline content, and increasing the photosynthetic pigments and NO content, as well as the specific extracellular H^+^ level into the medium, probably by re-establishing the equilibria of membrane transporter activities. We believe our data will have not only scientific but also important economic and social implications, from the recovery of RH biogenic SiNPs to its efficient application together with the beneficial alginate-based film, as plant biostimulants that alleviate saline stress from the first stages of plant development.

## Data availability statement

The original contributions presented in the study are included in the article/**Supplementary Material**. Further inquiries can be directed to the corresponding author.

## Author contributions

NT: Conceptualization, Investigation, Methodology, Writing – original draft, Writing – review & editing. BT: Data curation, Investigation, Methodology, Visualization, Writing – original draft. Ş-OD: Data curation, Investigation, Methodology, Writing – original draft. LC: Investigation, Writing – original draft. R-AG: Investigation, Methodology, Writing – original draft. AC: Data curation, Methodology, Writing – original draft. FO: Conceptualization, Funding acquisition, Project administration, Resources, Supervision, Writing – review & editing. DC-A: Conceptualization, Funding acquisition, Investigation, Methodology, Project administration, Resources, Writing – original draft, Writing – review & editing.
