# Supplementary figures and images for: Mechanistic insights into the plant biostimulant activity of a novel formulation based on rice husk nanobiosilica embedded in a seed coating alginate film
Source: Front Plant Sci. 2024 May 21;15:1349573. doi: 10.3389/fpls.2024.1349573 (PMC11148368; doi:10.3389/fpls.2024.1349573)

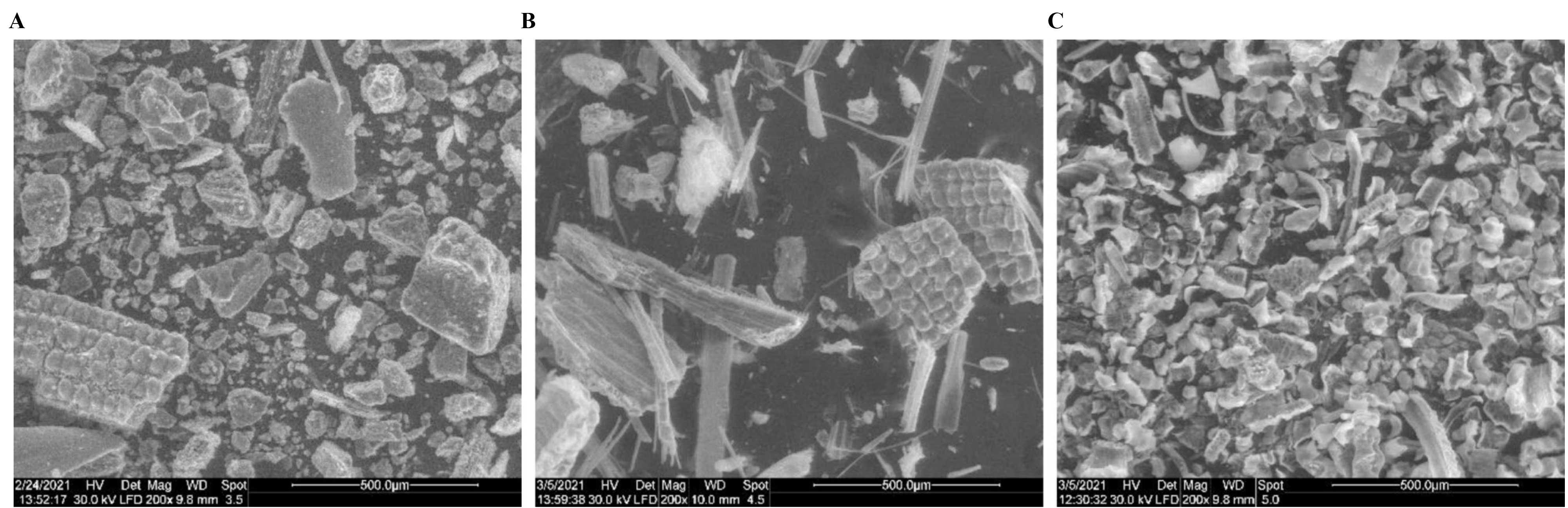

Supplement: Supplementary file 2 [file Image_2.tif]

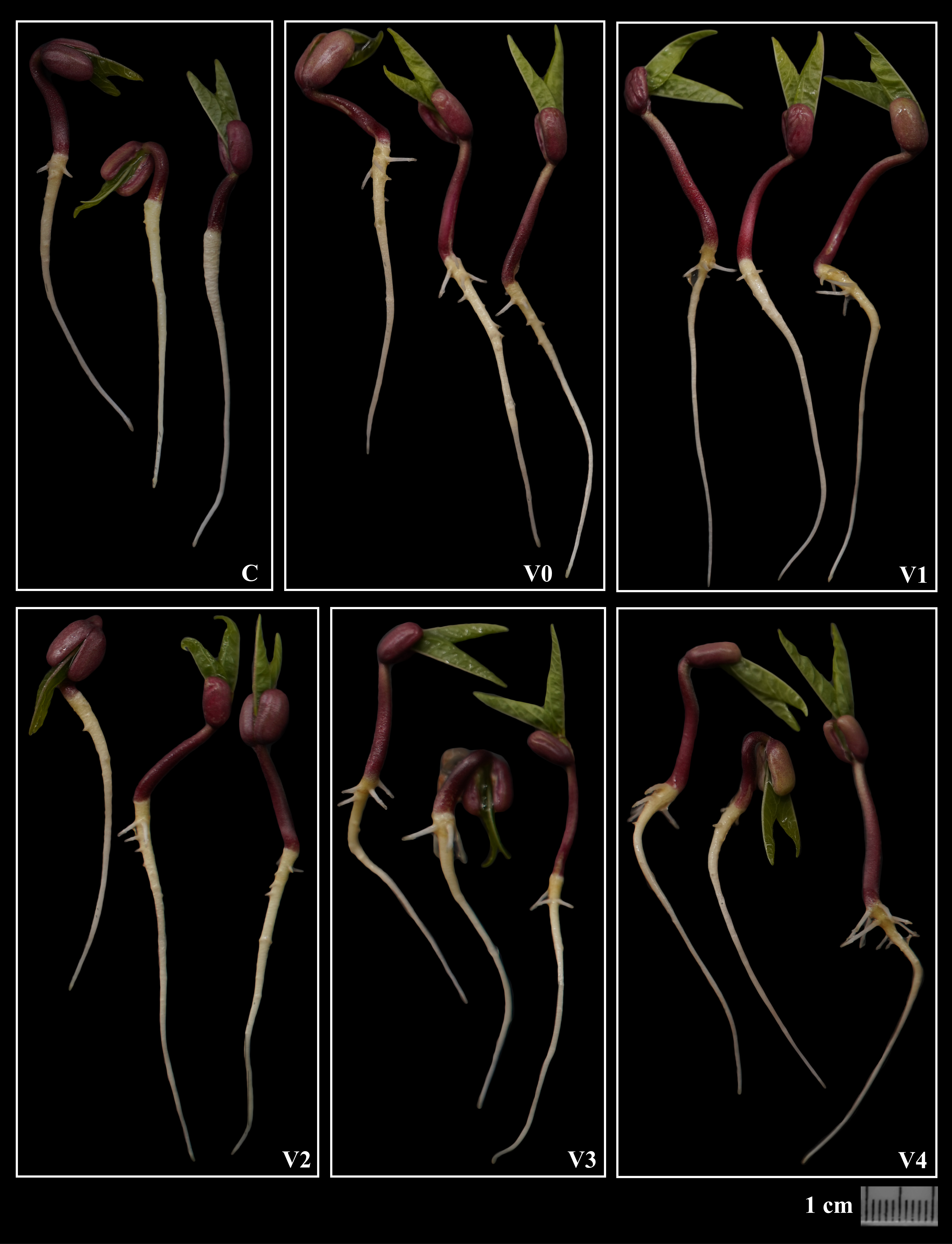

Supplement: Supplementary file 3 [file Image_3.tif]

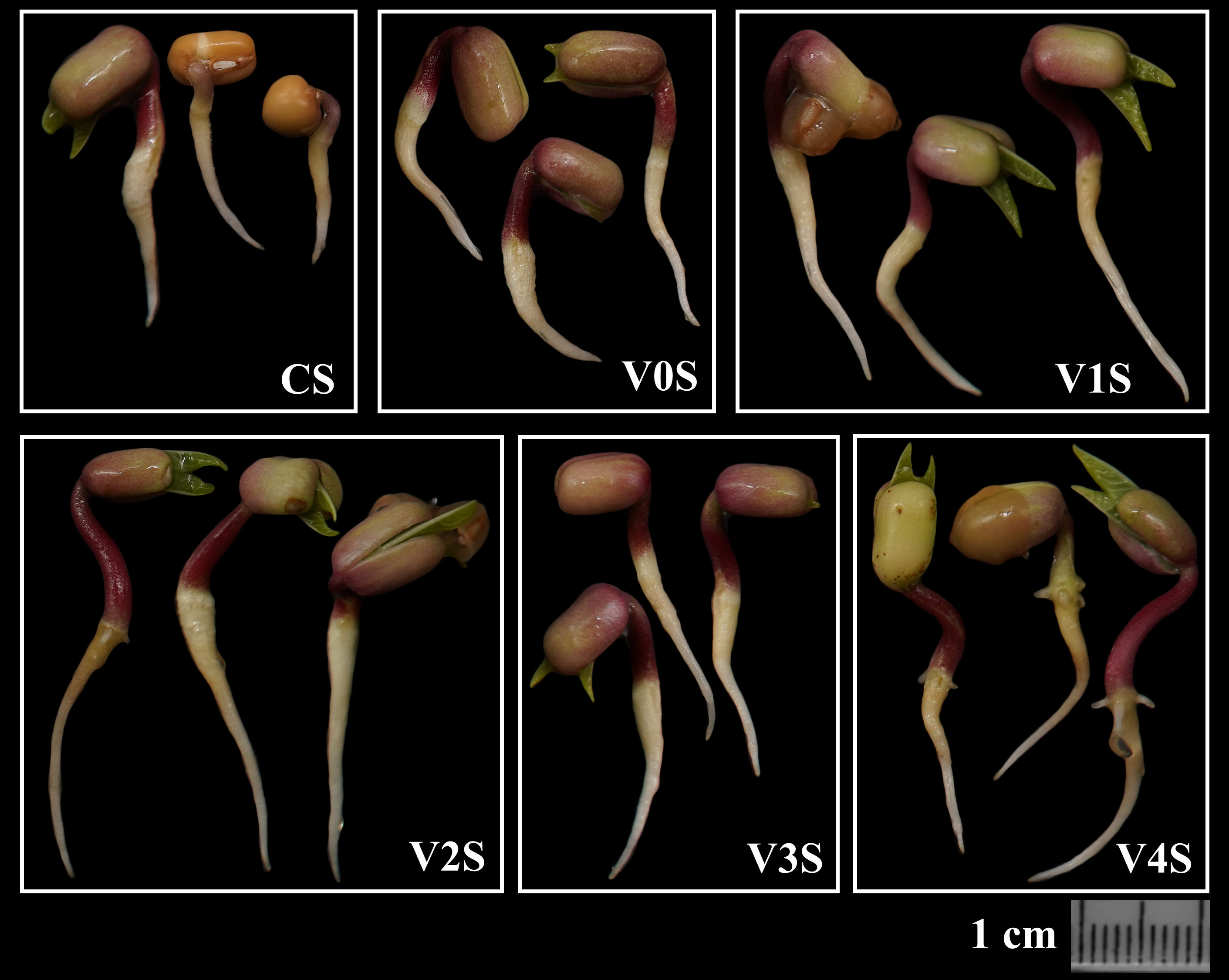

Supplement: Supplementary file 4 [file Image_4.tif]

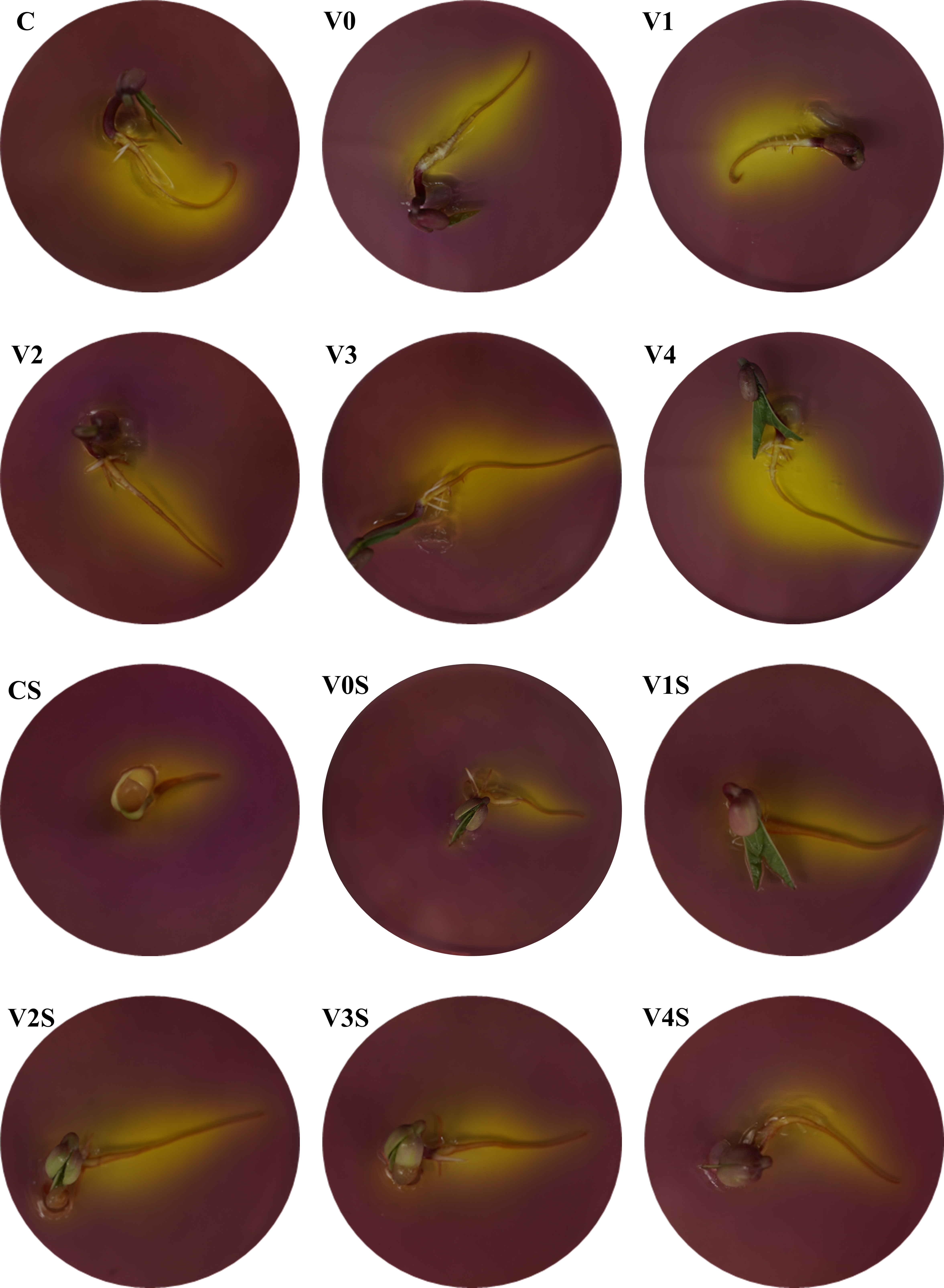

Supplement: Supplementary file 5 [file Image_5.tif]

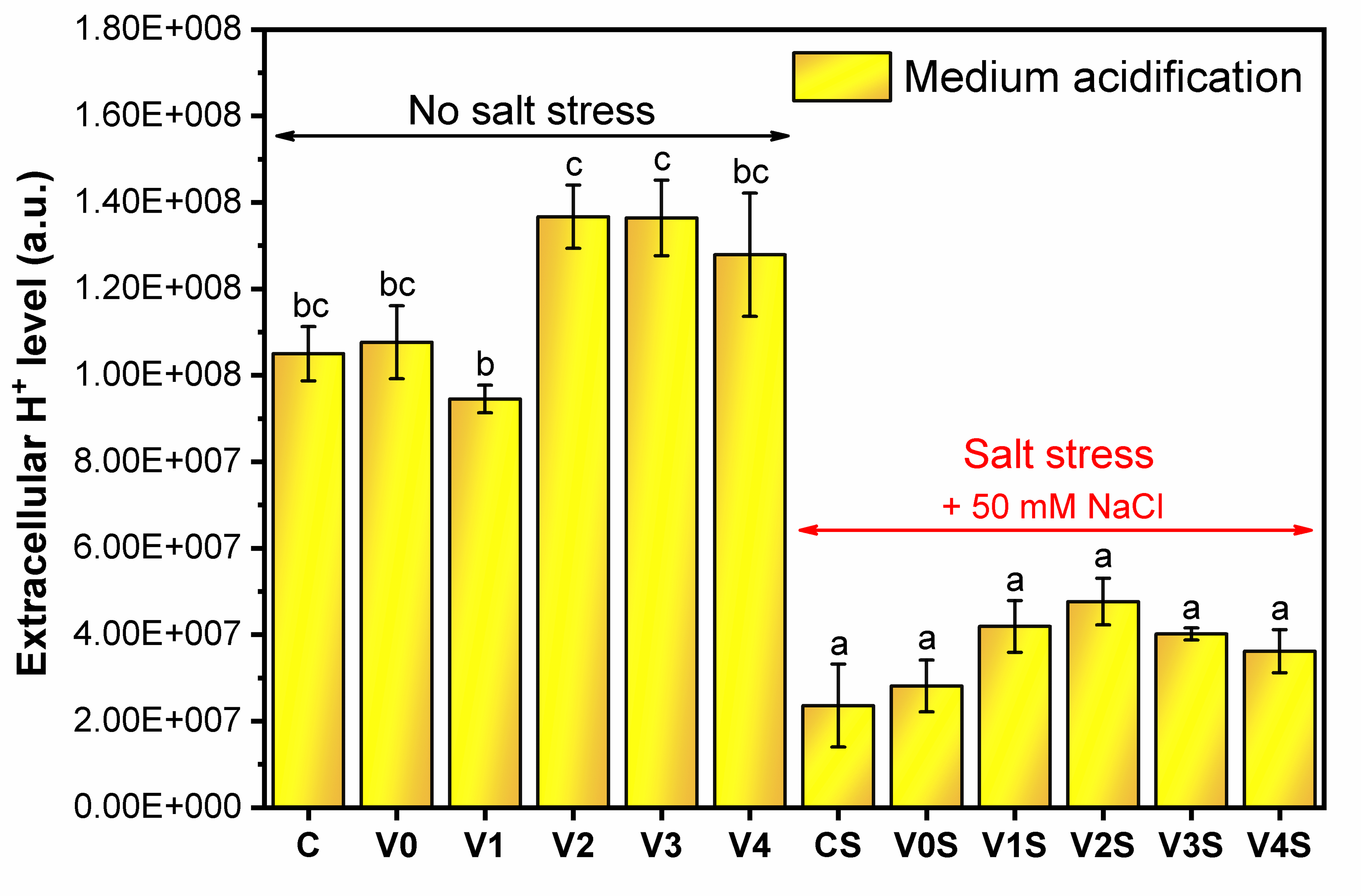

Supplement: Supplementary file 6 [file Image_6.tif]

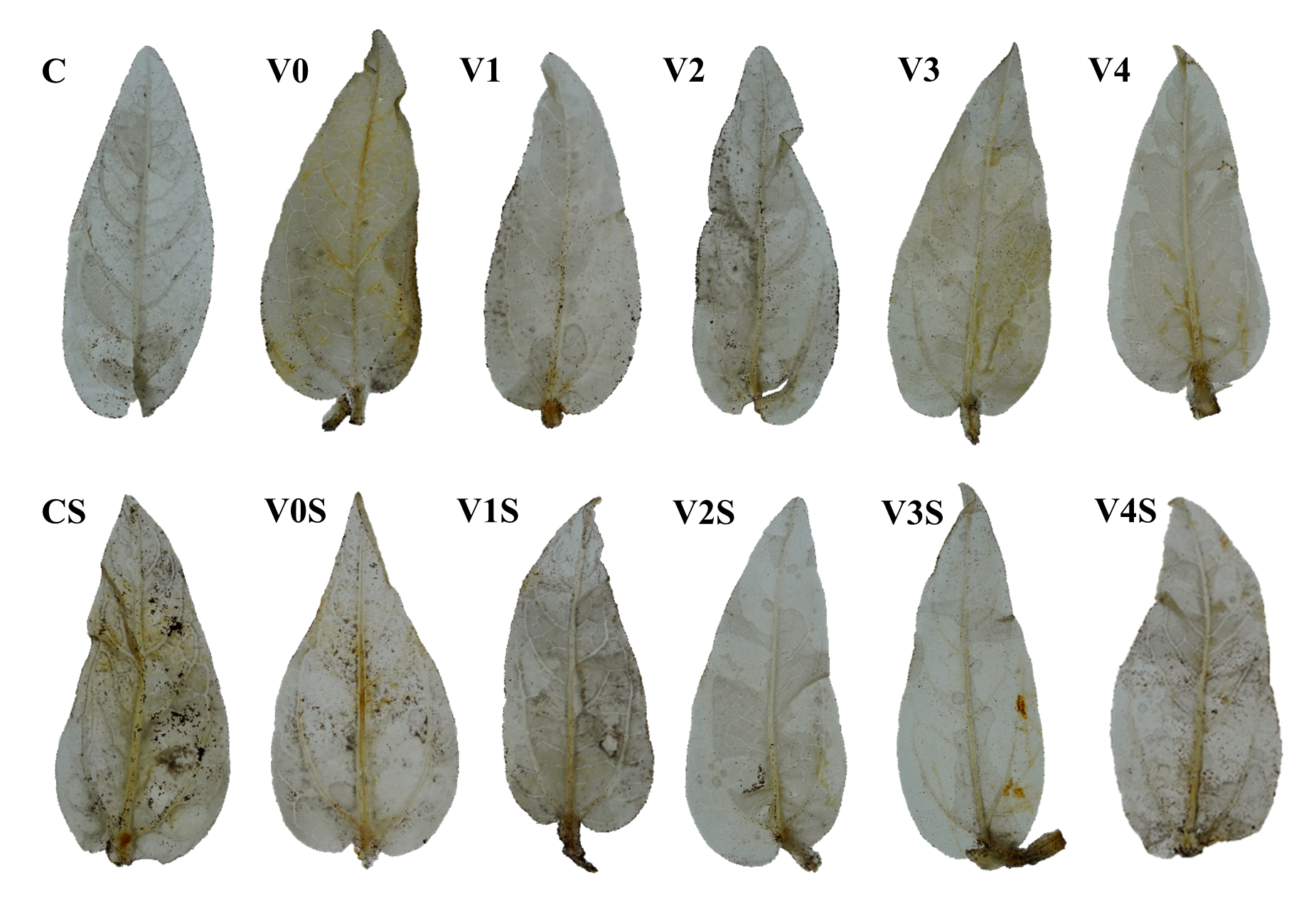

Supplement: Supplementary file 7 [file Image_7.tif]

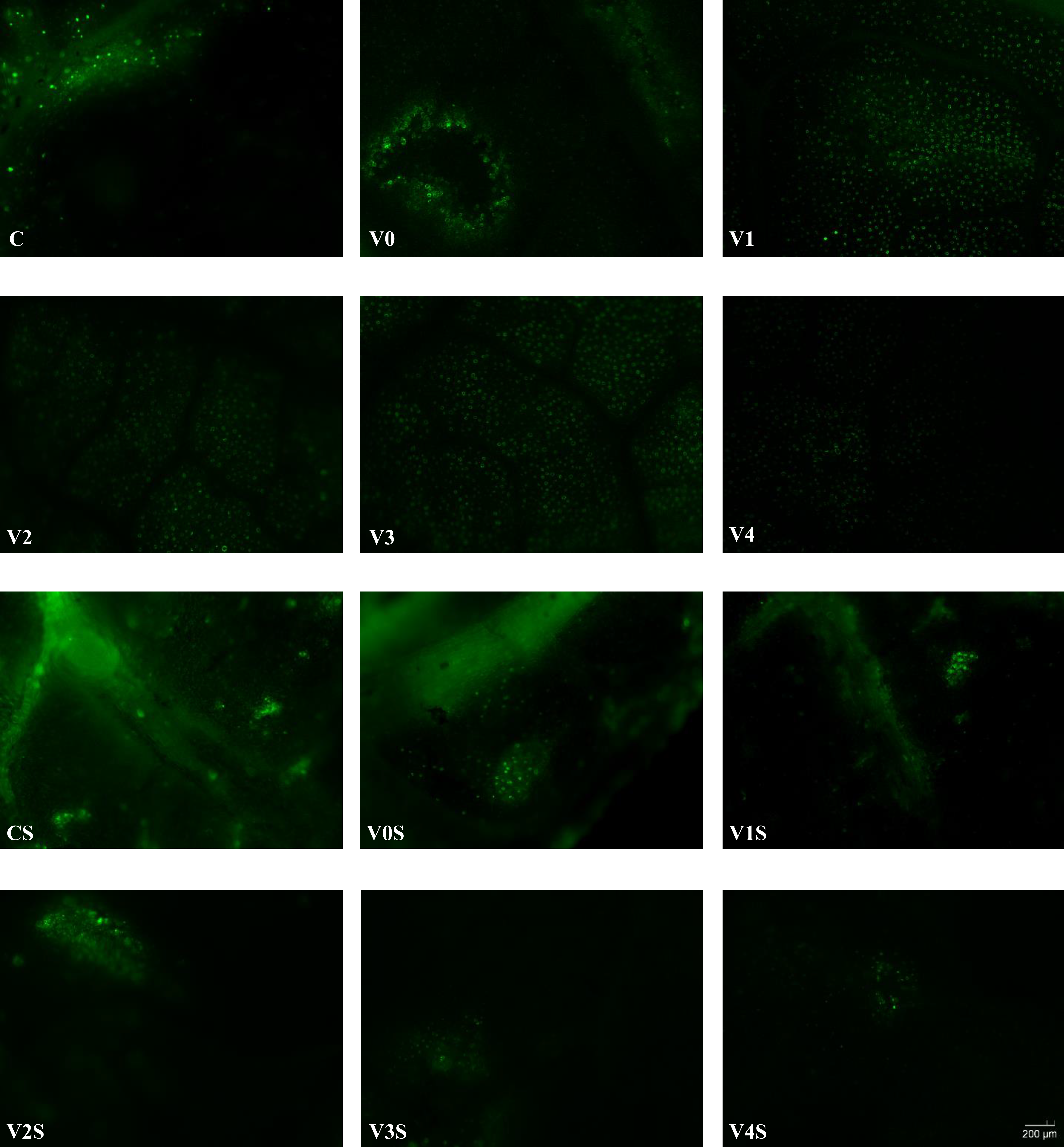

Supplement: Supplementary file 8 [file Image_8.tif]
