# Supplementary material for: Mechanistic insights into the plant biostimulant activity of a novel formulation based on rice husk nanobiosilica embedded in a seed coating alginate film
Source: Front Plant Sci. 2024 May 21;15:1349573. doi: 10.3389/fpls.2024.1349573 (PMC11148368; doi:10.3389/fpls.2024.1349573)
Supplement: Supplementary file 9 [file DataSheet_1.pdf]

Time : 2024-04-10 12:27:20

Serial Number : 803368

Method : Geochem(2)
Daily ID : 1

Elapsed Time : 30 s

Spectrum

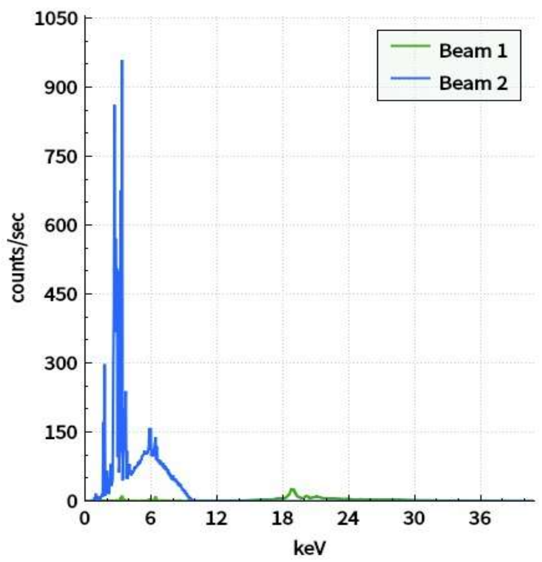

Aiming Image

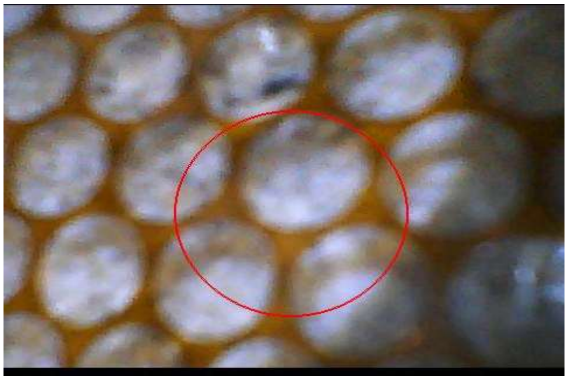

Chemistry

Elapsed time: 30.0s

El	PPM	+/- 3σ
Si	27.17%	0.88
P	2.30%	0.16
S	1.255%	0.094
K	12.00%	0.37
Ca	1.776%	0.091
Cr	650	260
Mn	2580	390
Fe	3320	350
Ni	91	56
Zn	98	32
Rb	10	7
Sr	16	7
Y	16	9
Zr	11	11
Nb	12	12
Ag	54	47
Th	61	38
LE	54.8%	1.3
El	PPM	+/- 3σ
Mg	ND	<61000
Al	ND	<11000
Ti	ND	<1200
V	ND	<110
Co	ND	<93
Cu	ND	<54
As	ND	<16
Se	ND	<9
Mo	ND	<32
Cd	ND	<100
Sn	ND	<140
Sb	ND	<170
W	ND	<80
Hg	ND	<46
Pb	ND	<25

....

Notes

info: u2

Date:

Signature:

Time : 2024-04-10 12:28:00

Method : Geochem(2)

Daily ID : 2

Serial Number : 803368

Elapsed Time : 30 s

Chemistry

Elapsed time: 30.0s

El	PPM	+/- 3σ
Si	27.64%	0.88
P	2.24%	0.16
S	1.262%	0.092
K	11.83%	0.36
Ca	1.724%	0.088
Cr	590	240
Mn	2440	370
Fe	3110	340
Ni	62	51
Zn	115	33
Rb	8	7
Sr	15	7
Y	13	9
Zr	11	10
Th	38	36
U	22	16
LE	54.7%	1.3
El	PPM	+/- 3σ
Mg	ND	<60000
Al	ND	<10000
Ti	ND	<1200
V	ND	<120
Co	ND	<93
Cu	ND	<56
As	ND	<17
Se	ND	<8
Nb	ND	<24
Mo	ND	<31
Ag	ND	<95
Cd	ND	<99
Sn	ND	<140
Sb	ND	<160
W	ND	<77
Hg	ND	<49

....

Spectrum

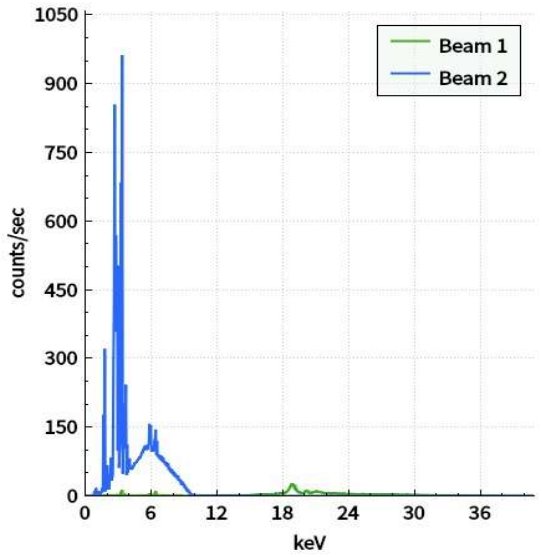

Aiming Image

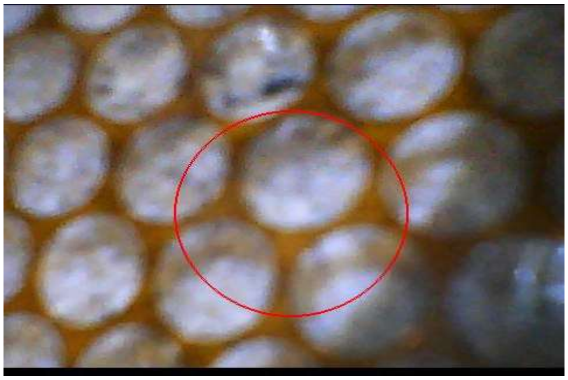

Notes

info: u2

Date:

Signature:

Time : 2024-04-10 12:28:36

Serial Number : 803368

Method : Geochem(2)
Daily ID : 3

Elapsed Time : 30 s

Chemistry

Elapsed time: 30.0s

El	PPM	+/- 3σ
Si	26.77%	0.86
P	2.15%	0.15
S	1.257%	0.092
K	11.49%	0.35
Ca	1.671%	0.086
Cr	810	260
Mn	2510	370
Fe	3010	330
Ni	88	53
Zn	105	32
Sr	17	7
Y	11	8
Cd	54	47
U	28	16
LE	56.0%	1.3
El	PPM	+/- 3σ
Mg	ND	<60000
Al	ND	<11000
Ti	ND	<1200
V	ND	<95
Co	ND	<84
Cu	ND	<56
As	ND	<15
Se	ND	<8
Rb	ND	<12
Zr	ND	<21
Nb	ND	<23
Mo	ND	<30
Ag	ND	<90
Sn	ND	<130
Sb	ND	<160
W	ND	<81
Hg	ND	<48
Pb	ND	<25

....

Spectrum

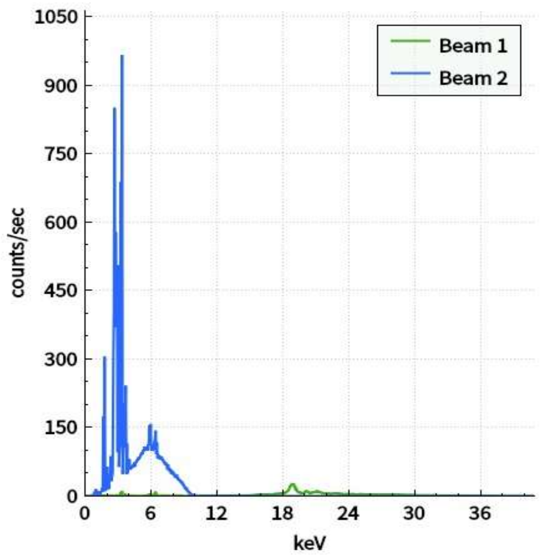

Aiming Image

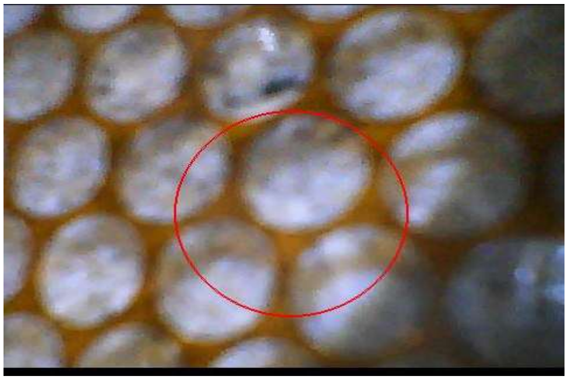

Notes

info: u2

Date: ____________________

Signature: ____________________

Time : 2024-04-10 12:30:33

Serial Number : 803368

Method : Geochem(2)
Daily ID : 4

Elapsed Time : 30 s

Chemistry

Elapsed time: 30.0s

El	PPM	+/- 3σ
Si	61.3%	1.3
P	1600	1600
S	1.80%	0.15
Ca	790	570
Cr	980	310
Fe	3730	380
Ni	139	66
Sr	14	7
Y	11	9
Zr	23	12
Th	39	38
U	23	17
LE	36.1%	1.3
El	PPM	+/- 3σ
Mg	ND	<69000
Al	ND	<7700
K	ND	<1600
Ti	ND	<1200
V	ND	<120
Mn	ND	<2800
Co	ND	<100
Cu	ND	<62
Zn	ND	<29
As	ND	<18
Se	ND	<7
Rb	ND	<13
Nb	ND	<25
Mo	ND	<34
Ag	ND	<100
Cd	ND	<100
Sn	ND	<140
Sb	ND	<170
W	ND	<79
Hg	ND	<44

....

Spectrum

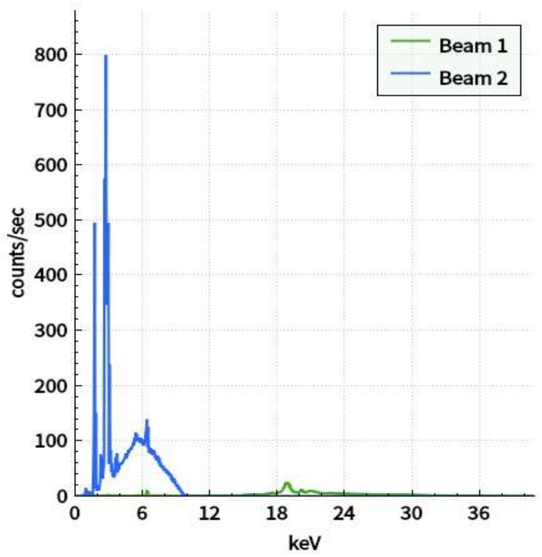

Aiming Image

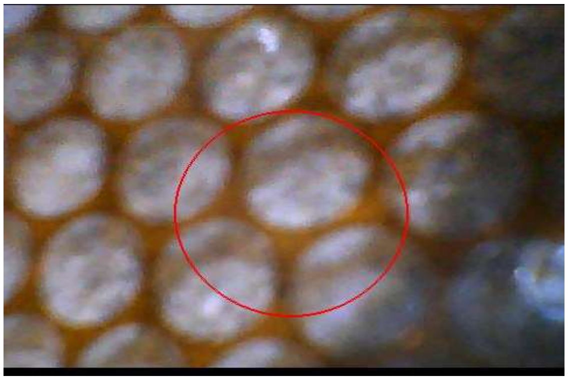

Notes

info: u2

Date: ____________________

Signature: ____________________

Time : 2024-04-10 12:31:08

Serial Number : 803368

Method : Geochem(2)
Daily ID : 5

Elapsed Time : 30 s

Chemistry

Elapsed time: 30.0s

El	PPM	+/- 3σ
Si	61.0%	1.3
P	2000	1600
S	1.80%	0.15
Ca	1050	580
Cr	960	310
Mn	180	170
Fe	3790	380
Ni	120	63
Rb	7	7
Sr	15	7
Y	18	9
Zr	17	11
U	20	17
LE	36.4%	1.3
El	PPM	+/- 3σ
Mg	ND	<66000
Al	ND	<8100
K	ND	<1600
Ti	ND	<1300
V	ND	<120
Co	ND	<95
Cu	ND	<63
Zn	ND	<28
As	ND	<20
Se	ND	<9
Nb	ND	<26
Mo	ND	<33
Ag	ND	<100
Cd	ND	<110
Sn	ND	<140
Sb	ND	<180
W	ND	<67
Hg	ND	<50
Pb	ND	<32

....

Spectrum

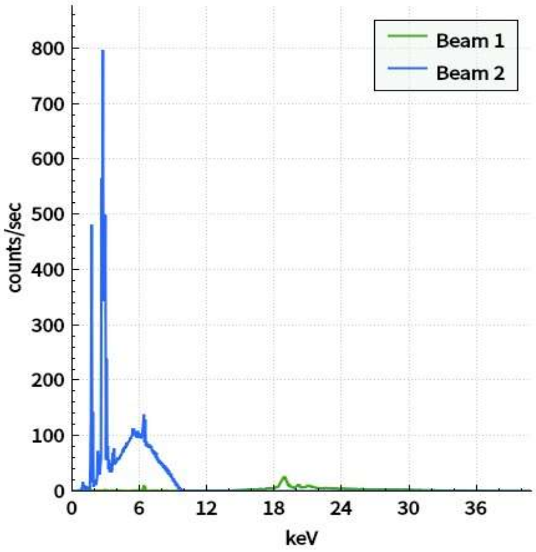

Aiming Image

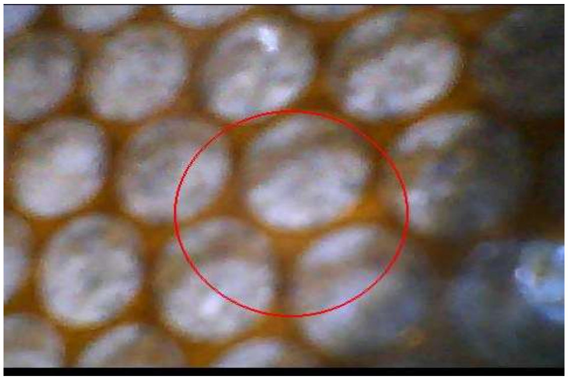

Notes

info: u2

Date: ______________________

Signature: ______________________

Time : 2024-04-10 12:31:46

Serial Number : 803368

Method : Geochem(2)
Daily ID : 6

Elapsed Time : 30 s

Chemistry

Elapsed time: 30.0s

El	PPM	+/- 3σ
Si	60.2%	1.2
S	1.82%	0.14
Ca	660	550
Cr	900	300
Mn	210	180
Fe	3650	370
Ni	136	64
Sr	11	7
Y	17	9
Zr	13	11
Th	49	38
LE	37.4%	1.3
El	PPM	+/- 3σ
Mg	ND	<68000
Al	ND	<7700
P	ND	<1300
K	ND	<1600
Ti	ND	<1200
V	ND	<110
Co	ND	<100
Cu	ND	<64
Zn	ND	<28
As	ND	<18
Se	ND	<8
Rb	ND	<12
Nb	ND	<25
Mo	ND	<32
Ag	ND	<97
Cd	ND	<100
Sn	ND	<140
Sb	ND	<170
W	ND	<80
Hg	ND	<47
Pb	ND	<27

....

Spectrum

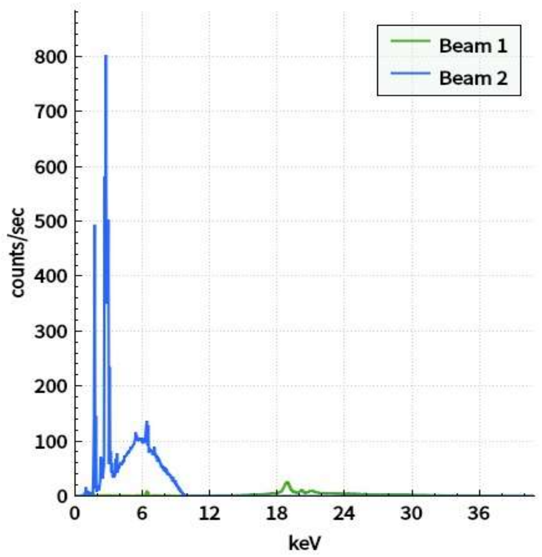

Aiming Image

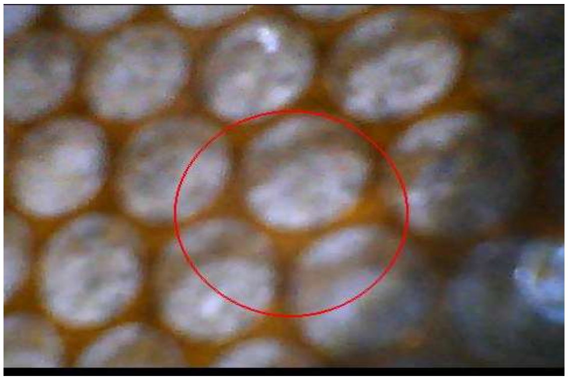

Notes

info: u2

Date: ____________________

Signature: ____________________

Time : 2024-04-09 13:49:00

Serial Number : 803368

Method : Geochem(2)
Daily ID : 1

Elapsed Time : 30 s

Spectrum

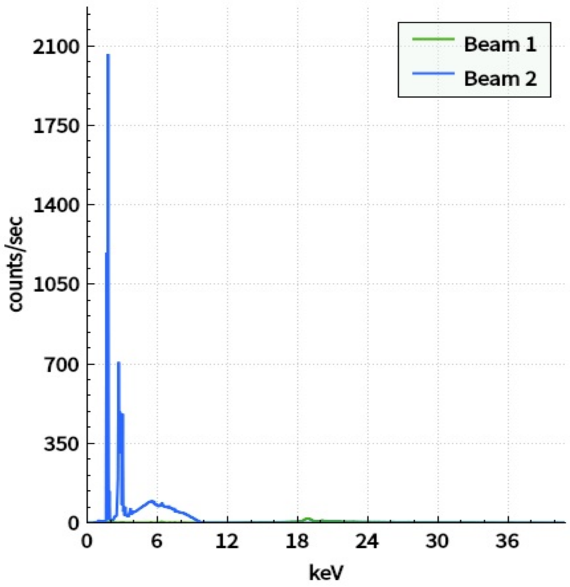

Aiming Image

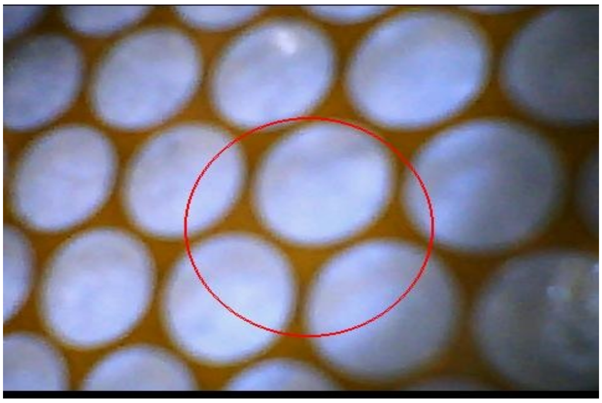

Chemistry

Elapsed time: 30.0s

El	PPM	+/- 3σ
Si	99.771%	0.028
Cr	620	200
Fe	1530	190
Ni	67	38
Sr	5	4
Y	9	5
Zr	10	7
Mo	13	10
Th	32	23
U	13	10
El	PPM	+/- 3σ
Mg	ND	<16000
Al	ND	<2300
P	ND	<300
S	ND	<280
K	ND	<350
Ca	ND	<310
Ti	ND	<310
V	ND	<150
Mn	ND	<800
Co	ND	<110
Cu	ND	<69
Zn	ND	<37
As	ND	<24
Se	ND	<13
Rb	ND	<16
Nb	ND	<32
Ag	ND	<130
Cd	ND	<130
Sn	ND	<180
Sb	ND	<220
W	ND	<110
Hg	ND	<72
Pb	ND	<35

....

Notes

info: u2

Date:

Signature:

Time : 2024-04-09 13:49:43

Serial Number : 803368

Method : Geochem(2)
Daily ID : 2

Elapsed Time : 30 s

Spectrum

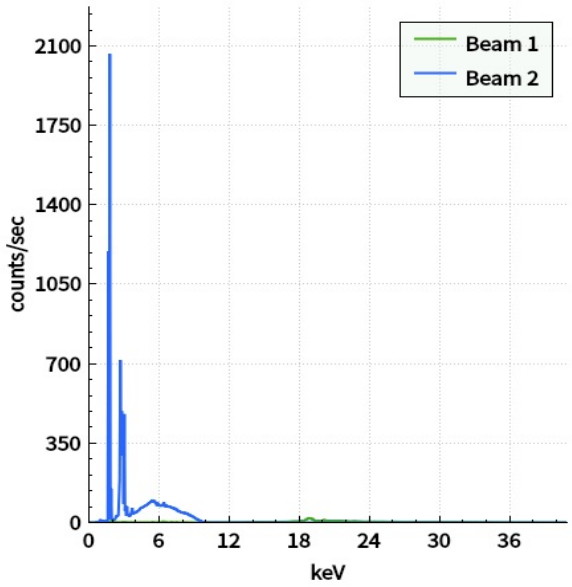

Chemistry

Elapsed time: 30.0s

El	PPM	+/- 3σ
Si	99.753%	0.031
Cr	650	200
Mn	120	110
Fe	1540	190
Ni	58	37
Sr	6	4
Y	12	6
Zr	12	7
Nb	12	8
Mo	15	10
Th	35	23
U	17	11
El	PPM	+/- 3σ
Mg	ND	<16000
Al	ND	<2400
P	ND	<300
S	ND	<280
K	ND	<350
Ca	ND	<310
Ti	ND	<320
V	ND	<170
Co	ND	<120
Cu	ND	<76
Zn	ND	<34
As	ND	<24
Se	ND	<13
Rb	ND	<16
Ag	ND	<130
Cd	ND	<140
Sn	ND	<180
Sb	ND	<230
W	ND	<110
Hg	ND	<63
Pb	ND	<37

....

Aiming Image

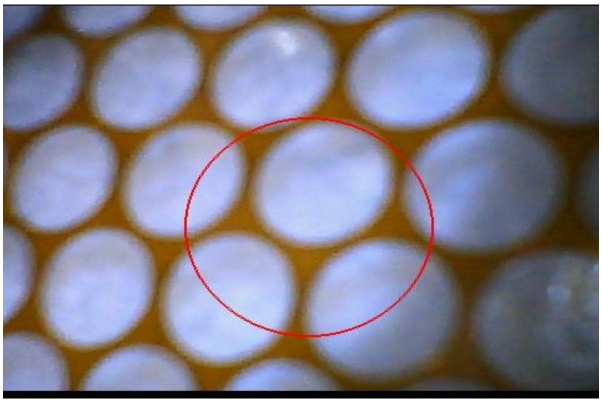

Notes

info: u2

Date: ____________________

Signature: ____________________

Time : 2024-04-09 13:50:19

Serial Number : 803368

Method : Geochem(2)

Daily ID : 3

Elapsed Time : 30 s

Spectrum

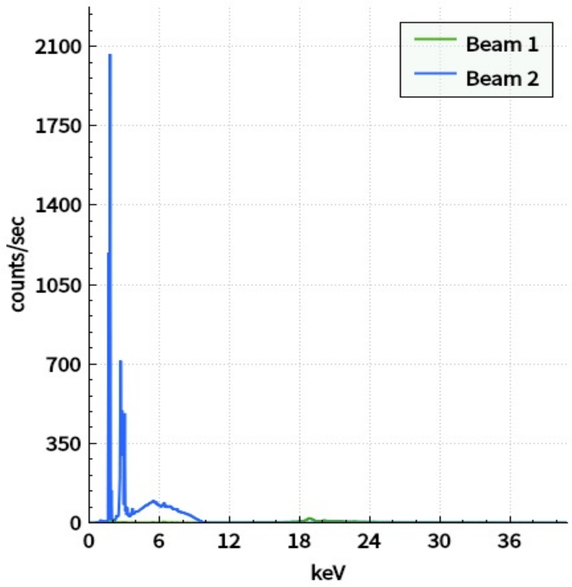

Chemistry

Elapsed time: 30.0s

El	PPM	+/- 3σ
Si	99.776%	0.030
Cr	680	210
Mn	120	110
Fe	1370	180
Sr	9	4
Y	7	5
Zr	11	7
Nb	8	7
Mo	17	10
U	16	10
El	PPM	+/- 3σ
Mg	ND	<16000
Al	ND	<2400
P	ND	<300
S	ND	<270
K	ND	<350
Ca	ND	<310
Ti	ND	<320
V	ND	<170
Co	ND	<110
Ni	ND	<110
Cu	ND	<66
Zn	ND	<36
As	ND	<24
Se	ND	<13
Rb	ND	<17
Ag	ND	<130
Cd	ND	<130
Sn	ND	<180
Sb	ND	<220
W	ND	<99
Hg	ND	<69
Pb	ND	<37
Bi	ND	<110

....

Aiming Image

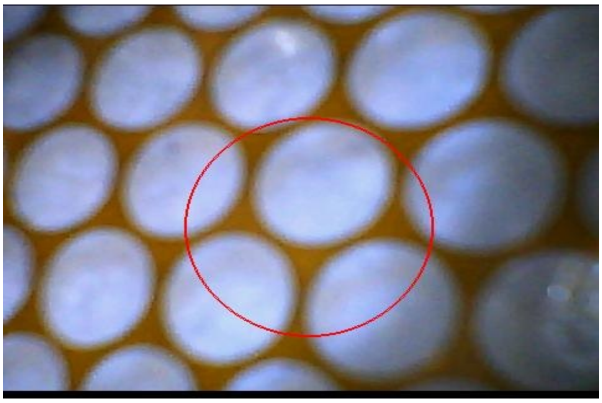

Notes

info: u2

Date: ______________________

Signature: ______________________

Time : 2024-04-09 13:52:33

Serial Number : 803368

Method : Geochem(2)
Daily ID : 4

Elapsed Time : 30 s

Chemistry

Elapsed time: 30.0s

El	PPM	+/- 3σ
Si	99.411%	0.045
Cr	760	240
Mn	290	160
Fe	3200	310
Ni	1470	140
Cu	45	43
Sr	8	5
Y	8	6
Zr	11	8
Nb	11	9
Mo	27	12
Ag	40	33
Th	27	26
El	PPM	+/- 3σ
Mg	ND	<19000
Al	ND	<2900
P	ND	<430
S	ND	<340
K	ND	<450
Ca	ND	<400
Ti	ND	<390
V	ND	<190
Co	ND	<190
Zn	ND	<61
As	ND	<30
Se	ND	<14
Rb	ND	<18
Cd	ND	<160
Sn	ND	<220
Sb	ND	<260
W	ND	<130
Hg	ND	<78
Pb	ND	<49
Bi	ND	<130

....

Spectrum

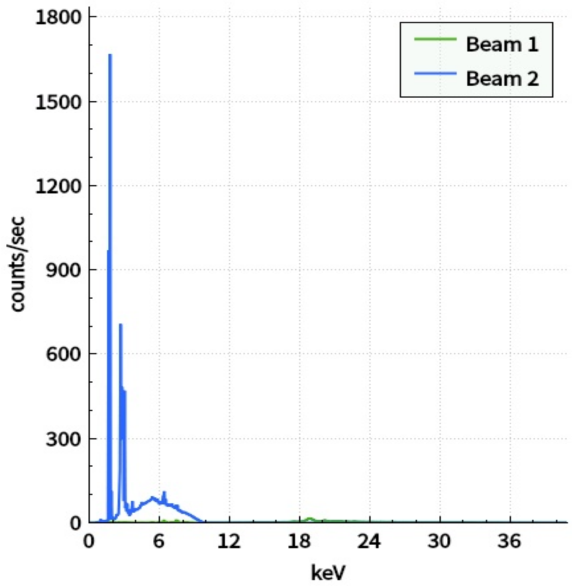

Aiming Image

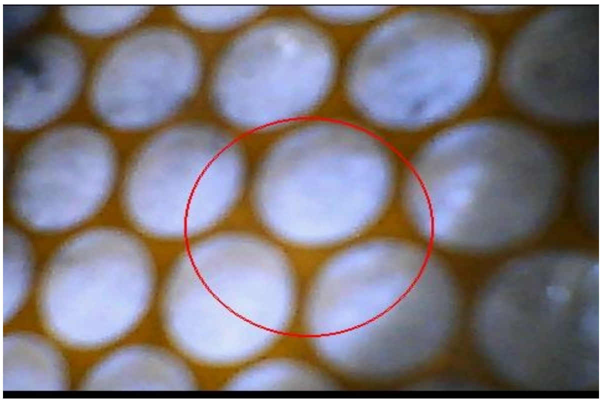

Notes

info: u2

Date:

Signature:

Time : 2024-04-09 13:53:30

Serial Number : 803368

Method : Geochem(2)
Daily ID : 5

Elapsed Time : 30 s

Chemistry

Elapsed time: 30.0s

El	PPM	+/- 3σ
Si	99.392%	0.046
Cr	730	240
Mn	390	170
Fe	3340	320
Ni	1510	140
Zn	21	17
Sr	8	5
Zr	10	8
Nb	11	9
Mo	24	12
Th	31	26
El	PPM	+/- 3σ
Mg	ND	<20000
Al	ND	<3000
P	ND	<430
S	ND	<350
K	ND	<460
Ca	ND	<400
Ti	ND	<390
V	ND	<240
Co	ND	<200
Cu	ND	<150
As	ND	<29
Se	ND	<15
Rb	ND	<18
Y	ND	<27
Ag	ND	<150
Cd	ND	<160
Sn	ND	<220
Sb	ND	<270
W	ND	<130
Hg	ND	<83
Pb	ND	<44
Bi	ND	<130

....

Spectrum

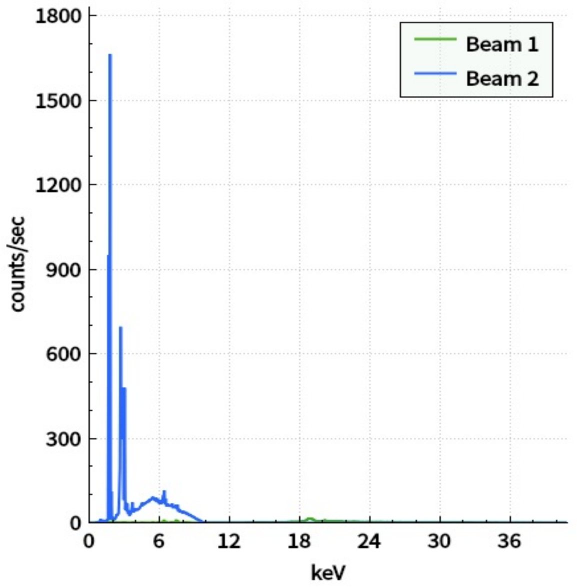

Aiming Image

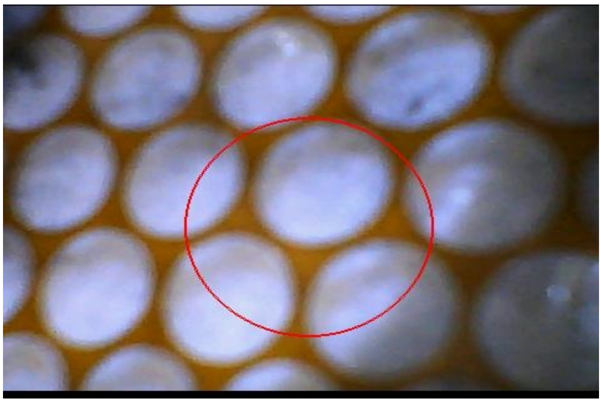

Notes

info: u2

Date:

Signature:

Time : 2024-04-09 13:54:11

Serial Number : 803368

Method : Geochem(2)
Daily ID : 6

Elapsed Time : 30 s

Chemistry

Elapsed time: 30.0s

El	PPM	+/- 3σ
Si	99.419%	0.045
Cr	690	230
Mn	410	170
Fe	3070	300
Ni	1440	140
Cu	60	45
Sr	7	5
Y	10	6
Zr	9	8
Nb	12	9
Mo	23	11
W	39	38
Th	34	26
El	PPM	+/- 3σ
Mg	ND	<20000
Al	ND	<2800
P	ND	<420
S	ND	<340
K	ND	<460
Ca	ND	<400
Ti	ND	<390
V	ND	<220
Co	ND	<190
Zn	ND	<56
As	ND	<28
Se	ND	<14
Rb	ND	<18
Ag	ND	<150
Cd	ND	<160
Sn	ND	<220
Sb	ND	<270
Hg	ND	<84
Pb	ND	<44
Bi	ND	<130

....

Spectrum

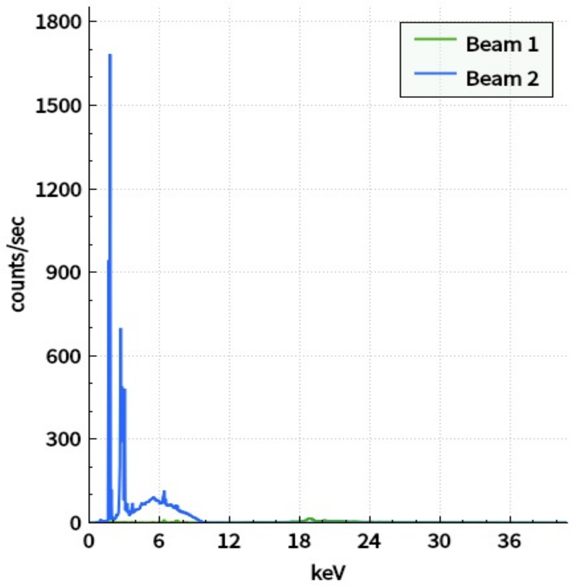

Aiming Image

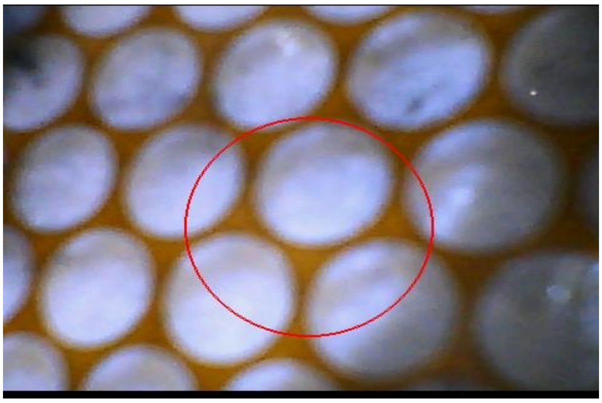

Notes

info: u2

Date:

Signature:

Time : 2024-04-09 13:54:47

Serial Number : 803368

Method : Geochem(2)
Daily ID : 7

Elapsed Time : 30 s

Chemistry

Elapsed time: 30.0s

El	PPM	+/- 3σ
Si	99.408%	0.045
Cr	740	240
Mn	340	160
Fe	3160	310
Ni	1520	140
Cu	47	44
Zn	17	17
Sr	6	5
Y	8	6
Zr	9	8
Nb	12	9
Mo	21	11
Th	36	26
U	13	11
El	PPM	+/- 3σ
Mg	ND	<21000
Al	ND	<2900
P	ND	<430
S	ND	<350
K	ND	<450
Ca	ND	<400
Ti	ND	<390
V	ND	<220
Co	ND	<190
As	ND	<31
Se	ND	<16
Rb	ND	<19
Ag	ND	<160
Cd	ND	<160
Sn	ND	<220
Sb	ND	<270
W	ND	<150
Hg	ND	<91
Pb	ND	<46

....

Spectrum

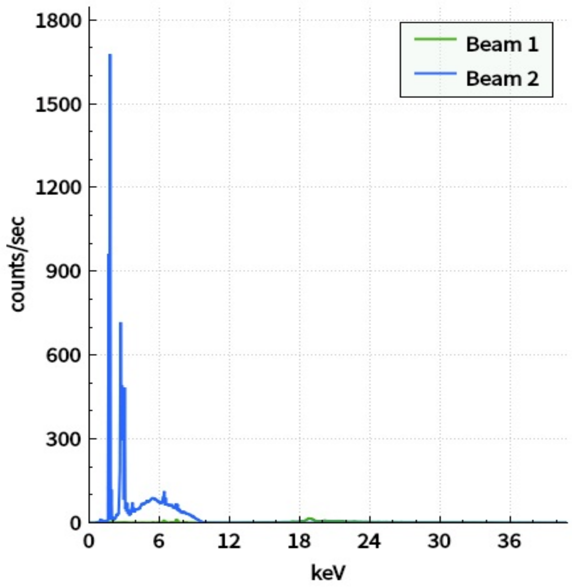

Aiming Image

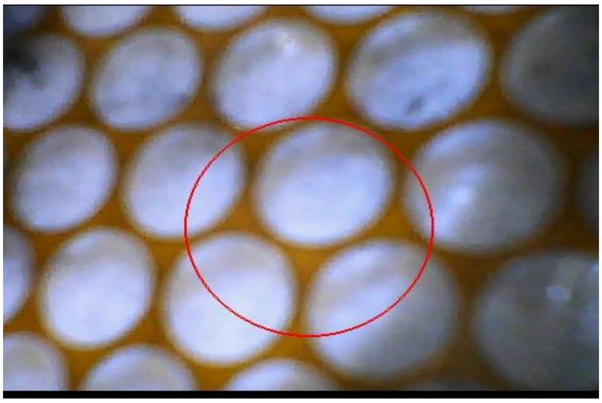

Notes

info: u2

Date:

Signature:

Time : 2024-04-09 13:55:22

Serial Number : 803368

Method : Geochem(2)
Daily ID : 8

Elapsed Time : 30 s

Chemistry

Elapsed time: 30.0s

El	PPM	+/- 3σ
Si	99.421%	0.045
Cr	660	230
Mn	410	170
Fe	3200	310
Ni	1350	130
Cu	61	44
Sr	9	5
Y	8	6
Mo	23	12
Ag	35	33
Th	35	26
El	PPM	+/- 3σ
Mg	ND	<19000
Al	ND	<2900
P	ND	<430
S	ND	<340
K	ND	<460
Ca	ND	<400
Ti	ND	<390
V	ND	<210
Co	ND	<180
Zn	ND	<54
As	ND	<30
Se	ND	<17
Rb	ND	<19
Zr	ND	<33
Nb	ND	<36
Cd	ND	<150
Sn	ND	<210
Sb	ND	<250
W	ND	<130
Hg	ND	<83
Pb	ND	<48
Bi	ND	<130

....

Spectrum

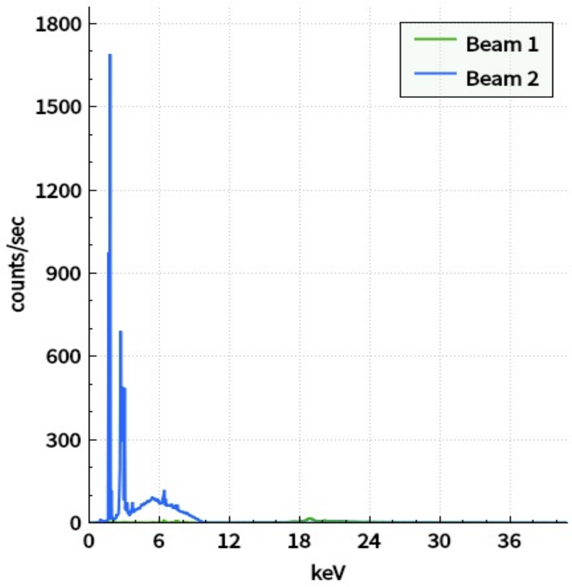

Aiming Image

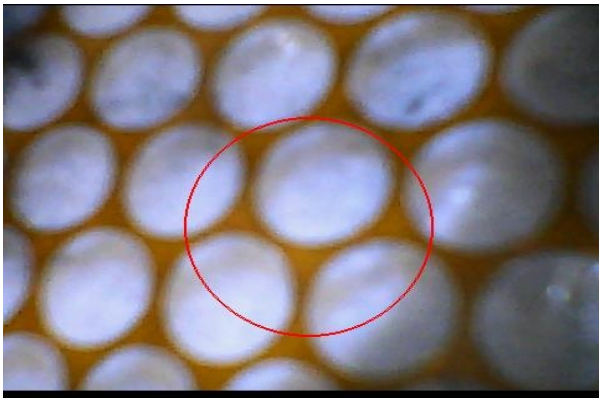

Notes

info: u2

Date:

Signature:
