# Supplementary material for: Mechanistic insights into the plant biostimulant activity of a novel formulation based on rice husk nanobiosilica embedded in a seed coating alginate film
Source: Front Plant Sci. 2024 May 21;15:1349573. doi: 10.3389/fpls.2024.1349573 (PMC11148368; doi:10.3389/fpls.2024.1349573)
Supplement: Supplementary file 10 [file DataSheet_2.docx]

Supplementary Material

# Materials

The following chemicals were used: hydrochloric acid solution 1 N, sodium chloride, glacial acetic acid, isopropyl alcohol, ethyl alcohol, sulfosalicylic acid 30%, potassium iodide (Chimreactiv, Bucharest, Romania), sodium hydroxide, potassium sodium tartrate for analysis, phenol molecular biology grade, maltose monohydrate, starch soluble, di-sodium hydrogen phosphate dihydrate (Na_2_HPO_4_ x 2H_2_O), sodium dihydrogen phosphate monohydrate (NaH_2_PO_4_ x H_2_O), potassium dihydrogen phosphate (KH_2_PO_4_), Triton® X-100, trichloroacetic acid for analysis, L-proline extra pure, bromocresol purple, hydrogen peroxide solution 30% w/w, agar, glycerol 99.5%, dimethyl sulfoxide, D-sorbitol, tris(hydroxymethyl)aminomethane (Scharlau, Barcelona, Spain), silica fumed 99.8%, 1,1,3,3-tetramethoxypropane 99%, 2-thiobarbituric acid ≥98%, thiazolyl blue tetrazolium bromide, guaiacol, sodium hypochlorite, 3,3′-diaminobenzidine, ethylenediaminetetraacetic acid tetrasodium salt dihydrate, Silicon ICP Standard traceable to SRM from NIST SiO_2_ in NaOH 2% 1000 mg/L Si Certipur (Sigma-Aldrich, Darmstadt, Germany), Certified reference material 21 components 100 mg/L each in HNO_3_ 5% (CPAchem, Bogomilovo, Bulgaria) for ICP-OES, 3,5-dinitrosalicylic acid 97+%, riboflavin 98%, nitro blue tetrazolium chloride (NBT) 98+%, magnesium chloride anhydrous 99%, L-glutathione oxidized (Alfa Aesar, Massachusetts, USA), sodium alginate (BioChemica, PanReac AppliChem ITW Reagents, Monza, Italy), L-methionine 98+% (Acros Organics, New Jersey, USA), toluene (Chimopar, Bucharest, Romania), methanol (Honeywell, Charlotte, NC, USA), 2,7-dichlorodihydrofluorescein, nicotinamide adenine dinucleotide phosphate (NADPH) (Cayman Chemicals, Michigan, USA), Griess reagent system (Promega Corporation, Madison, WI, USA), 2,5-Bis(5-tert-butyl-2-benzo-oxazol-2-yl, cystine (ThermoFisher Scientific, Bremen, Germany).

# Supplementary Definitions

BET – Brunauer-Emmett-Teller method is the most known gas adsorption model, which extends the Langmuir mono-layer adsorption to multi-layer adsorption and gives the most relevant results in the range 0.05-0.3 of p/p^0^, meaning for micropores and mesopores.

DFT – Density Functional Theory, with its main type (NL)DFT – (Non-Linear)Density Functional Theory is a general method for the full p/p^0^ range (0.001-1) that evaluates the adsorption phenomena by statistical mechanics using a mathematical model that considers both gas-solid and gas-gas interactions, and also pore geometry.

BJH - Barrett-Joyner-Halenda method is a modified Kelvin equation that correlates the pressure at which the adsorbate condenses in multi-layers of certain thickness, being more specific for mesopores and macropores, respectively p/p^0^ > 0.3, where multi-layers of adsorbed molecules usually occur.

T-plot or t-method offers information regarding the specific surface area and volumes of micro- and mesopores and the thickness of the multi-layer as a function of increasing relative pressure p/p^0^.

DR - Dubinin-Radushkevic is a method specific for the adsorption phenomena in micropores (< 2 nm, p/p^0^ < 0.1) based on average adsorption energy and pore size distribution.

# Supplementary Figures and Tables

## Supplementary Figures

**Supplementary Figure S1.** Details in the 3800-3600 cm^−1^ region: RH – native rice husk; hRH – hydrolyzed rice husk; cRH – calcined rice husk; fSiO2 – commercial fumed silica.

**Supplementary Figure S2.** SEM analysis: (A) Rice husk (RH); (B) Hydrolyzed rice husk (hRH), and (C) Calcined rice husk (cRH).

**Supplementary Figure S3.** Mung bean seedling growth in the absence of salt stress; C – Control (uncoated mung bean seeds), no salt stress; V0 – AGS solution seed coating, no salt stress; V1 – AGS solution+1% SiNPs seed coating, no salt stress; V2 – AGS solution+2% SiNPs seed coating, no salt stress; V3 – AGS solution+3% SiNPs seed coating, no salt stress; V4 – AGS solution+4% SiNPs seed coating, no salt stress.

**Supplementary Figure S4.** Mung bean seedling growth in the presence of salt stress; CS – Control (uncoated mung bean seeds), salt stress; V0S – AGS solution seed coating, salt stress; V1S – AGS solution+1% SiNPs seed coating, salt stress; V2S –AGS solution+2% SiNPs seed coating, salt stress; V3S – AGS solution+3% SiNPs seed coating, salt stress; V4S – AGS solution+4%SiNPs seed coating, salt stress.

**Supplementary Figure S5.** Rhizosphere (medium) acidification; C – Control (uncoated mung bean seeds), no salt stress; V0 – AGS solution seed coating, no salt stress; V1 – AGS solution+1% SiNPs seed coating, no salt stress; V2 – AGS solution+2% SiNPs seed coating, no salt stress; V3 – AGS solution+3% SiNPs seed coating, no salt stress; V4 – AGS solution+4% SiNPs seed coating, no salt stress; CS – Control, salt stress (uncoated mung bean seeds); V0S – AGS solution seed coating, salt stress; V1S – AGS solution+1% SiNPs seed coating, salt stress; V2S –AGS solution+2% SiNPs seed coating, salt stress; V3S – AGS solution+3% SiNPs seed coating, salt stress; V4S – AGS solution+4%SiNPs seed coating, salt stress.

**Supplementary Figure S6.** Extracellular H^+^ level; C – Control (uncoated mung bean seeds), no salt stress; V0 – AGS solution seed coating, no salt stress; V1 – AGS solution+1% SiNPs seed coating, no salt stress; V2 – AGS solution+2% SiNPs seed coating, no salt stress; V3 – AGS solution+3% SiNPs seed coating, no salt stress; V4 – AGS solution+4% SiNPs seed coating, no salt stress; CS – Control, salt stress (uncoated mung bean seeds); V0S – AGS solution seed coating, salt stress; V1S – AGS solution+1% SiNPs seed coating, salt stress; V2S –AGS solution+2% SiNPs seed coating, salt stress; V3S – AGS solution+3% SiNPs seed coating, salt stress; V4S – AGS solution+4% SiNPs seed coating, salt stress.

**Supplementary Figure S7.** H_2_O_2_ detection in mung bean leaves using DAB staining; C – Control (uncoated mung bean seeds), no salt stress; V0 – AGS solution seed coating, no salt stress; V1 – AGS solution+1% SiNPs seed coating, no salt stress; V2 – AGS solution+2% SiNPs seed coating, no salt stress; V3 – AGS solution+3% SiNPs seed coating, no salt stress; V4 – AGS solution+4% SiNPs seed coating, no salt stress; CS – Control, salt stress (uncoated mung bean seeds); V0S – AGS solution seed coating, salt stress; V1S – AGS solution+1% SiNPs seed coating, salt stress; V2S –AGS solution+2% SiNPs seed coating, salt stress; V3S – AGS solution+3% SiNPs seed coating, salt stress; V4S – AGS solution+4%SiNPs seed coating, salt stress.

**Supplementary Figure S8.** Fluorescence microscopy observations of H_2_DCFDA-mediated fluorescence; C – Control (uncoated mung bean seeds), no salt stress; V0 – AGS solution seed coating, no salt stress; V1 – AGS solution+1% SiNPs seed coating, no salt stress; V2 – AGS solution+2% SiNPs seed coating, no salt stress; V3 – AGS solution+3% SiNPs seed coating, no salt stress; V4 – AGS solution+4% SiNPs seed coating, no salt stress; CS – Control, salt stress (uncoated mung bean seeds); V0S – AGS solution seed coating, salt stress; V1S – AGS solution+1% SiNPs seed coating, salt stress; V2S –AGS solution+2% SiNPs seed coating, salt stress; V3S – AGS solution+3% SiNPs seed coating, salt stress; V4S – AGS solution+4%SiNPs seed coating,

## Supplementary Tables

**Supplementary Table S1.** Young’s modulus and the ultimate tensile strength (UTS) for each film prepared using mixture design.

| Sample code | Commercial sodium alginate (mg)  (X_1_) | Glycerol (mg)  (X_2_) | Sorbitol (mg)  (X_3_) | Modulus [MPa]  (Y_1_) | UTS [MPa]  (Y_2_) |
| --- | --- | --- | --- | --- | --- |
| 1 | 800 [1] | 250 [0] | 250 [0] | 2.6941 | 5.8815 |
| 2 | 400 [0] | 650 [1] | 250 [0] | 0.0220 | 1.1967 |
| 3 | 400 [0] | 250 [0] | 650 [1] | 0.0245 | 0.7913 |
| 4 | 600 [0.5] | 450 [0.5] | 250 [0] | 0.1107 | 3.5182 |
| 5 | 600 [0.5] | 250 [0] | 450 [0.5] | 0.0870 | 2.7758 |
| 6 | 400 [0] | 450 [0.5] | 450 [0.5] | 0.0161 | 0.3965 |
| 7a | 533.3 [0.33] | 383.3 [0.33] | 383.3 [0.33] | 0.0332 | 0.8362 |
| 7b | 533.3 [0.33] | 383.3 [0.33] | 383.3 [0.33] | 0.0328 | 1.3198 |

**Supplementary Table S2.** Specific XRD peaks.

| fSiO_2_ | RH | hRH | cRH | CelluIα | CelluIβ | CelluAm. |
| --- | --- | --- | --- | --- | --- | --- |
| - | - | - | - | 10.28 | - | - |
| - | - | - | - | 14.26 | 14.83 | - |
| - | 15.2 | 15.8 | - | - | - | 15.28 |
| - | - | - | - | 16.77 | 16.42 | - |
| - | 17.2 | - | - | - | 17.09 | - |
| - | 18.1 | - | - | - | 18.52 | - |
| - | - | - | - | - | 20.21 | 19.78 |
| 21.9 | 22.2 | 22.2 | 22.0 | 21.80 | 22.71 | - |
| - | - | - | - | 23.24 | - | - |
| - | 26.7 | 26.7 | - | 25.04 | - | 27.13 |
| - | - | - | - | - | - | - |
| - | - | - | - | - | - | - |
| - | 34.6 | 34.6 | - | - | 34.64 | - |
| - | - | - | - | - | - | 36.1 |

fSiO2 – commercial fumed silica; RH – native rice husk; hRH – hydrolyzed rice husk; cRH – calcined rice husk; Cellu – cellulose; CelluAm – Amorphous cellulose

**Supplementary Table S3.** BET analysis

| Method | SSA,  m^2^/g | CPV,  cm^3^/g | < 2PV,  cm^3^/g (%v/v) | 2-12PV,  cm^3^/g (%v/v) | 12-40PV,  cm^3^/g (%v/v) | D,* nm |
| --- | --- | --- | --- | --- | --- | --- |
| BET(ads)  (TPV@STP) | 240.0 | 226.6 | 74.0(32.7%) | 108.6(47.9%) | 31.3(13.8%) | 8.8 |
| BET(des)  (CPV) | - | 0.347 | 0.111(32.0%) | 0.191(55.0%) | 0.037(10.7%) | 8.9 |
| NL-DFT | 242.4 | 0.335 | 0.006(1.8%) | 0.276(82.4%) | 0.045(13.4%) | 5.1 |
| BJH** | 421.2 | 0.383 | 0.068(17.8%) | 0.256(66.8%) | 0.047(12.3%) | 3.5** |
| BJH(ads) | 502.6 | 0.401 | 0.081(21.4%) | 0.252(66.7%) | 0.045(11.9%) | 3.2 |
| BJH(des) | 339.9 | 0.366 | 0.054(14.9%) | 0.260(71.8%) | 0.048(13.3%) | 3.8 |

* D- computed average mesopore size between 0.35-0.93 p/p^0^; ** mean values for adsorption-desorption; SSA- Specific surface area, TPV-Total pore volume at standard temperature and pressure (STP); CPV-Cumulative pore volume for liquid N_2_; < 2PV = total pore volume for pores < 2nm; 2-12PV = total pore volume for pores 2-12nm; 12-40PV = total pore volume for pores 12-40nm.

**Supplementary Table S4.** SEM - EDX analysis

| Sample | Element | Weight % | Atomic % | Error % |
| --- | --- | --- | --- | --- |
| RH | C K | 44.07 | 52.08 | 8.13 |
|  | N K | 3.98 | 4.03 | 28.53 |
|  | O K | 46.54 | 41.29 | 11.2 |
|  | Mg K | 0.46 | 0.27 | 25.41 |
|  | Al K | 0.22 | 0.11 | 34 |
|  | Si K | 3.19 | 1.61 | 6.95 |
|  | P K | 0.29 | 0.13 | 29.17 |
|  | S K | 0.13 | 0.06 | 52.95 |
|  | Cl K | 0.28 | 0.11 | 28.57 |
|  | K K | 0.84 | 0.3 | 9.95 |
| hRH | C K | 58.17 | 65.55 | 6.63 |
|  | O K | 39.28 | 33.22 | 11.33 |
|  | Al K | 0.28 | 0.14 | 23.7 |
|  | Si K | 2.13 | 1.03 | 6.6 |
|  | S K | 0.14 | 0.06 | 30.15 |
| cRH | C K | 16.72 | 23.93 | 11.99 |
|  | O K | 54.18 | 58.23 | 9.16 |
|  | Al K | 0.76 | 0.48 | 10.66 |
|  | Si K | 28.35 | 17.35 | 4.3 |

RH – native rice husk; hRH – hydrolyzed rice husk; cRH – calcined rice husk;

**Supplementary Table S5.** ANOVA analysis of Young’s modulus.

| Source | Sum of  Squares | df | Mean | F-value | p-value |
| --- | --- | --- | --- | --- | --- |
| Model | 18.98 | 4 | 4.75 | 59.05 | 0.003 |
| Linear Mixture | 17.07 | 2 | 8.54 | 106.21 | 0.002 |
| AB | 0.68 | 1 | 0.68 | 8.49 | 0.062 |
| AC | 1.19 | 1 | 1.19 | 14.84 | 0.031 |
| Residual | 0.24 | 3 | 0.08 |  |  |
| Lack of Fit | 0.24 | 2 | 0.12 | 1640.48 | 0.017 |
| Pure Error | 0.00 | 1 | 0.00 |  |  |
| Cor Total | 19.22 | 7 |  |  |  |

**Supplementary Table S6.** ANOVA analysis of UTS.

| Source | Sum of Squares | df | Mean Square | F-value | p-value |
| --- | --- | --- | --- | --- | --- |
| Model | 21.16 | 2 | 10.58 | 16.31 | 0.0064 |
| ⁽¹⁾Linear Mixture | 21.16 | 2 | 10.58 | 16.31 | 0.0064 |
| Residual | 3.24 | 5 | 0.6487 |  |  |
| Lack of Fit | 3.13 | 4 | 0.7817 | 6.68 | 0.2814 |
| Pure Error | 0.1169 | 1 | 0.1169 |  |  |
| Cor Total | 24.40 | 7 |  |  |  |

**Supplementary Table S7.** Water activity (aW) of mung bean seeds with different coatings (± standard error).

|  | Mung bean seeds | | | | | | |
| --- | --- | --- | --- | --- | --- | --- | --- |
|  | **Untreated** | **C** | **V0** | **V1** | **V2** | **V3** | **V4** |
| aW | 0.446±0.003 | 0.461±0.001 | 0.469±0.001 | 0.461±0.001 | 0.460±0.008 | 0.463±0.010 | 0.468±0.002 |

C – Control (uncoated mung bean seeds); V0 – AGS solution seed coating; V1 – AGS solution+1% SiNPs seed coating; V2 – AGS solution+2% SiNPs seed coating; V3 – AGS solution+3% SiNPs seed coating; V4 – AGS solution+4% SiNPs seed coating.

**Supplementary Table S8.** Pearson correlation for the salt-free set.

|  | **Root**  **length** | **Stem length** | **Seedling height** | **Alfa-amylase activity** | **Ch a** | **Ch b** | **Total ch**  **content** | **Caro-tenoids** | **MTT** | **eH^+^** | **seH^+^** | **L-Pro** | **MDA** | **H_2_O_2_** | **SOD activity** | **G-POX activity** | **GR activity** | **NO** | **Leaf ROS** | **Total ROS** |  |
| --- | --- | --- | --- | --- | --- | --- | --- | --- | --- | --- | --- | --- | --- | --- | --- | --- | --- | --- | --- | --- | --- |
| **Root**  **length** | 1 |  |  |  |  |  |  |  |  |  |  |  |  |  |  |  |  |  |  |  |  |
| **Stem**  **length** | 0.889  ** | 1 |  |  |  |  |  |  |  |  |  |  |  |  |  |  |  |  |  |  |  |
| **Seedling height** | 0.989  ** | 0.947  ** | 1 |  |  |  |  |  |  |  |  |  |  |  |  |  |  |  |  |  |  |
| **Alfa-**  **amylase activity** | -0.226 | 0.164 | -0.107 | 1 |  |  |  |  |  |  |  |  |  |  |  |  |  |  |  |  |  |
| **Ch a** | 0.392 | 0.337 | 0.384 | -0.157 | 1 |  |  |  |  |  |  |  |  |  |  |  |  |  |  |  |  |
| **Ch b** | 0.127 | 0.085 | 0.117 | -0.217 | 0.847  ** | 1 |  |  |  |  |  |  |  |  |  |  |  |  |  |  |  |
| **Total ch**  **content** | 0.291 | 0.239 | 0.281 | -0.189 | 0.972  ** | 0.948  ** | 1 |  |  |  |  |  |  |  |  |  |  |  |  |  |  |
| **Caro-**  **tenoids** | 0.463 | 0.305 | 0.424 | 0.055 | 0.177 | -0.306 | -0.029 | 1 |  |  |  |  |  |  |  |  |  |  |  |  |  |
| **MTT** | -0.358 | -0.342 | -0.362 | -0.328 | 0.514 | 0.722 | 0.626 | -0.597 | 1 |  |  |  |  |  |  |  |  |  |  |  |  |
| **eH^+^** | 0.154 | 0.169 | 0.163 | 0.233 | 0.827  * | 0.771  ms | 0.835  * | 0.190 | 0.287 | 1 |  |  |  |  |  |  |  |  |  |  |  |
| **seH^+^** | -0.381 | -0.573 | -0.452 | -0.497 | 0.467 | 0.735  ms | 0.604 | -0.403 | 0.812  * | 0.415 | 1 |  |  |  |  |  |  |  |  |  |  |
| **L-Pro** | -0.929  ** | -0.785  ms | -0.906  ** | 0.213 | -0.519 | -0.371 | -0.475 | -0.375 | 0.264 | -0.412 | 0.145 | 1 |  |  |  |  |  |  |  |  |  |
| **MDA** | -0.972  ** | -0.803  ms | -0.942  ** | 0.245 | -0.395 | -0.065 | -0.266 | -0.643 | 0.435 | -0.192 | 0.366 | 0.908  * | 1 |  |  |  |  |  |  |  |  |
| **H_2_O_2_** | -0.559 | -0.365 | -0.511 | 0.703 | -0.669 | -0.513 | -0.627 | -0.078 | -0.477 | -0.154 | -0.334 | 0.488 | 0.524 | 1 |  |  |  |  |  |  |  |
| **SOD**  **activity** | 0.227 | -0.196 | 0.097 | -0.775  ms | 0.494 | 0.508 | 0.520 | 0.213 | 0.284 | 0.350 | 0.681 | -0.397 | -0.325 | -0.560 | 1 |  |  |  |  |  |  |
| **G-POX activity** | 0.358 | 0.311 | 0.352 | 0.390 | -0.087 | -0.177 | -0.130 | 0.529 | -0.775  ms | 0.312 | -0.410 | -0.499 | -0.442 | 0.463 | 0.022 | 1 |  |  |  |  |  |
| **GR**  **activity** | -0.616 | -0.579 | -0.619 | 0.335 | -0.861  * | -0.684 | -0.817  * | -0.072 | -0.477 | -0.464 | -0.243 | 0.597 | 0.549 | 0.891  * | -0.354 | 0.305 | 1 |  |  |  |  |
| **NO** | 0.422 | 0.579 | 0.482 | 0.567 | -0.041 | -0.002 | -0.025 | 0.107 | -0.580 | 0.298 | -0.491 | -0.546 | -0.366 | 0.410 | -0.312 | 0.809 | 0.108 | 1 |  |  |  |
| **Leaf ROS** | -0.853  * | -0.760  ms | -0.844  * | 0.166 | -0.788  ms | -0.583 | -0.729  ms | -0.365 | -0.004 | -0.621 | -0.019 | 0.928  ** | 0.834  * | 0.632 | -0.416 | -0.305 | 0.810  ms | -0.383 | 1 |  |  |
| **Total ROS** | -0.88  * | -0.595 | -0.810  ms | 0.527 | -0.271 | 0.041 | -0.145 | -0.629 | 0.377 | 0.029 | 0.252 | 0.775  ms | 0.934  ** | 0.617 | -0.486 | -0.269 | 0.476 | -0.080 | 0.673 | 1 | |

**Supplementary Table S9.** Pearson correlation for the set subjected to salt stress.

|  | **Root**  **length** | **Stem**  **length** | **Seedling**  **height** | **Alfa-**  **amylase**  **activity** | **Ch a** | **Ch b** | **Total ch content** | **Caro-tenoids** | **MTT** | **eH^+^** | **seH^+^** | **L-Pro** | **MDA** | **H_2_O_2_** | **CAT**  **activity** | **SOD**  **activity** | **G-POX**  **activity** | **GR**  **activity** | **NO** | **Leaf ROS** | **Total**  **ROS** |
| --- | --- | --- | --- | --- | --- | --- | --- | --- | --- | --- | --- | --- | --- | --- | --- | --- | --- | --- | --- | --- | --- |
| **Root**  **length** | 1 |  |  |  |  |  |  |  |  |  |  |  |  |  |  |  |  |  |  |  |  |
| **Stem**  **length** | 0.886  * | 1 |  |  |  |  |  |  |  |  |  |  |  |  |  |  |  |  |  |  |  |
| **Seedling**  **height** | 0.983  ** | 0.956  ** | 1 |  |  |  |  |  |  |  |  |  |  |  |  |  |  |  |  |  |  |
| **Alfa-**  **amylase activity** | 0.722 | 0.555 | 0.677 | 1 |  |  |  |  |  |  |  |  |  |  |  |  |  |  |  |  |  |
| **Ch a** | 0.832  * | 0.809  ms | 0.846  * | 0.407 | 1 |  |  |  |  |  |  |  |  |  |  |  |  |  |  |  |  |
| **Ch b** | 0.657 | 0.781  ms | 0.724 | 0.107 | 0.926  ** | 1 |  |  |  |  |  |  |  |  |  |  |  |  |  |  |  |
| **Total ch**  **content** | 0.792  ms | 0.813  * | 0.822  * | 0.321 | 0.993  ** | 0.963  ** | 1 |  |  |  |  |  |  |  |  |  |  |  |  |  |  |
| **Caro-**  **tenoids** | 0.753  ms | 0.686 | 0.748  ms | 0.377 | 0.959  ** | 0.844  * | 0.939  ** | 1 |  |  |  |  |  |  |  |  |  |  |  |  |  |
| **MTT** | 0.052 | 0.079 | 0.064 | -0.254 | 0.415 | 0.542 | 0.460 | 0.283 | 1 |  |  |  |  |  |  |  |  |  |  |  |  |
| **eH^+^** | 0.725 | 0.650 | 0.716 | 0.637 | 0.874  * | 0.703 | 0.835  * | 0.86  * | 0.387 | 1 |  |  |  |  |  |  |  |  |  |  |  |
| **seH^+^** | -0.135 | 0.016 | -0.079 | -0.171 | 0.287 | 0.419 | 0.332 | 0.197 | 0.857  * | 0.437 | 1 |  |  |  |  |  |  |  |  |  |  |
| **L-Pro** | -0.928  ** | -0.871  * | -0.932  ** | -0.684 | -0.799  ms | -0.626 | -0.758  ms | -0.799  ms | 0.192 | -0.685 | 0.270 | 1 |  |  |  |  |  |  |  |  |  |
| **MDA** | -0.901  * | -0.927  ** | -0.937  ** | -0.542 | -0.947  ** | -0.859  * | -0.935  ** | -0.906  * | -0.145 | -0.816  * | -0.077 | 0.929  ** | 1 |  |  |  |  |  |  |  |  |
| **H_2_O_2_** | -0.922  ** | -0.867  * | -0.926  ** | -0.861  * | -0.697 | -0.522 | -0.654 | -0.571 | -0.048 | -0.733  ms | 0.003 | 0.817  * | 0.800  ms | 1 |  |  |  |  |  |  |  |
| **CAT**  **activity** | -0.101 | -0.306 | -0.185 | 0.434 | -0.129 | -0.408 | -0.217 | 0.083 | -0.427 | 0.248 | -0.133 | -0.070 | 0.093 | 0.033 | 1 |  |  |  |  |  |  |
| **SOD**  **activity** | 0.839  * | 0.734  ms | 0.821  * | 0.665 | 0.807  ms | 0.591 | 0.753  ms | 0.876  * | -0.170 | 0.769  ms | -0.188 | -0.955  ** | -0.893  * | -0.709 | 0.301 | 1 |  |  |  |  |  |
| **G-POX**  **activity** | 0.854  * | 0.809  ms | 0.86  * | 0.714 | 0.807  ms | 0.614 | 0.761  ms | 0.846  * | -0.167 | 0.791  ms | -0.134 | -0.965  ** | -0.921  ** | -0.779  ms | 0.261 | 0.985  ** | 1 |  |  |  |  |
| **GR**  **activity** | -0.841  * | -0.946  ** | -0.906  * | -0.400 | -0.825  * | -0.811  ms | -0.834  * | -0.770  ms | 0.020 | -0.577 | 0.119 | 0.909  * | 0.941  ** | 0.712 | 0.279 | -0.806  ms | -0.842  * | 1 |  |  |  |
| **NO** | 0.741  ms | 0.877  * | 0.816  * | 0.565 | 0.691 | 0.622 | 0.681 | 0.689 | -0.260 | 0.602 | -0.154 | -0.902  * | -0.878  * | -0.709 | 0.051 | 0.85  * | 0.913  * | -0.91  * | 1 |  |  |
| **Leaf ROS** | -0.891  * | -0.803  ms | -0.881  * | -0.701 | -0.874  * | -0.673 | -0.826  * | -0.902  * | 0.036 | -0.85  * | 0.059 | 0.957  ** | 0.941  ** | 0.794  ms | -0.224 | -0.985  ** | -0.987  ** | 0.831  * | -0.853  * | 1 |  |
| **Total ROS** | -0.703 | -0.542 | -0.660 | -0.475 | -0.655 | -0.442 | -0.600 | -0.786 | 0.332 | -0.535 | 0.426 | 0.87  * | 0.740  ms | 0.473 | -0.319 | -0.933  ** | -0.869  * | 0.710 | -0.722 | 0.864  * | 1 |


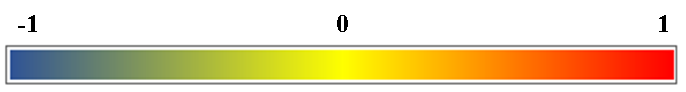


**. Correlation is significant at the 0.01 level.

*. Correlation is significant at the 0.05 level.

ms. Marginally significant; 0.05<σ≤0.1.
