# Supplementary material for: Mechanistic insights into the plant biostimulant activity of a novel formulation based on rice husk nanobiosilica embedded in a seed coating alginate film
Source: Front Plant Sci. 2024 May 21;15:1349573. doi: 10.3389/fpls.2024.1349573 (PMC11148368; doi:10.3389/fpls.2024.1349573)
Supplement: Supplementary file 11 [file DataSheet_3.pdf]

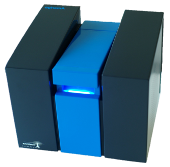

Measurement Report *Sample Name : SiNPs pleava*

Measurement Date: *18.08.2023 15:32* ***by:*** *DefaultExpert*

File Name : *SiNPs pleava_20230818_161921.dat* ***Report Date:*** *19.08.2023 15:28* ***by:*** *DefaultExpert*

comment:

SOP : DefaultSOP

Solvent ***:*** *Water* ***Refractive index*** ***:*** *1.33* ***Viscosity(mPa.s) at 27.57 °C*** ***:*** *0.8384*

Device ***:*** *AmeriQ* ***Wavelength*** ***:*** *638 nm* ***Angle :*** *170°*

Laser power ***:*** *10 %* ***Temperature set by*** ***:*** *Sensor*

Algorithm ***:*** *Cumulants/Pade Laplace/SBL*

Scattering Model : *Rayleigh* ***Particle refractive Index (a.u.) :*** *1.56 (Real) 0.01 (Imaginary)*

Experiment ***:*** *Mono-Acquisition*

Overview

From (hh:mm:ss)	To (hh:mm:ss)	Duration (hh:mm:ss)	T (°C)	Viscosity (mPa.s)	Laser Power (%)	Beta (a.u.)	Count Rate (kcps)
00.2 s	45:03.6	45:03.4	27.52-27.8	0.8341 - 0.8394	10	0.85	91 - 219

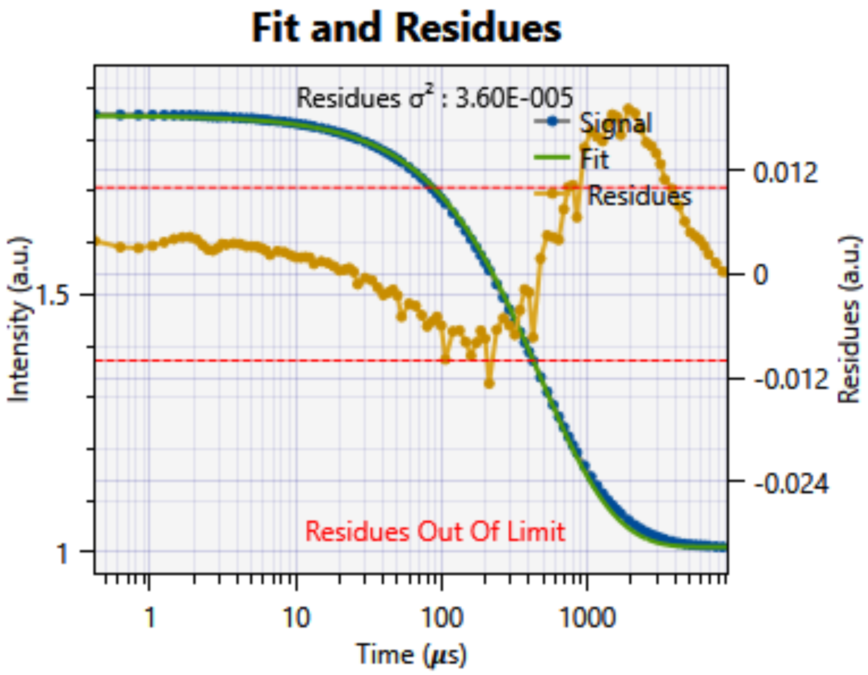

From (hh:mm:ss) : 00.2 s **To (hh:mm:ss) : 45:03.6** **Duration (hh:mm:ss) : 45:03.4**
Beta (a.u.) : 0.8

Z average (nm)	PDI (a.u.)	Std Dev (%)	Diffusion Coeff (m²/s)	Decay Rate (s-1)
**282.39**	**0.2785**	**0.53**	**1045.51**	**18.65194E-013**

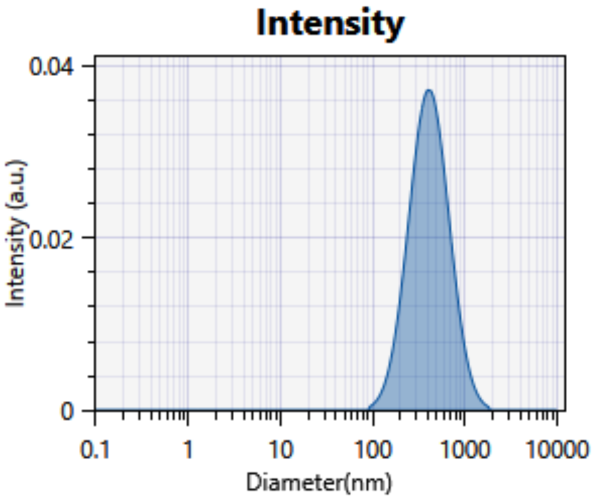

Di 10% : 215.44 nm Di 50 % : 411.58 nı
Di 90 % : 786.29 nm Mean : 460.16 nm

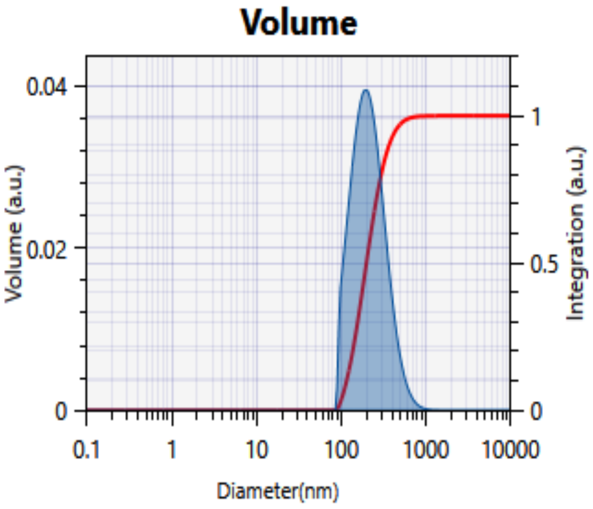

Dv 10% : 118.11 nm Dv 50 % : 205.71 nm
Dv 90 % : 375.23 nm Mean : 231.57 nm

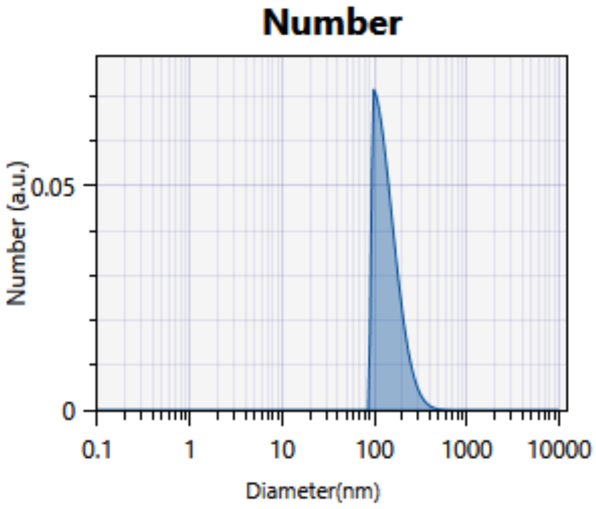

Dn 10% : 98.17 nm Dn 50 % : 129.55 nm
Dn 90 % : 205.71 nm Mean : 144.24 nm

Pade Laplace *SiNPs pleava SiNPs pleava_20230818_161921.dat*

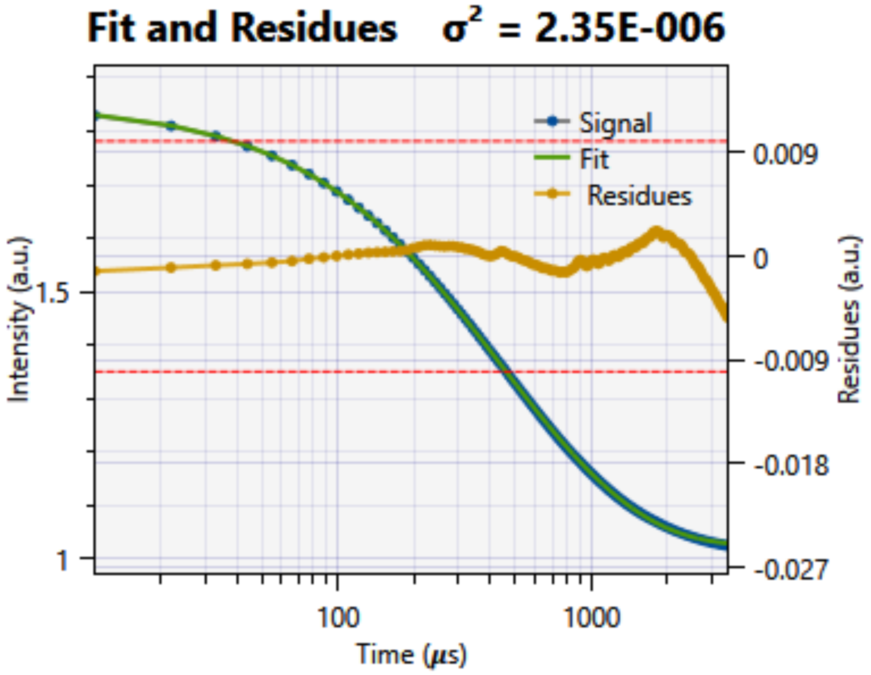

From (hh:mm:ss) : 00.2 s **To (hh:mm:ss) : 45:03.6** **Duration (hh:mm:ss) : 45:03.4**
Tau (µs) : 10.9908252740974 **Channel : 316**
Beta (a.u) : 0.8

Detected Size

Size (nm)	Intensity (a.u)	Volume (a.u)	Number (a.u)	Decay Rate (s-1)	Diffusion Coeff (m²/s)
109.71	0.23	0.95	1	2691.15	48.01004E-013
436.26	0.77	0.05	8.0e-4	676.76	12.07348E-013

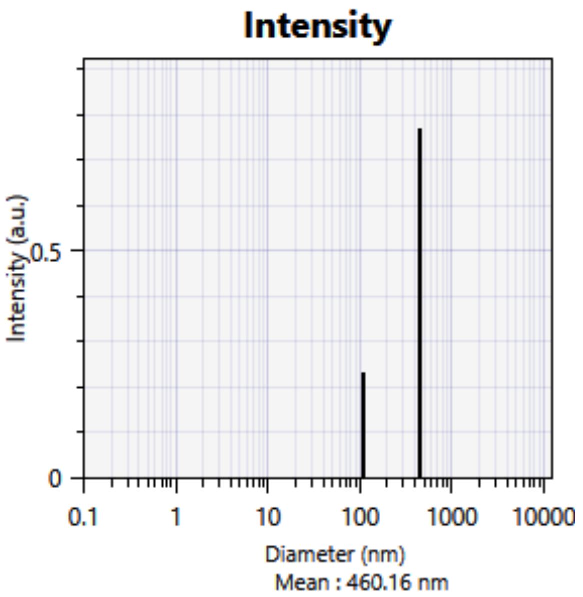

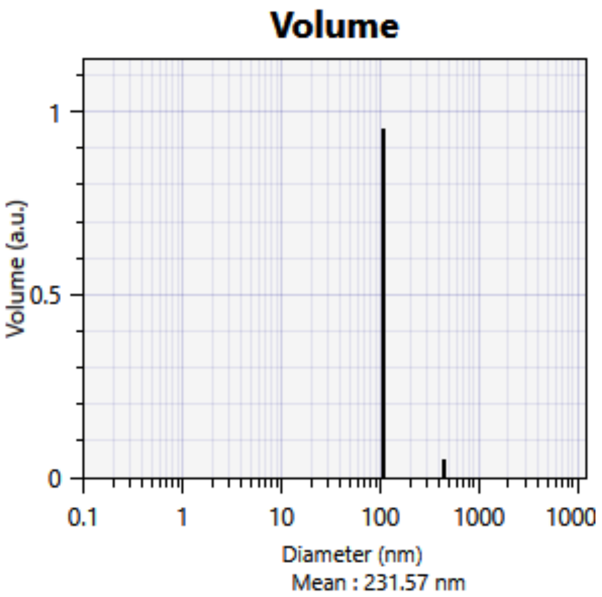

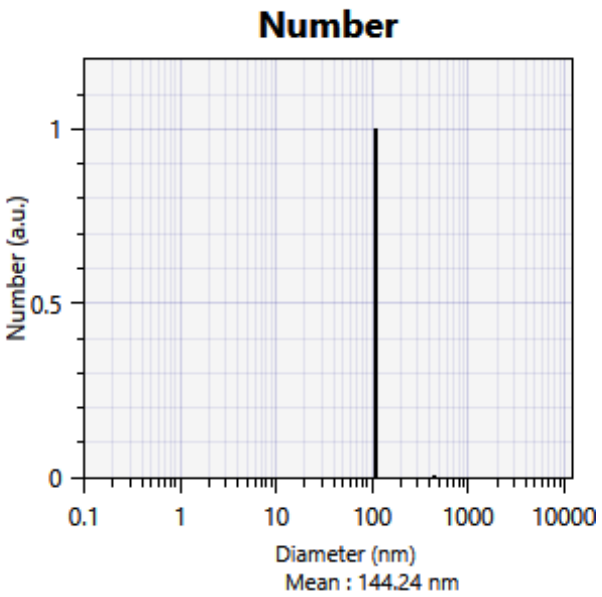

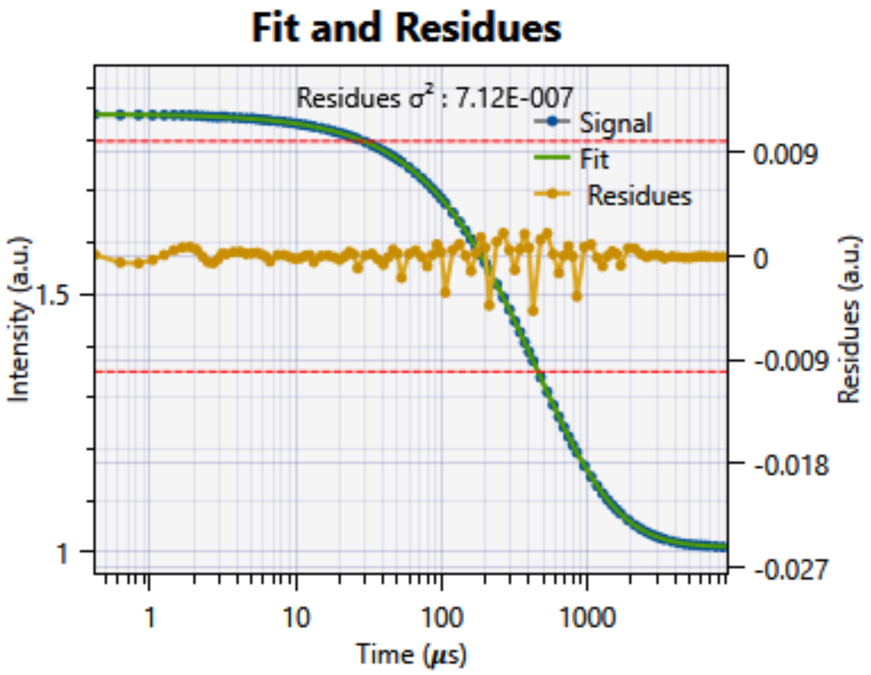

From (hh:mm:ss) : 00.2 s **To (hh: mm:ss) : 45:03.6** **Duration (hh: mm:ss) : 45:03.4**
Beta (a.u): 0.8

Mode (nm)	Mean (nm)	Std Dev (%)	Intensity (%)	Decay Rate (s-1)	Diffusion Coeff (m²/s)
225.64	552.79	137.97	100	1308.48	19.12016 E-013

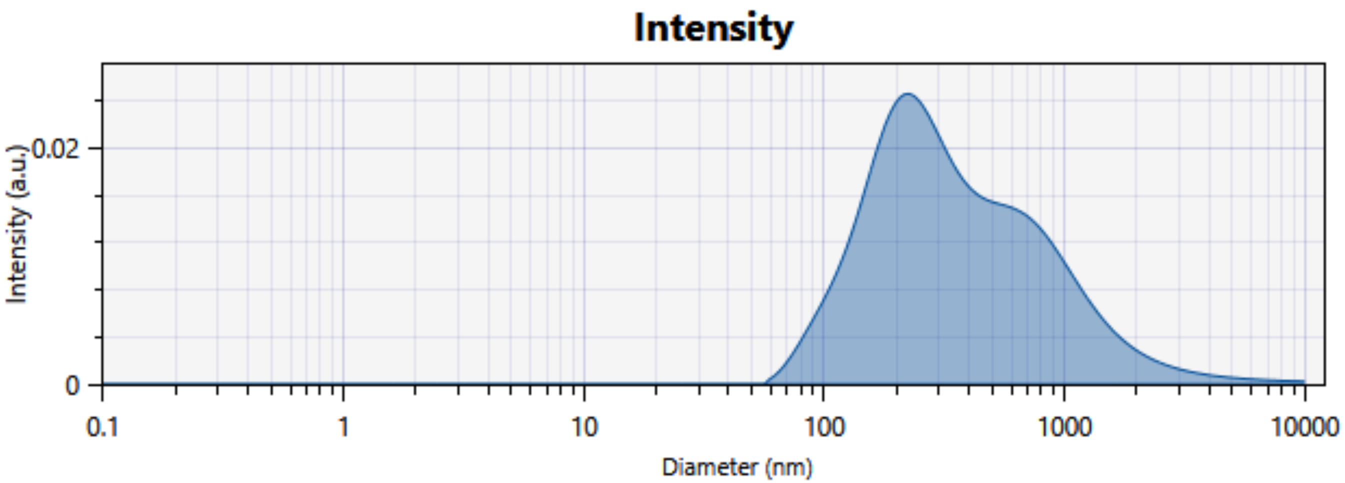

Di 10 % : 129.55 nm Di 50 % : 311.87 nm Di 90 % : 1138.22 nm Mean : 550.06 nm

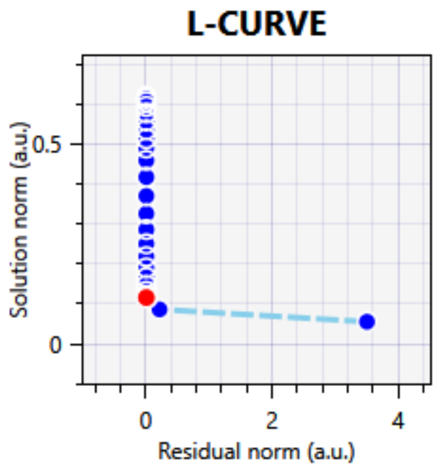

Best Solution : 4

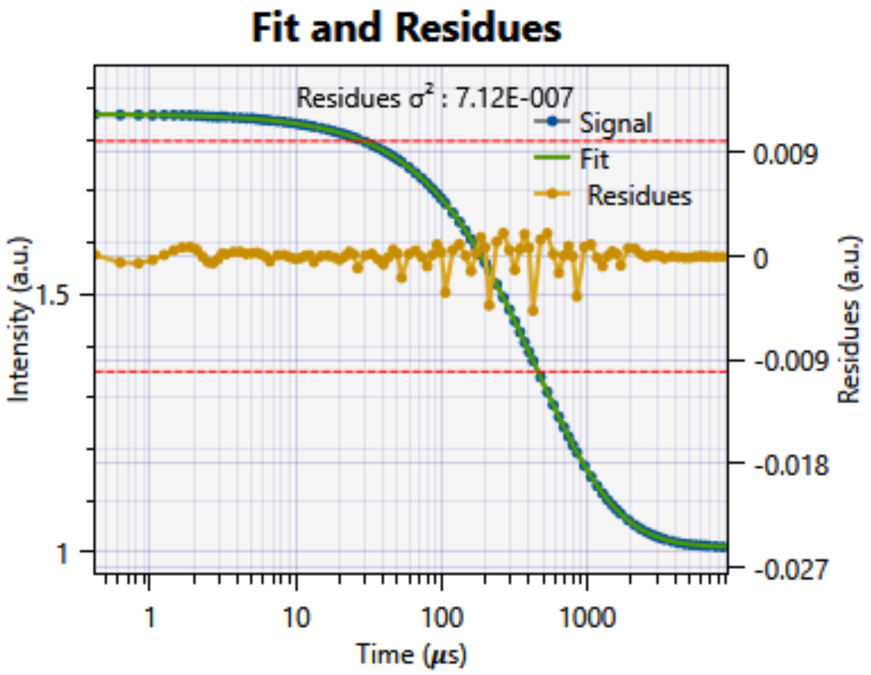

From (hh:mm:ss) : 00.2 s **To (hh:mm:ss) : 45:03.6** **Duration (hh:mm:ss): 45:03.4**
Beta (a.u): 0.8

Mode (nm)	Mean (nm)	Std Dev (%)	Volume (%)	Decay Rate (s-1)	Diffusion Coeff (m²/s)
89.5	130.72	56.43	100	3298.92	48.20564 E-013

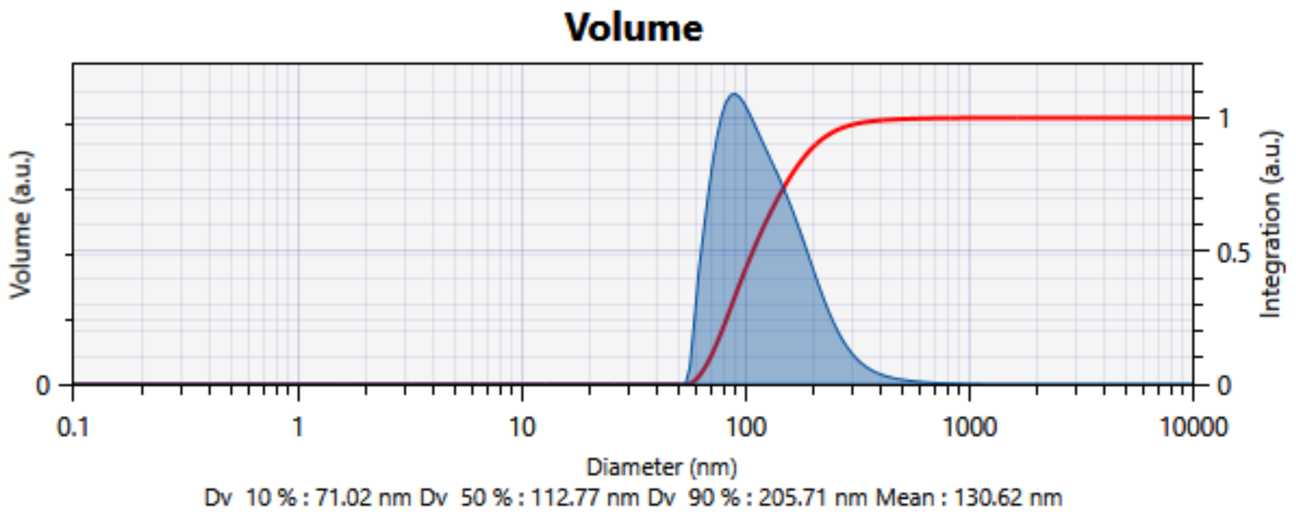

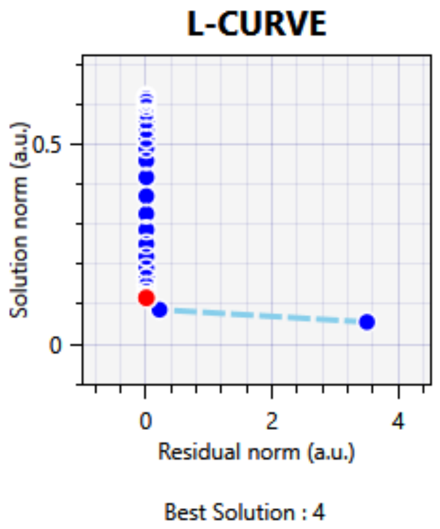

Fit and Residues

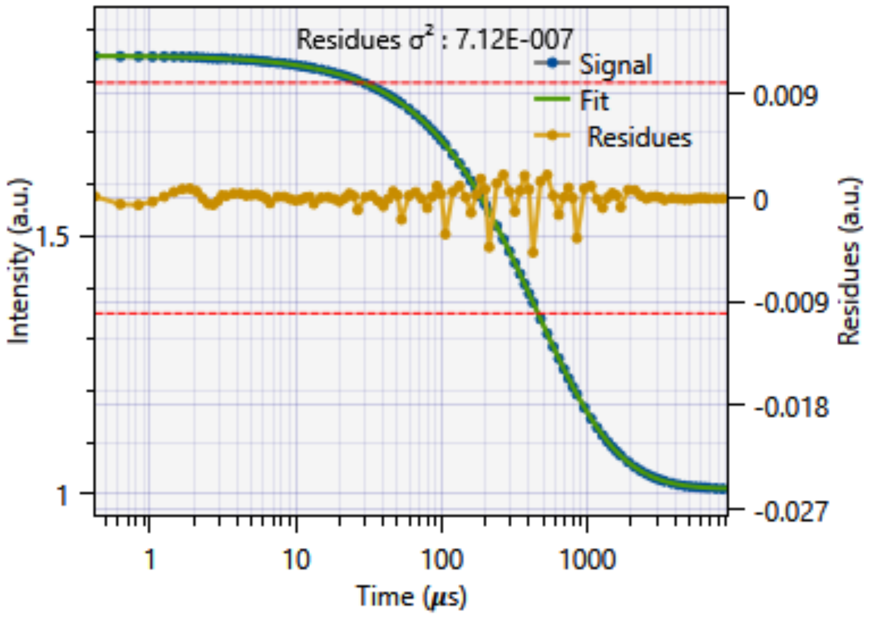

From (hh:mm:ss) : 00.2 s To (hh:mm:ss) : 45:03.6 Duration (hh:mm:ss) : 45:03.4
Beta (a.u) : 0.8

Mode (nm)	Mean (nm)	Std Dev (%)	Number (%)	Decay Rate (s-1)	Diffusion Coeff (m²/s)
71.02	86.21	31.9	100	4156.94	60.74345 E-013

Number

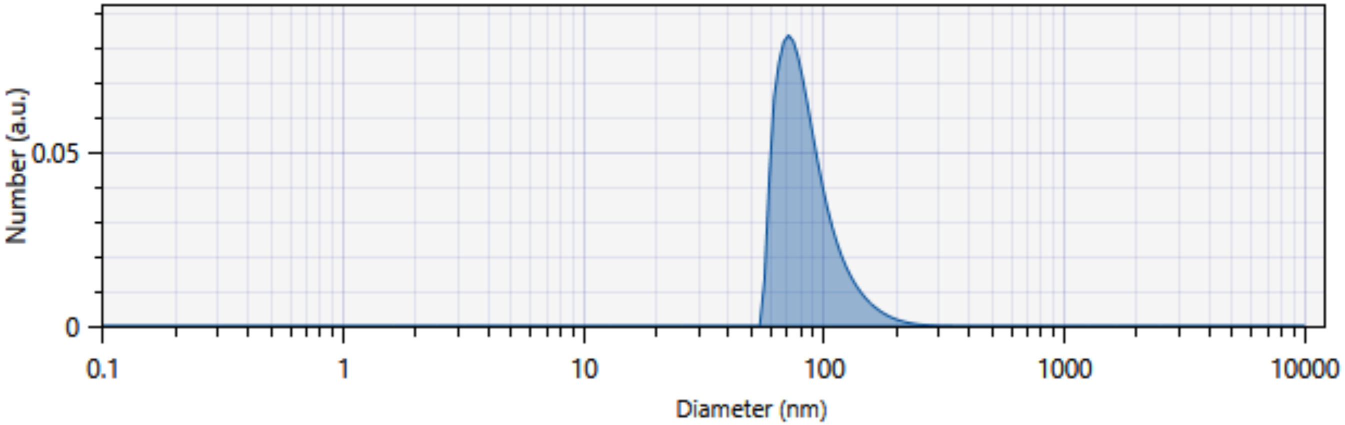

Dn 10 % : 61.83 nm Dn 50 % : 77.91 nm Dn 90 % : 118.11 nm Mean : 86.15 nm

L-CURVE

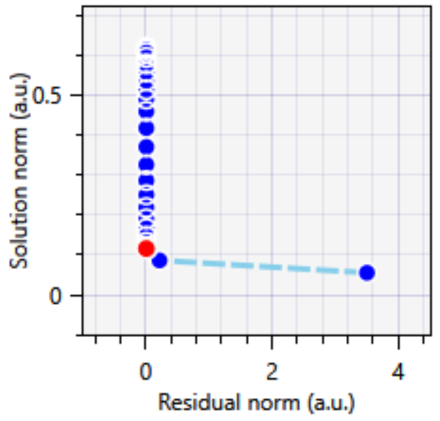

Best Solution : 4
